# Within-country age-based prioritisation, global allocation, and public health impact of a vaccine against SARS-CoV-2: A mathematical modelling analysis

**DOI:** 10.1016/j.vaccine.2021.04.002

**Published:** 2021-05-21

**Authors:** Alexandra B. Hogan, Peter Winskill, Oliver J. Watson, Patrick G.T. Walker, Charles Whittaker, Marc Baguelin, Nicholas F. Brazeau, Giovanni D. Charles, Katy A.M. Gaythorpe, Arran Hamlet, Edward Knock, Daniel J. Laydon, John A. Lees, Alessandra Løchen, Robert Verity, Lilith K. Whittles, Farzana Muhib, Katharina Hauck, Neil M. Ferguson, Azra C. Ghani

**Affiliations:** a MRC Centre for Global Infectious Disease Analysis, and the Abdul Latif Jameel Institute for Disease and Emergency Analytics, School of Public Health, Imperial College London, St Mary's Campus, Norfolk Place, London, W2 1PG, United Kingdom; b PATH, 455 Massachusetts Avenue NW, Suite 1000, Washington, DC 20001, USA; c Department of Infectious Disease Epidemiology, London School of Hygiene & Tropical Medicine, Keppel St, Bloomsbury, London WC1E 7HT, United Kingdom

**Keywords:** SARS-CoV-2, Mathematical model, COVID-19, Vaccination model, Optimisation

## Abstract

The worldwide endeavour to develop safe and effective COVID-19 vaccines has been extraordinary, and vaccination is now underway in many countries. However, the doses available in 2021 are likely to be limited. We extend a mathematical model of SARS-CoV-2 transmission across different country settings to evaluate the public health impact of potential vaccines using WHO-developed target product profiles. We identify optimal vaccine allocation strategies within- and between-countries to maximise averted deaths under constraints on dose supply. We find that the health impact of SARS-CoV-2 vaccination depends on the cumulative population-level infection incidence when vaccination begins, the duration of natural immunity, the trajectory of the epidemic prior to vaccination, and the level of healthcare available to effectively treat those with disease. Within a country we find that for a limited supply (doses for < 20% of the population) the optimal strategy is to target the elderly. However, with a larger supply, if vaccination can occur while other interventions are maintained, the optimal strategy switches to targeting key transmitters to indirectly protect the vulnerable. As supply increases, vaccines that reduce or block infection have a greater impact than those that prevent disease alone due to the indirect protection provided to high-risk groups. Given a 2 billion global dose supply in 2021, we find that a strategy in which doses are allocated to countries proportional to population size is close to optimal in averting deaths and aligns with the ethical principles agreed in pandemic preparedness planning.

## Highlights

• The global dose supply of COVID-19 vaccines will be constrained in 2021.
• Within a country, prioritising doses to protect those at highest mortality risk is efficient.
• For a 2 billion dose supply in 2021, allocating to countries according to population size is efficient and equitable.

## 1 Introduction

Even as countries begin to introduce approved SARS-CoV-2 vaccines [1], [2], [3], [4], [5], [6], the demand for doses is likely to exceed supply throughout 2021. Despite international initiatives such as COVAX to share the risks of the research and development process, and ensure equitable access [7], political and economic incentives for countries to prioritise national interest remain high – as has already been demonstrated by countries stockpiling treatment supplies and other pharmaceuticals [8], [9] as well as countries signing advanced vaccine purchase agreements. Four allocation principles, reflected in the World Health Organization (WHO) global allocation framework [10], have been identified by bioethicists to guide allocation of scarce resources: (A) that the health benefits of the resource are maximised; (B) that priority is given to those who would be worst-off in the absence of the resource; (C) that individuals in equal circumstances should be treated equally; and (D) that societal benefit is maximised [11], [12]. A key component in achieving principles A and B will be targeting the vaccine to those at highest risk of death. Given the strong age-gradient of risk associated with COVID-19 infection [13], allocation is likely to be age-targeted. However, to meet principle B, vaccine dose allocation will need to be balanced against both life-expectancy and the additional variation in the risk of death resulting from, for example, inequitable access to healthcare globally. In addition, aligned with principle D, essential workers such as those providing the frontline health response may need to be prioritised [14]. Adhering to these principles to derive a fair and optimal allocation strategy, given limited vaccine stocks, is far from straightforward.

We extend a model of SARS-CoV-2 transmission to explore the public health impact of vaccine characteristics, epidemic stages, and population-targeting strategies. We apply the model to countries with different levels of income to understand the impact of demographics, societal mixing, and health system constraints in overall vaccine benefit. We explore the implications of these characteristics for within-country and global allocation and quantify the maximum public health benefit of different allocation strategies under a range of supply constraints.

## 2 Methods

### 2.1 Mathematical model

We extended a previously developed age-structured deterministic SEIR-type compartmental model of SARS-CoV-2 transmission [15] to include vaccination. The model explicitly incorporates the clinical pathway for those requiring hospitalisation, allowing estimates of the need for oxygen and/or intensive care support. Transmission depends on age-based contact matrices and a constant transmission rate per contact. Other risk groups or settings (such as healthcare workers or care homes) are not included. The model was extended to capture loss of naturally-acquired immunity.

Once vaccination is introduced into a population, we assume that all eligible individuals (depending on the targeting or prioritisation that is applied) are vaccinated at a constant rate, up to a maximum level of coverage. Vaccination prioritisation strategies are modelled by specifying an age disaggregated matrix of coverage targets where rows are ordered prioritisation steps (Supplementary Information, Section 1.2). Susceptible, latent, or recovered individuals can be vaccinated. Vaccinated individuals initially enter a temporary state to capture the delay between receiving the vaccine and being protected before moving into a vaccine-protected state. We model a vaccine that is efficacious against either infection or disease, or that is efficacious against infection with additional reduction in the risk of severe disease in vaccinated individuals who experience a breakthrough infection (the default assumption). We assume that protection is partial, with efficacy parameters detailed in Table 1. Additional details are in the Supplementary Information (Figure S1; Tables S1 and S2).

**Table 1:** **Summary of Scenarios Explored**. The values in bold represent the default parameters, unless otherwise stated.

Parameter	Values	References / Notes
Income setting	**High-income (HIC);** Upper-middle income (UMIC); Lower-middle income (LMIC); Low-income (LIC)	[16]
Transmission from March 2020 - April 2020 (R_0_)	**2.5;** 1.5–4.0	Assumed values to mimic patterns of epidemics observed globally
Transmission from May 2020 - January 2021 (R_t1_)	**1.1;** 0.9; 1.3	
Transmission from February 2021 onwards (R_t2_)	**2.0**; 1.5; 2.5	
Vaccine mode of action	**Combined;** Infection; Disease	[2], [3], [4], [5], [6], [29]
Vaccine efficacy against infection	**90%**; 50–90%; 0% (disease-only efficacy)	[3], [4], [5], [6]
Vaccine efficacy against disease	**Additional 60% for breakthrough infections**; 50–90% (disease-only efficacy); 0% (infection-only efficacy)	[3], [5], [25], [26], [27], [28]
Reduction in efficacy against infection in individuals 65 years and older due to immunosenescence	**0%**; 50%	[44], [45]
Vaccine coverage	**80%;** 0%–100%	[58]
Vaccine duration of protection	**Long-term**; 1 year; 6 months	[38]
Duration of naturally-acquired immunity	**1 year**; lifelong; 6 months	[35], [36], [37], [59], [60]
Age targeting	**All ages targeted;** Older; Younger; combinations of 5-year groups	Chosen to reflect different patterns being adopted by countries
Duration between vaccination and vaccine protection following second dose	**7 days**	[30], [31], [32], [33], [34]
Health system constraints	**Present;** Absent	[15]
Health system constraint assumptions	**Health system constraints present** (LIC: Capacity limited + negative health outcomes due to poorer standard of care LMIC: Capacity limited UMIC & HIC: Capacity unlimited as surge capacity is put in place); Health system constraints absent (Capacity unlimited in all settings)	[15]
Vaccine dose supply constraint	**2 billion doses in 2021** (COVAX); 2 billion doses in 2021 + direct country procurement (1.15 billion doses by HICs, 1.10 billion doses by MICs)	[7]
Dose schedule	**2 doses;** 1 dose	[2], [4], [5], [6]
Vaccine buffer stock and wastage allowance	**15%**	[42]

The model code is available as an R package at https://github.com/mrc-ide/nimue. To aid utility of the modelling framework by policymakers, government agencies, and other stakeholders, we additionally developed a user-friendly interface (Box 1). In the interface the model is fitted to country epidemics (Supplementary Information, Section 1.4) with fits updated weekly. Updates will incorporate new information that emerges as the pandemic unfolds.

Box 1 **COVID-19 Scenario Analysis Tool.**

**Table:** 

A user-friendly interface to the model used here is freely available at www.covidsim.org. The interface allows the user to explore the impact of vaccine introduction on the SARS-CoV-2 epidemic in any given country.
Key Features:
1. The interface pre-loads epidemic curves fitted to epidemiological data (reported cases and deaths) in each country [24]. These fits are updated weekly. This allows the user to explore scenarios based on the current state of the epidemic.
2. Forward projections of the epidemic in the absence of vaccination (counterfactual scenarios) can be flexibly generated by specifying the reproduction number, R, in the absence of immunity. This allows the user to explore the potential to relax NPIs as vaccine roll-out increases. Healthcare capacity (general beds and critical care beds) are incorporated (and can be modified by the user) to explore the projected impact on healthcare demand.
3. Country-specific data on population size, demography, representative mixing patterns, numbers of health-care workers and the size of at-risk groups are incorporated to allow exploration of different vaccine prioritisation strategies specific to the local setting [15], [17], [57], [61].
4. Vaccine properties can be varied by the user such that the impact of different approved vaccines can be generated and modified as further data emerge.
5. Supply constraints can be incorporated by specifying the vaccine courses available as a percentage of the population.
6. Delivery can be specified by modifying the number receiving the first dose each week and selecting one of four prioritisation scenarios closely aligned with current WHO recommendations (HCW and Elderly; HCW, Elderly, High-Risk Groups; Elderly; No Prioritisation).
7. Vaccine uptake can be modified.
8. All outputs can be exported to text files for further analysis.
9. Additional features may be added as the pandemic unfolds to best capture trends and new scientific knowledge.

### 2.2 Parameterisation

We stratify the global population into four groups, based on the current World Bank [16] classification of high-income countries (HIC), upper-middle-income countries (UMIC), lower-middle-income countries (LMIC) and low-income countries (LIC). For each income setting, the age distribution of the population and age-based contact patterns are modified to capture epidemiological characteristics of countries in the different income strata. We use the current age distribution for the country with the median GDP in each of the four groups (HIC: Malta; UMIC: Grenada; LMIC: Nicaragua; and LIC: Madagascar) [17], and representative age-based contact patterns based on the availability of contact data studies [15]. Natural history parameters for SARS-CoV-2 infection are based on a review of published estimates and large clinical studies [18], [19] with age-stratified probabilities of requiring hospitalisation and intensive care obtained from a national study of French hospital admissions [20] and the age-stratified infection fatality ratio (IFR) obtained from a recent meta-analysis [21] (Table S2). The model has been previously validated by using Bayesian methods to fit to country-specific data on COVID-19 deaths (Figure S2) [22], [23], [24]. We consider that health systems may be constrained in LMIC and LIC settings, and that once exceeded, those requiring hospitalisation but who do not receive it experience worse outcomes, following Walker et al [15], while in UMIC and ad HIC settings, surge capacity may be implemented to meet hospitalisation requirements (Supplementary Information Section 1.5). Our default assumption is that health system constraints are in place.

Interim efficacy estimates against symptomatic COVID-19 have been published for several candidate vaccines, including Pfizer/BioNTech (95% efficacy), Moderna (94.1%), Oxford/AstraZeneca (63.1% for two standard doses), and the Gamaleya Center (91.6%) [3], [4], [5], [6]. Trial results indicate higher protection against severe COVID-19 disease, and this is supported by evidence being generated by vaccine implementation in the UK and Israel [3], [5], [25], [26], [27], [28]. The extent to which these vaccines will prevent infection is not fully known, however the phase III Oxford/AstraZeneca study reported preliminary efficacy against asymptomatic COVID-19 of 27.3% (95% CI − 17.2–54.9) [2], and evidence from the UK indicates a four-fold reduction in asymptomatic COVID-19 in vaccinated versus unvaccinated healthcare workers following a single Pfizer/BioNTech vaccine dose [29]. We therefore assume efficacy against infection of 90%, with an additional 60% efficacy against severe disease for vaccinated individuals who experience breakthrough infection [25]. We assume an average period between vaccination and protection of 7 days [30], [31], [32], [33], [34], and no partial protection in this period. Given that the duration of protection after vaccination is unknown, we assume that vaccine-induced immunity is either long-term, one year, or six months. Antibody reversion from natural infection [35] and re-infection [36] has now been reported, and a UK-based study of hospital staff found that prior infection was associated with an 83% lower risk of infection, with a median duration of follow-up of five months [37]. We therefore assume that natural immunity following infection persists for an average of one year, and additionally explore both six-month and long-term durations [38]. Parameters are summarised in Tables 1, S1, and S3.

### 2.3 Scenarios and counterfactual

To evaluate the public health impact of any vaccine, we must consider the epidemic trajectory in the absence of vaccination. However, because of the vastly different experiences between countries in their exposure and response to SARS-CoV-2, considering such counterfactuals is challenging, particularly since testing practices differ markedly, making it difficult to accurately assess the current epidemic stages of different countries. Furthermore, while vaccination has commenced in some countries, scale-up will occur over a longer period.

**Fig. 1:**
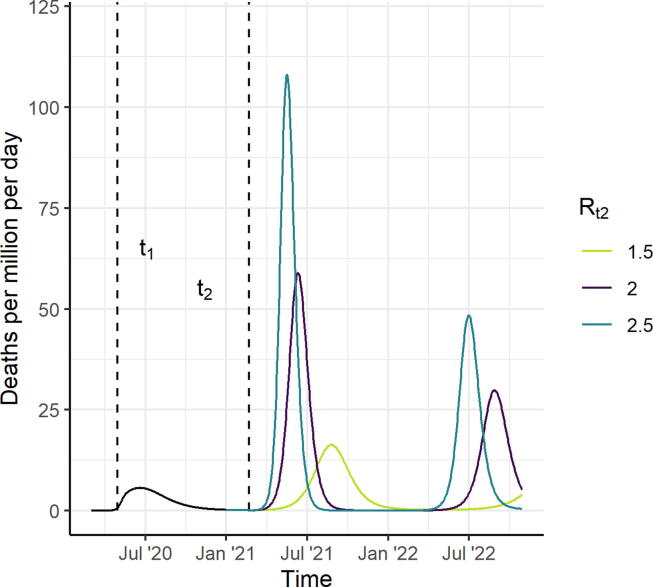
**Scenarios for the Course of the Epidemic from 2020 to 2022, for a High-Income Country Setting, in the Absence of a Vaccine (counterfactual scenarios).** We assume that R_0_ = 2.5 up to time t_1_ (May 2020) and that R_t1_ drops to 1.1 between time t_1_ and t_2_ (February 2021). Assuming an average duration of naturally-acquired immunity of one year, this results in 11% in the recovered (immune) state at vaccine introduction. From time t_2_ onwards, we consider three counterfactual scenarios, R_t2_ = 1.5, 2 and 2.5 shown in light green, purple, and turquoise, respectively. Vaccine impact is compared to these counterfactual scenarios. (For interpretation of the references to colour in this figure legend, the reader is referred to the web version of this article.)

Modelled levels of transmission are determined by the time-varying reproduction number, R_t_. We do not explicitly model individual NPIs, but instead incorporate the assumed impact of combined NPIs within the estimate of the reproduction number. Our baseline scenarios assume that an initial peak in transmission occurred in the first half of 2020, which is reduced by NPIs, resulting in lower levels of transmission through the remainder of the year (Figure S3). Assuming a mean duration of immunity of one year and for levels of transmission where R_0_ = 2.5 and R_t1_ = 1.1, the simulation results in 11% of the population becoming immune by the end of 2020 in a HIC setting (plausible given seroprevalence surveys [23], [24], and 11%, 12%, and 16% immune in UMIC, LMIC and LIC settings respectively (Fig. 1; Figure S5). We explore additional scenarios with long-term natural immunity and different plausible values of R_0_ and R_t1_ (Figures S5 and S6).

To understand the public health value of the vaccine and consider allocation strategies that do not penalise countries for lacking the capacity to implement NPIs, we make the simplifying assumption that the vaccine is introduced while NPIs are in place. This avoids the situation in which our modelled results suggest the vaccine has no impact because vaccine introduction “misses” the counterfactual epidemic. NPIs are then lifted (R_t_ increases) after the target coverage is achieved, allowing vaccine impact in a fully vaccinated population to be compared to the counterfactual scenario. Under this simplification, we consider a scenario where the first vaccine dose is introduced from the beginning of 2021 over 30 days, and that a second dose occurs 21 days after the first, allowing target coverage to be achieved by the end of February 2021 and with no subsequent vaccination (although in practice if vaccine-induced immunity wanes, then repeat vaccination is likely). In sensitivity analyses we also consider a more realistic coverage scale-up throughout the year. It is unlikely that relaxing NPIs would result in a return to the high level of transmission (R_0_ between values of 2 and 4) seen before controls were introduced, due to ongoing interventions [24]. We therefore explore three scenarios for R_t2_ from March 2021 onwards: R_t2_ = 1.5, 2, or 2.5. These values were chosen to represent a range of epidemic trajectories in the absence of vaccination, with the higher R_t2_ resulting in a rapid epidemic and the lower R_t2_ values resulting in flatter but longer epidemics (Fig. 1).

We compare scenarios from March 2021 onwards and quantify vaccine impact over 2021 only and 2021–2022. We calculate the deaths averted and life-years gained as the differences in deaths and life-years lost between the vaccinated and counterfactual scenarios. The life-years lost is estimated by summing the remaining life expectancy for each death in each age group, where the reference life expectancy is 86.6 years [39], consistent with ethical vaccine allocation approaches [40].

### 2.4 Vaccine allocation

We first consider optimal vaccine allocation within a country under different supply constraints. To do this, we divide the population into 5-year age groups and generate all possible age group combinations, retaining the combinations in which up to two contiguous “bands” of age groups could be selected, where the selected age groups would then be allocated the vaccine (Supplementary Information Section 1.7; Figure S4). For each combination we calculate the deaths averted in 2021, assuming 80% uptake within each 5-year age group. The vaccine supply is calculated as the percentage of the total population who is vaccinated. We then select the most efficient allocation frontier from these simulations (Figure S11). We compare this optimal strategy to two age-targeted approaches. In the first approach we sequentially allocate from the oldest age group downwards (80 + years, 75+, 70 + etc.), for both uniform vaccine efficacy across age groups and where efficacy in the 65 + group is reduced by 50%. In the second approach we target the working-age population, beginning with 60–64 years and working downwards by age, then sequentially including the younger and older age groups until the entire population is covered. We repeat this analysis for each income setting.

Second, we explore the impact of different global vaccine dose allocation strategies, assuming a two-dose schedule, that the dose supply is constrained to the two billion doses that will be made available through the COVAX facility in 2021 [41], and 15% buffer stock and wastage [42]. We simulate the total global health impact for each of six plausible identified strategies:

• Strategy A: Countries allocated doses relative to population size.
• Strategy B: Countries allocated doses relative to population size, with individuals 65 + years targeted first.
• Strategy C: Countries allocated doses relative to size of population 65 + years, with 65 + years targeted first.
• Strategy D: High-income countries can access doses first.
• Strategy E: Low- and lower-middle-income countries can access doses first.
• Strategy F: Countries allocated the first 2 billion doses relative to population size, with additional doses available to high- and middle-income (UMIC and LMIC) countries (1.15 billion and 1.10 billion respectively, distributed according to population).

For these six strategies, we do not stratify the population within an income setting into individual countries, therefore by extension we assume that available doses can be distributed across any number of countries within an income setting, even if coverage within an individual country is low.

We additionally use the ompr package in R [43], which formulates and solves mixed linear integer optimisation problems, to identify the optimal global vaccine allocation strategy. To do this, we stratify the global population into the population sizes of the 85 largest countries (comprising 95% of the global population), assigning each country the characteristic age distribution, contact matrix, and health system capacity for its income setting to reduce computational requirements. We again calculate the deaths averted in 2021 for the set of 5-year age group combinations. Under this optimisation algorithm, the available doses can be allocated both between and within countries, thereby allowing different combinations of 5-year age groups to be selected within each country.

For both the fixed and optimal global vaccine allocation strategies, we calculate the total deaths averted per million population, and the deaths averted per 100 fully vaccinated persons, to compare the efficiency of the identified strategies.

### 2.5 Sensitivity analysis

We identify the optimal within-country and global between-country vaccine allocations, and calculate the impact of the six fixed global strategies (A–F), while varying our default parameter assumptions: vaccine efficacy reduced from 90% to 70% [38]; efficacy in the 65 + population reduced by 50% [44], [45]; vaccine efficacious against disease only; higher NPIs following the introduction of a vaccine (R_t2_ reduced from 2.0 to 1.5); reduced NPIs following the introduction of a vaccine (R_t2_ increased from 2.0 to 2.5); health system constraints absent; infectiousness in children younger than 10 years reduced by 50%; and life-years gained rather than deaths averted as the optimisation outcome measure.

## 3 Results

**Fig. 2:**
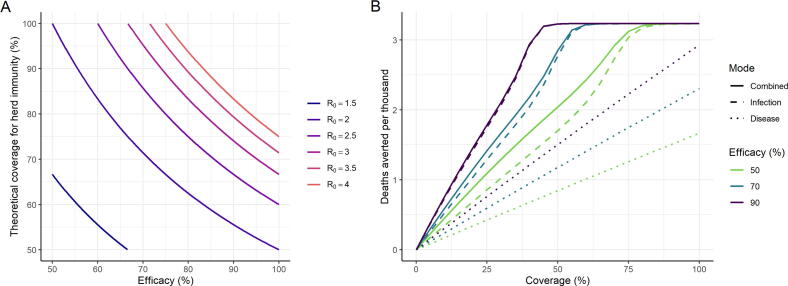
**Vaccine Efficacy and Herd Immunity.** (A) The relationship between efficacy of a vaccine that reduces the risk of infection, and the theoretical coverage required for herd immunity, for a range of levels of transmission shown as the level of the reproduction number R_0_. The theoretical coverage assumes random mixing of the population. (B) Projected total deaths averted per thousand population in 2021 under the default assumptions shown in Table 1 (with R_0_ = 2.5, R_t1_ = 1.1, and R_t2_ = 2.0). The colours show different magnitudes of vaccine efficacy . Solid lines represent impact for a vaccine that is efficacious against infection, with additional efficacy against severe disease. Dashed lines represent a vaccine that is efficacious against infection only, and dotted lines represent a vaccine that only prevents severe disease (and hence death) but does not reduce infection or onwards transmission (Table S3). Impact is shown for a HIC setting and all age groups are vaccinated uniformly; additional plots for other income settings and health system constraint assumptions are in Figure S7.

Vaccines that are efficacious against infection can have health impact by reducing the burden of disease through direct protection of those vaccinated, and by reducing transmission, thus providing indirect protection to the entire population. For our assumed R_0_ of 2.5, the theoretical coverage required to achieve herd immunity for a 100% efficacious vaccine is 60%, in a population that mixes randomly. For a more transmissible strain (such as the recently identified UK variant [46], [47]) with an R_0_ of 4 the coverage required for herd immunity increases to 75%. Coverage must be higher if vaccine efficacy is below 100% (Fig. 2A). For example, for a vaccine with 90% efficacy the coverage would need to be 67% for R_0_ = 2.5 and 83% for R_0_ = 4. However, as demonstrated in our simulations, temporary herd immunity can be reached at lower coverage levels since ongoing NPIs generate a lower effective reproduction number (illustrated here with R_0_ = 2). With wider vaccine availability from 2022 onwards it is likely that NPIs would gradually be lifted, and hence higher coverage would be required to sustain herd immunity.

A vaccine that prevents infection has a greater impact than one of equivalent efficacy that prevents severe disease only due to the indirect effect of the former on transmission (Fig. 2B). With an infection-reducing vaccine the projected deaths averted increases sharply with coverage until herd immunity is approached, at which point the curve plateaus. In contrast, a vaccine that only provides direct protection against disease has a linear relationship between coverage and health impact. For example, for R_0_ = 2.5, R_t1_ = 1.1, R_t2_ = 2.0, vaccine efficacy of 90% and coverage of 67%, only approximately 60% of the deaths averted by an infection-reducing vaccine would be averted by a disease-reducing vaccine; this finding is consistent across income settings (Figure S7). The additional value of indirect protection remains important for all potential vaccine efficacies and levels of coverage (Fig. 2B). The duration of protection will affect overall impact (Figure S8) although this could be overcome through repeat vaccination.

**Fig. 3:**
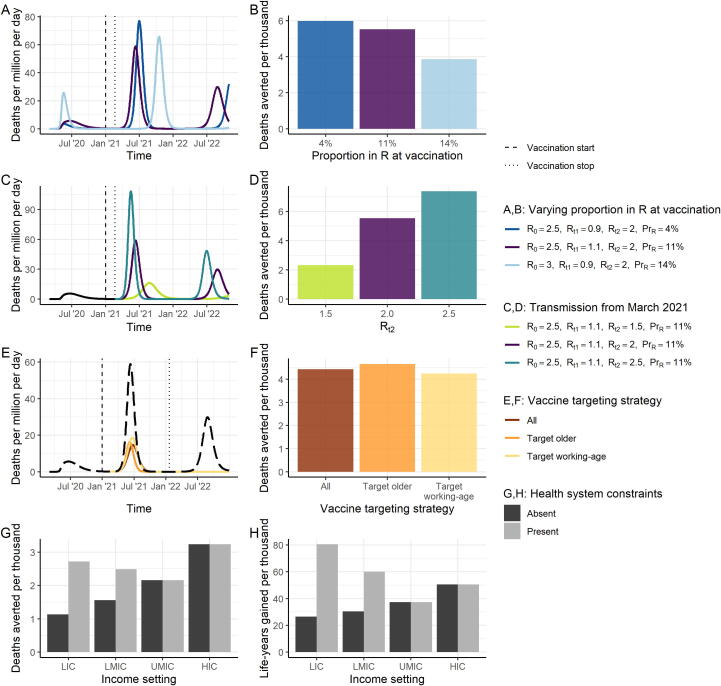
**Epidemic Characteristics at Vaccine Introduction.** (A) Three scenarios for the stage of the epidemic at vaccine introduction. The dark blue line shows a scenario where transmission has previously been suppressed and therefore the proportion immune at vaccine introduction is low (4%). The purple line shows the default scenario in which the proportion immune at vaccine introduction is 11%. The light blue line shows a scenario in which more widespread transmission occurred during 2020 and the proportion immune at vaccine introduction is higher (14%). (B) The projected impact of vaccination in terms of total deaths averted per thousand individuals over 2021–2022, for the scenarios in A. All other vaccine characteristics are set to the default assumptions. (C) Three scenarios for the course of the epidemic from February 2021 onwards assuming the default scenario up until this time of vaccine introduction. The light green line shows the scenario for R_t2_ = 1.5, purple R_t2_ = 2, and turquoise R_t2_ = 2.5. (D) Deaths averted per thousand individuals over 2021–2022, for the scenarios in C. All other vaccine characteristics are set to the default assumptions. (E) Three scenarios for the course of the epidemic from February 2021 onwards where NPIs are assumed to be lifted when the vaccine is introduced, and the target population is vaccinated at a constant rate over 2021, for three vaccine targeting strategies (coloured lines). The black long-dashed line shows the counterfactual scenario. (F) Deaths averted per thousand individuals over 2021–2022, for the scenarios in E. “All”: all age groups vaccinated simultaneously. “Target older”: the 80 + group is vaccinated first, then additional groups (75–79, 70–74 and so on) are consecutively vaccinated. “Target working-age”: the 15–64-year-old group is vaccinated first, and then the older group, and then children. (G, H) Deaths averted (G) and life-years gained (H) per thousand population in 2021 for each income setting, where health systems are either unconstrained (dark grey) or constrained (light grey). Default vaccine parameters are in Table 1. (For interpretation of the references to colour in this figure legend, the reader is referred to the web version of this article.)

The health benefit of vaccination will depend on the proportion of the population that have naturally-acquired immunity at the time of vaccine introduction and the duration of that immunity. There is a clear decrease in public health impact with higher pre-existing immunity in the population, under the assumption that ongoing NPIs in 2021 do not differ based on this immunity (Fig. 3A–B). In addition, if vaccination is timely, its impact is greater in countries where other NPIs are not sustained (Fig. 3C–D). While our main analysis assumed that the target population is vaccinated before NPIs are lifted, if NPIs are lifted at vaccine introduction and vaccination takes place over a longer period, impact will be lower (Fig. 3E–F and Figure S9). Furthermore, a greater public health impact will be obtained by initially targeting the elderly rather than the working-age population because the overall vaccine coverage during the period in which the epidemic occurs is lower, compared to if NPIs are lifted after the target population is vaccinated.

The public health value of vaccination will also depend on the risk profile of the population and whether other therapeutic means are available to reduce morbidity and mortality. In the absence of health system constraints, the impact of vaccination is predicted to be greatest in HIC since these countries have the largest elderly populations (Fig. 3G–H). For example, we predict 3.2 deaths averted per 1,000 population in a HIC compared to 1.1 in a LIC in 2021. However, once health system constraints are accounted for in lower-income settings, the public health value of the vaccine becomes more similar across settings. Furthermore, if the metric for assessing value accounts for the age at death (i.e. life-years gained) we predict a further shift towards benefiting the lowest income settings (Fig. 3H), with 81 life-years gained per 1,000 population in a LIC compared to 50 in a HIC in 2021.

**Fig. 4:**
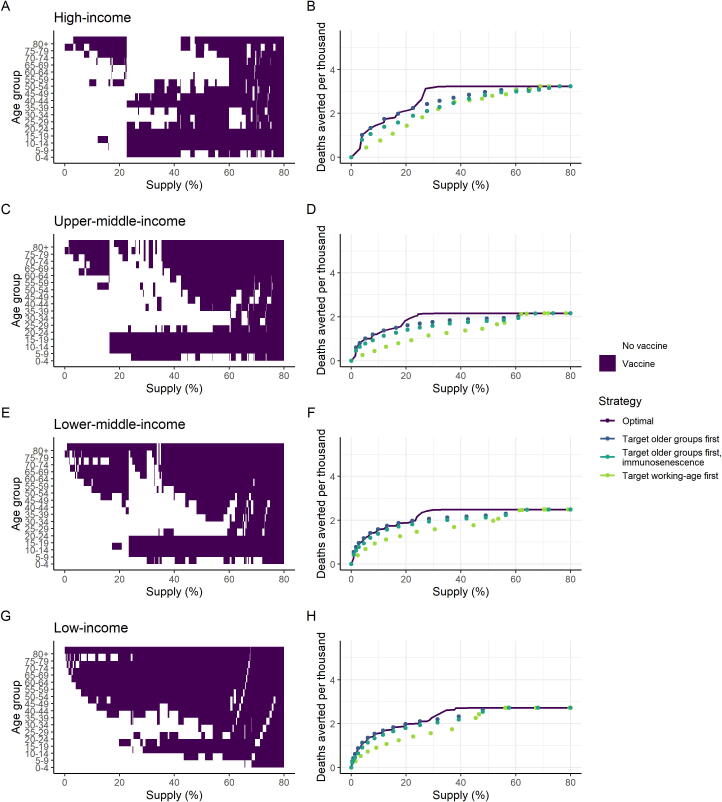
**Age-targeting of Vaccine Introduction.** These panels illustrate the most efficient allocation under different supply constraints, where the supply is defined as the proportion of the total population able to access two doses. Panels A, C, E and G show the age groups allocated the vaccine under the optimal strategy for different levels of vaccine supply, where the purple shaded regions indicate the age groups prioritised. Panels B, D, F and H show the total health impact expressed as deaths averted per thousand population as a function vaccine supply. The optimal strategies from the left-hand panels are shown in purple on the right-hand panels. The dark blue points show the strategy that prioritises the older at-risk age: 80 + for the lowest coverage level, and sequentially including additional age groups (75–79, 70–74 and so on) as additional doses are available. The turquoise points show the same strategy, but where vaccine efficacy in the 65 + population is 35% (immunosenescence). The green points show the strategy that prioritises the working-age population first (beginning with the 60–64 age group and sequentially adding younger groups), then vaccinates the elderly and children as doses become available. Health system constraints in LICs and LMICs are assumed to be present These allocations are generated using the default vaccine characteristics in Table 1, with 80% coverage in the target age group vaccinated; additional scenarios are shown in Figures S12–S19. (For interpretation of the references to colour in this figure legend, the reader is referred to the web version of this article.)

Even accounting for differences in demography, contact patterns and health system capacity, vaccination targeting the oldest age groups is beneficial across all income settings. Under a strategy in which the elderly are targeted first, the required number of vaccine doses differs between income settings, reflecting the different age distributions (Figure S10). Fig. 4A–H shows the optimal allocation of the vaccine for each of the four income settings. In all settings, if doses are limited, the most efficient approach is to vaccinate the elderly population, starting at the oldest age group. In LIC settings, this approach broadly remains optimal. However, in HIC, UMIC and LMIC settings we find that above a given dose supply threshold and for a vaccine that reduces the risk of infection, the optimal allocation strategy switches from “direct protection of the vulnerable” to one of “herd impact”, whereby the vaccine is allocated to younger populations (including children and adults). Under such a scenario, community transmission is reduced and this indirectly reduces the risk to vulnerable older populations to a greater extent than through direct protection. Assuming that targeted age groups are vaccinated at 80% coverage, this switch occurs when there are sufficient doses to cover between 20% and 40% of the total population, although the threshold depends on both the relative size of the elderly population and on mixing patterns between the older population and the general population. Furthermore, in UMIC and LMIC settings we obtain a “mixed” approach at these intermediate dose availability levels, with both the highest risk elderly and younger populations included. This is due to greater mixing between the older and general populations in these settings, as well as the size of the high-risk older populations, which are generally smaller than in HIC settings. In all settings our optimisation includes the vaccination of children when this switch occurs.

Our model does not explicitly capture a lower transmission risk of children; if this is included, we find that they are de-prioritised (Figure S18). The most efficient strategy is also sensitive to vaccine efficacy (where for a lower efficacy, the level of vaccine supply at which a herd impact strategy becomes optimal is higher, Figure S12). For a vaccine that reduces the risk of severe disease only, the optimal strategy is to directly protect the vulnerable (Figure S14). If NPIs are maintained at a greater level following vaccine introduction, then we find that younger populations are targeted at a lower dose supply threshold (around 10%, Figure S15). Conversely, if transmission is higher, it is efficient to continue targeting older age groups even at a higher dose supply (Figure S16).

At a global level and assuming an initial vaccine dose supply of 2 billion, allocating doses equitably across all income settings relative to population size (Strategy B), or relative to population size in the 65 + age group (Strategy C), are the most efficient of the fixed approaches considered, assuming that within a country the vaccine is targeted to the at-risk population (Table 2). This finding is consistent across all sensitivity analyses considered (Table S6). If high-income countries can preferentially obtain a large proportion of the available vaccine doses at the expense of lower income countries then we would expect an additional 900 deaths per million from this less efficient global allocation, assuming that all countries have similar levels of pre-existing natural immunity (Table 2, Strategy D). Under a fully-optimised global allocation – in which allocation both within- and between- countries is optimised – we estimate the most efficient strategy can avert 1.43 deaths per 100 fully vaccinated persons, marginally higher than the equitable allocation with prioritisation to older age groups scenario (1.25 deaths averted per 100 fully vaccinated persons, Strategy C).

**Table 2:** **Global Allocation of Vaccine Doses for both Non-Optimised and Optimised Scenarios**. The global vaccine supply was assumed to be constrained to 2 billion doses, with a two-dose schedule and 15% buffer and wastage (resulting in 0.85 billion vaccine courses available). Table S4 shows impact for the same non-optimised allocation strategies, but with the assumption that limited countries within each income setting are allocated doses at high (80%) coverage. Table S6 shows the sensitivity analysis for each of the non-optimised scenarios. FVP: fully vaccinated persons.

Allocation strategy		Income setting	Target age group	Deaths averted per million	Deaths averted per 100 FVP	Total deaths averted per million global population	Total deaths averted per 100 FVP
Allocated to all countries at varying coverage	A: Countries receive doses in proportion to population	HIC	all	831	0.76	650	0.59
		UMIC	all	531	0.48		
		LMIC	all	730	0.66		
		LIC	all	482	0.44		
	B: Countries receive doses in proportion to population, with 65 + group prioritised and remaining doses allocated to 15–64 age groups	HIC	15+	1389	1.26	1324	1.20
		UMIC	15+	1270	1.14		
		LMIC	15+	1374	1.25		
		LIC	15+	1221	1.11		
	C: Countries receive doses in proportion to 65 + population, with 65 + group prioritised and remaining doses allocated to 15–64 age groups	HIC	15+	2238	0.9	1386	1.25
		UMIC	15+	1285	1.09		
		LMIC	15+	1237	1.91		
		LIC	15+	918	2.56		
	D: Allocated first to high-income countries	HIC	all	3234	0.46	513	0.46
		UMIC	all	0	0		
		LMIC	all	0	0		
		LIC	all	0	0		
	E: Allocated first to low-income and lower-middle-income countries	HIC	all	0	0	597	0.53
		UMIC	all	0	0		
		LMIC	all	1323	0.55		
		LIC	all	1074	0.45		
	F: Countries receive doses in proportion to population, plus 1.15b doses to HIC and 1.1b doses to MIC	HIC	all	3231	0.63	1308	0.56
		UMIC	all	898	0.47		
		LMIC	all	1099	0.58		
		LIC	all	482	0.44		
Optimised allocation	Allocation algorithm selects countries and age groups within targets to maximise deaths averted	HIC	optimised	3135	1.16	1672	1.43
		UMIC	optimised	1241	1.44		
		LMIC	optimised	1581	1.62		
		LIC	optimised	1533	1.81		

For a fully optimised global allocation, a higher proportion of doses is allocated to HICs (33% of global doses) with sufficient coverage (27%) in most HICs to pursue a herd impact strategy, assuming vaccination can be implemented while transmission remains suppressed (Table 3), relative to 28%, 33% and 6% of global doses in UMIC, LMIC and LIC settings, respectively. This is due primarily to the older populations in HIC but is also dependent on absolute population sizes in each income band. Under the optimal allocation scenario, in LIC, LMIC and UMIC settings the proportional coverage is lower (between 8 and 10% coverage) and hence doses are targeted to the elderly. Overall, assuming a vaccine reduces infection risk, the optimal allocation of 2 billion doses balances herd impact strategies in some countries with direct protection of the vulnerable in others.

**Table 3:** **Identified Optimal Global Allocation by Income Setting.** The optimal allocated coverage is the fully vaccinated persons per population, and the value in parentheses represents the proportion of total global doses allocated to that income setting. The Default scenario represents the default vaccine assumptions in Table 1. We also present the sensitivity of the allocation to changes in the assumptions about vaccine efficacy, vaccine efficacy in the 65 + years age group, mode of action of the vaccine, NPIs at vaccine introduction, health system constraints, reduced infectiousness in children younger than 10 years, and life-years gained (LYG) as an optimisation measure. The within-setting results are shown in Figures S12–S19, and the public health impact for each scenario is in Table S5. The estimated proportion of the global population in each income setting is included for reference.

	Allocated coverage of the population (proportion of global doses allocated to income setting)			
	**HIC**	**UMIC**	**LMIC**	**LIC**
Reference: Proportion of global population	15.9%	37.3%	38.1%	8.7%
Default scenario	27.1% (33.2%)	8.6% (27.9%)	9.8% (32.6%)	8.5% (6.2%)
Lower vaccine efficacy (70%)	32.7% (40.2%)	7.8% (25.3%)	8.5% (28.3%)	8.5% (6.2%)
Reduced vaccine efficacy (scaled by 50%) in 65 + years population	27.1% (33.2%)	11.5% (37.3%)	6.9% (23.2%)	8.5% (6.2%)
Vaccine efficacious against disease only	18.3% (22.5%)	7.8% (25.3%)	13.1% (43.7%)	11.6% (8.5%)
NPIs maintained at higher level following vaccine introduction (such that R_t2_ = 1.5)	11.3% (13.9%)	11.5% (37.1%)	11.2% (37.5%)	15.6% (11.5%)
NPIs maintained at lower level following vaccine introduction (such that R_t2_ = 2.5)	29.2% (35.8%)	7.8% (25.3%)	9.8% (32.6%)	8.5% (6.2%)
Health system constraints absent	27.1% (33.2%)	15% (48.7%)	4.5% (15.2%)	4% (3%)
Reduced infectiousness in children younger than 10 years	29.3% (35.9%)	7.8% (25.3%)	9.7% (32.5%)	8.5% (6.2%)
LYG as optimisation outcome measure	27.1% (33.2%)	1.7% (5.5%)	10.7% (35.8%)	34.7% (25.6%)

The global optimised strategy is sensitive to assumptions about the vaccine mode of action, with a more equitable allocation found to be optimal for a vaccine that is efficacious against disease only (Table 3). For a scenario with higher transmission following vaccine introduction relative to the default assumption, additional doses are allocated to HIC countries, however, for a scenario where NPIs are maintained to a greater extent following vaccination, a more equitable global allocation is optimal as the difference in averted deaths between income settings is less marked (Table 3, Figure S20). We also find that if life-years gained, rather than deaths averted, is used as the outcome measure, doses are diverted away from UMICs and allocated to LMIC and LIC settings, whereas removing assumptions about health system constraints causes doses to be allocated preferentially to HIC and UMIC countries (Table 3, Figures S17 and S19).

## 4 Discussion

Effective vaccines against SARS-CoV-2 will have enormous global public health and economic value. While the first vaccines are now being distributed, the dose supply in 2021 will be constrained. Current COVAX plans favour a global allocation strategy that prioritises the highest risk groups – including the elderly – and suggest an “equitable” vaccine allocation strategy in which each country receives doses in proportion to their population size and their epidemic status [7], [48]. Our results support such a strategy being close to optimal in terms of reducing the potential global mortality from SARS-CoV-2.

For most infectious disease vaccines, one of the underlying goals is to vaccinate a sufficiently high proportion of the population to achieve herd immunity. Our results demonstrate that such indirect protection remains important for SARS-CoV-2 despite the strong risk-profile with increasing age. Indirect protection is particularly important for vulnerable groups – such as the immunocompromised – who cannot be directly protected through vaccination. However, at a population level, the reduction in transmission also benefits those who receive the vaccine but do not achieve sterile protection. The coverage required to achieve herd immunity will depend on both the underlying transmissibility of the virus (R_0_) and vaccine efficacy (the effective coverage) and requires a vaccine that is efficacious against infection rather than only disease. For our assumed R_0_ of 2.5% and vaccine efficacy of 90%, population coverage of nearly 70% would be required. It is possible that heterogeneity in contact rates could reduce this theoretical coverage if vaccination is targeted to those with the highest contact rates [49], [50]. Furthermore, in the presence of ongoing NPIs, the effective coverage required to interrupt transmission will be lower. Nevertheless, aiming for high coverage will clearly be important given the uncertainty in the precise value of this threshold.

We focused on two metrics to capture the public health value of the vaccine: deaths averted, and life-years gained. These metrics were chosen because the IFR is the best characterised endpoint and is easily translatable between settings. However, the impact of SARS-CoV-2 in terms of longer-term morbidity – so-called “Long COVID” [51] – could also be considerable. If morbidity is significant then it could favour the prioritisation of vaccination towards younger age-groups. However, at the current time, the extent and longer-term consequences of COVID-19 remain relatively poorly understood, with no other comparable respiratory infections available with which to quantify QALYs. This will be a research priority in the coming months and years.

One of the most difficult challenges facing decision-makers in 2021 will be the allocation of a limited dose supply. Our results demonstrate that within a country, targeting a limited vaccine supply to older age groups is likely to be an efficient way to reduce mortality, particularly if doses are only available for less than approximately 20% of the population. If sufficient doses are available and if vaccination is efficacious against infection, targeting the working-age population, or a combination of older and working-age groups, could be more efficient (a finding consistent with other modelling studies [52], [53], [54], [55], [56]). However, the optimality of targeting younger groups may not hold if vaccines are delivered in a gradual phased approach, therefore if the early supply is constrained then targeting the most vulnerable will likely remain optimal. Such an approach also has the advantage that delivery systems are in place in many settings that can access such groups. Globally, under our baseline assumptions, a strategy where available vaccine doses are allocated preferentially to higher-income countries who have the largest elderly populations, but also have the strongest health systems, maximises the total averted deaths. However, the optimality of this allocation is sensitive to many assumptions and will depend on both the vaccine characteristics and on the stage of the epidemic in each country throughout vaccine introduction. Considering the optimisation framework applied, it is possible that there are other dose allocation strategies that would be close to optimal, but that distribute doses between countries in the same income setting differently. Given this uncertainty, allocating vaccine doses according to population size would be the next most efficient approach.

In our study we assessed the impact of vaccination for broad income strata, rather than individual countries. Decision-making at the country level will need to account for local epidemic history, levels of transmission, other control measures and how long they can be sustained, and the sizes of the populations at highest risk within those settings [57]. Some countries have identified prioritisation frameworks for allocating vaccines, with general agreement that frontline health care workers and high-risk populations should be allocated the first available doses, and our user-friendly COVID-19 vaccine simulation interface could help in supporting such decisions about country-level introductions (Box 1). However, there are many other setting-specific factors that will also be important for governments to consider, including supply chains, logistics, access to populations, costs and budgets, and competing health, economic, and social priorities [14].

As with any modelling study, there are several limitations. First, while phase III trial efficacy results have been announced for several vaccine candidates, durability data are not yet available, and data on the extent to which the current vaccine products can prevent infection are still emerging [2], [3], [29]; our model assumptions may therefore need to be updated as trials and effectiveness studies progress. Second, the number of doses and the timing of their availability are also uncertain; for this reason, we have illustrated simplified scenarios in which vaccination occurs over a one-month period for a range of epidemic stages. In practice, each country will have experienced a different epidemic when the first vaccine is introduced and will scale up coverage gradually. Third, the model used here is relatively simple in structure and can only simulate a single vaccine product, with one value for vaccine efficacy, meaning we could not include complexities such as multiple vaccine products, nor the partial efficacy following the first dose in a multi-dose vaccine schedule. These considerations will be important for future studies. Fourth, our study focuses only on the health benefits of vaccination. It will be important to consider other therapeutic interventions, as well as the capacity of countries to suppress transmission using NPIs, and to better capture specific risk groups as appropriate to individual countries. Furthermore, the direct health outcome is only one dimension; models that integrate epidemiological and economic outcomes will be needed to evaluate the impact of different vaccine allocation strategies on the economic outputs of countries and the livelihoods of their citizens.

SARS-CoV-2 vaccine research and development has taken place at unprecedented speed, with efficacy and safety results now available for several vaccine candidates within one year of the pandemic being declared. Our results demonstrate that the global public health value of the vaccine can be maximised by ensuring equitable access. Acting collectively in this way during the early stages of vaccine deployment remains the ethical approach to take, even if this is not the most beneficial short-term strategy from a national perspective.

## Funding

PW and ABH acknowledge fellowship funding from Imperial College London. PGTW, OJW, ACG and NMF acknowledge grant funding from The Wellcome Trust and the UK Foreign, Commonwealth & Development Office (FCDO) (reference 221350/Z/20/Z). CW acknowledges support through a Medical Research Council (MRC) Doctoral Training Programme studentship. NB, GC and ACG acknowledge support from the Bill and Melinda Gates Foundation. All authors acknowledge funding support for the MRC Centre for Global Infectious Disease Analysis (reference MR/R015600/1), jointly funded by the UK MRC and the UK FCDO, under the MRC/FCDO Concordat agreement, also part of the EDCTP2 programme supported by the European Union. NMF and KH acknowledge funding by Community Jameel. NMF acknowledges support from the NIHR HPRU in Modelling and Health Economics, a partnership between PHE, Imperial College London and LSHTM (grant code NIHR200908). DJL and NMF acknowledge funding from Vaccine Efficacy Evaluation for Priority Emerging Diseases (VEEPED) grant (reference NIHR: PR-OD-1017-20002) from the National Institute for Health Research. ACG, ABH, MB, and PW received personal fees from the World Health Organization in relation to developing the online interface (approximately GBP 1,000 per individual), and the WHO provided input into the design of and data underpinning the online interface. The funders had no other role in study design, conduct, or interpretation of results. For the purpose of open access, the author has applied a CC BY public copyright licence to any Author Accepted Manuscript version arising from this submission. *Disclaimer: The views expressed are those of the authors and not necessarily those of the United Kingdom (UK) Department of Health and Social Care, the National Health Service, the National Institute for Health Research (NIHR), or Public Health England (PHE)*.

## Author contributions

ABH, PW and ACG designed the study with input from FM, KAMG, KH, MB, and NMF. ABH, PW, GC and OJW coded the model, ran the simulations and undertook the analysis with support from CW, NB, PGTW, and RV. AH, EK, JAL, and LW provided additional technical assistance with the model development and parameterisation. ABH, PW and ACG produced the first draft of the manuscript with additional input from AL, CW, DJL, KAMG, FM, KH, MB, NMF, OJW, and PGTW. All authors approved the final draft.

## 7 Data availability

The open-source model code is available as an R package at https://github.com/mrc-ide/nimue, and the code to perform the analysis and generate the tables and figures, is available to download at https://github.com/mrc-ide/covid_vaccine_allocation.

## Declaration of Competing Interest

The authors declare grants from The Wellcome Trust (NMF, ACG), UK Medical Research Council (NMF, ACG, KH), National Institute for Health Research (NMF, KH), Community Jameel (NMF, KH), the UK Foreign, Commonwealth and Development Office (OJW) and the Bill and Melinda Gates Foundation (NMF), during the conduct of the study; grants from the Bill and Melinda Gates Foundation (ACG), National Institute for Health (ACG), GlaxoSmithKline (AL), and Gavi, the Vaccine Alliance (ACG, KAMG) outside the submitted work; personal fees from the World Health Organization (ACG, ABH, MB, PW) during the conduct of the study, in relation to developing the online interface (approximately £1000 per individual); and personal fees from The Global Fund (ACG, PW) outside the submitted work. ABH was previously engaged by Pfizer Inc to advise on modelling RSV vaccination strategies for which she received no financial compensation. There are no other relationships or activities that could appear to have influenced the submitted work.

## References

[1] Craven J. COVID-19 vaccine tracker. Regul. Aff. Prof. Soc. 2021. https://www.raps.org/news-and-articles/news-articles/2020/3/covid-19-vaccine-tracker (accessed Jan 26, 2021).

[2] Voysey M., Clemens S.A.C., Madhi S.A. (2020). Safety and efficacy of the ChAdOx1 nCoV-19 vaccine (AZD1222) against SARS-CoV-2: an interim analysis of four randomised controlled trials in Brazil, South Africa, and the UK. Lancet.

[3] Voysey M., Costa Clemens S.A., Madhi S.A. (2021). Single-dose administration and the influence of the timing of the booster dose on immunogenicity and efficacy of ChAdOx1 nCoV-19 (AZD1222) vaccine: a pooled analysis of four randomised trials. Lancet.

[4] Baden L.R., El Sahly H.M., Essink B. (2020). Efficacy and Safety of the mRNA-1273 SARS-CoV-2 Vaccine. N Engl J Med.

[5] Polack F.P., Thomas S.J., Kitchin N. (2020). Safety and Efficacy of the BNT162b2 mRNA Covid-19 Vaccine. N Engl J Med.

[6] Logunov D.Y., Dolzhikova I.V., Shcheblyakov D.V. (2021). Safety and efficacy of an rAd26 and rAd5 vector-based heterologous prime-boost COVID-19 vaccine: an interim analysis of a randomised controlled phase 3 trial in Russia. Lancet.

[7] GAVI. COVAX: Ensuring global equitable access to COVID-19 vaccines. https://www.gavi.org/covid19/covax-facility (accessed Aug 4, 2020).

[8] BBC. Coronavirus: US buys nearly all of Gilead’s Covid-19 drug remedesivir. 2020. https://www.bbc.co.uk/news/world-us-canada-53254487 (accessed Aug 5, 2020).

[9] Stone J. Government says medicines must be stock-piled for no-deal Brexit on top of Covid-19 pressures. Independent. 3 Aug 2020. https://www.independent.co.uk/news/uk/politics/coronavirus-no-deal-brexit-government-medicine-stockpiling-a9651991.html (accessed Sept 17, 2020).

[10] World Health Organization. Ethics and COVID-19: resource allocation and priority-setting. 2020. https://www.who.int/ethics/publications/ethics-and-covid-19-resource-allocation-and-priority-setting/en/ (accessed Jan 26, 2021).

[11] Persad G., Wertheimer A., Emanuel E. (2009). Principles for allocation of scarce medical interventions. Lancet.

[12] Emanuel E.J., Persad G., Upshur R. (2020). Fair allocation of scarce medical resources in the time of covid-19. N Engl J Med.

[13] Verity R., Okell L.C., Dorigatti I. (2020). Estimates of the severity of coronavirus disease 2019: a model-based analysis. Lancet Infect Dis.

[14] Russell F.M., Greenwood B. (2020). Who should be prioritised for COVID-19 vaccination?. Hum Vaccines Immunother.

[15] Walker P.G.T., Whittaker C., Watson O.J. (2020). The impact of COVID-19 and strategies for mitigation and suppression in low- and middle-income countries. Science.

[16] The World Bank. DataBank World Development Indicators. 2020. https://databank.worldbank.org/ (accessed Sept 1, 2020).

[17] United Nations. United Nations World Population Prospects. 2020. https://population.un.org/wpp/ (accessed July 1, 2020).

[18] Pritchard M, Dankwa EA, Hall M (2020). ISARIC Clinical Data Report 4 October 2020. medRxiv.

[19] Intensive Care National Audit & Research Centre. ICNARC report on COVID-19 in critical care: 16 October 2020. 2020. https://www.icnarc.org/Our-Audit/Audits/Cmp/Reports (accessed Jan 31, 2021).

[20] Salje H., Kiem C.T., Lefrancq N. (2020). Estimating the burden of SARS-CoV-2 in France. Science.

[21] Brazeau NF, Verity R, Jenks S, et al. Report 34 - COVID-19 Infection Fatality Ratio estimates from seroprevalence. 2020. doi:10.25561/83545.

[22] Dong E., Du H., Gardner L. (2020). An interactive web-based dashboard to track COVID-19 in real time. Lancet Infect Dis.

[23] Worldometer. Coronavirus updates. https://www.worldometers.info/ (accessed Jan 26, 2021).

[24] MRC Centre for Global Infectious Disease Analysis Imperial College London. Future scenarios of the healthcare burden of COVID-19 in Low- or Middle-income countries. 2021. https://mrc-ide.github.io/global-lmic-reports/ (accessed Jan 26, 2021).

[25] Public Health England. PHE monitoring of the early impact and effectiveness of COVID-19 vaccination in England, 22 February 2021. https://www.gov.uk/government/publications/phe-monitoring-of-the-effectiveness-of-covid-19-vaccination (accessed 31 March 2021).

[26] Bernal JL, Andrews N, Gower C (2021). Early effectiveness of COVID-19 vaccination with BNT162b2 mRNA vaccine and ChAdOx1 adenovirus vector vaccine on symptomatic disease, hospitalisations and mortality in older adults in England. medRxiv.

[27] Amit S., Regev-Yochay G., Afek A., Kreiss Y., Leshem E. (2021). Early rate reductions of SARS-CoV-2 infection and COVID-19 in BNT162b2 vaccine recipients. Lancet.

[28] Hall V, Foulkes S, Saei A, et al. Effectiveness of BNT162b2 mRNA Vaccine Against Infection and COVID-19 Vaccine Coverage in Healthcare Workers in England, Multicentre Prospective Cohort Study (the SIREN Study). SSRN Prepr 2021. https://papers.ssrn.com/sol3/papers.cfm?abstract_id=3790399. 10.1016/S0140-6736(21)00790-X PMC8064668 33901423

[29] Weekes M., Jones N.K., Rivett L. (2021). Single-dose BNT162b2 vaccine protects against asymptomatic SARS-CoV-2 infection. eLife.

[30] Jackson L.A., Anderson E.J., Rouphael N.G., Roberts P.C., Makhene M., Coler R.N. (2020). Beigel for the mRNA-1273 SG. An mRNA Vaccine against SARS-CoV-2 — Preliminary Report. N Engl J Med.

[31] Folegatti P.M., Ewer K.J., Aley P.K. (2020). Safety and immunogenicity of the ChAdOx1 nCoV-19 vaccine against SARS-CoV-2: a preliminary report of a phase 1/2, single-blind, randomised controlled trial. Lancet.

[32] Zhu F.C., Li Y.H., Guan X.H. (2020). Safety, tolerability, and immunogenicity of a recombinant adenovirus type-5 vectored COVID-19 vaccine: a dose-escalation, open-label, non-randomised, first-in-human trial. Lancet.

[33] Mulligan M.J., Lyke K.E., Kitchin N. (2020). Phase I/II study of COVID-19 RNA vaccine BNT162b1 in adults. Nature.

[34] Sahin U., Muik A., Derhovanessian E. (2020). COVID-19 vaccine BNT162b1 elicits human antibody and TH1 T cell responses. Nature.

[35] Ministerio de Ciencia e Innovaciaon Gobierno de Espana, Ministerio de Sanidad Gobierno de Espana, Consejo Interterritorial, Instituto de Salud Carlos III. Estudio Nacional de Sero-Epidemiolgia de la Infeccion por SARS-CoV-2 en Espana: Informe Final. 2020 https://www.mscbs.gob.es/ciudadanos/ene-covid/.

[36] To K.K.W., Hung I.F., Ip J.D. (2020). COVID-19 re-infection by a phylogenetically distinct SARS-coronavirus-2 strain confirmed by whole genome sequencing. Clin Infect Dis.

[37] Hall V, Foulkes S, Charlett A (2021). Do antibody positive healthcare workers have lower SARS-CoV-2 infection rates than antibody negative healthcare workers? Large multi-centre prospective cohort study (the SIREN study), England: June to November 2020. medRxiv.

[38] World Health Organization. WHO Target Product Profiles for COVID-19 Vaccines. Version 3. Geneva, 2020 https://www.who.int/publications/m/item/who-target-product-profiles-for-covid-19-vaccines.

[39] Naghavi M., Abajobir A.A., Abbafati C. (2017). Global, regional, and national age-sex specifc mortality for 264 causes of death, 1980–2016: A systematic analysis for the Global Burden of Disease Study 2016. Lancet.

[40] Emanuel E.J., Persad G., Kern A. (2020). An ethical framework for global vaccine allocation. Science.

[41] World Health Organization. 172 countries and multiple candidate vaccines engaged in COVID-19 vaccine Global Access Facility. 2020. https://www.who.int/news/item/24-08-2020-172-countries-and-multiple-candidate-vaccines-engaged-in-covid-19-vaccine-global-access-facility (accessed Jan 27, 2021).

[42] World Health Organization. Revising global indicative wastage rates: a WHO initiative for better planning and forecasting of vaccine supply needs. Concept Note. 8 April 2019. 2019 ; 1–6.

[43] Schumacher D. ompr: Model and Solve Mixed Integer Linear Programs. R package version 0.8.0. 2020. https://cran.r-project.org/package=ompr.

[44] Haq K., McElhaney J.E. (2014). Immunosenescence: Influenza vaccination and the elderly. Curr Opin Immunol.

[45] Arinaminpathy N., Kim I.K., Gargiullo P. (2017). Estimating Direct and Indirect Protective Effect of Influenza Vaccination in the United States. Am J Epidemiol.

[46] Davies NG, Barnard RC, Jarvis CI (2020). Estimated transmissibility and severity of novel SARS-CoV-2 Variant of Concern 202012/01 in England. medRxiv.

[47] Volz E, Mishra S, Chand M (2021). Transmission of SARS-CoV-2 Lineage B.1.1.7 in England: Insights from linking epidemiological and genetic data. medRxiv.

[48] Gavi. 92 low- and middle-income economies eligible to get access to COVID-19 vaccines through Gavi COVAX AMC. 2020. https://www.gavi.org/news/media-room/92-low-middle-income-economies-eligible-access-covid-19-vaccines-gavi-covax-amc (accessed Sept 17, 2020).

[49] Britton T., Ball F., Trapman P. (2020). A mathematical model reveals the influence of population heterogeneity on herd immunity to SARS-CoV-2.. Science.

[50] Fontanet A., Cauchemez S. (2020). COVID-19 herd immunity: where are we?. Nat Rev Immunol.

[51] Mahase E. (2020). Covid-19: What do we know about ‘long covid’?. BMJ.

[52] Matrajt L., Eaton J., Leung T., Brown E.R. (2021). Vaccine optimization for COVID-19, who to vaccinate first?. Science.

[53] Babus A, Das S, Lee S (2020). The Optimal Allocation of Covid-19 Vaccines. medRxiv.

[54] Chen X, Li M, Simchi-Levi D, Zhao T (2020). Allocation of COVID-19 Vaccines Under Limited Supply. medRxiv.

[55] Bubar K.M., Reinholt K., Kissler S.M. (2021). Model-informed COVID-19 vaccine prioritization strategies by age and serostatus. Science.

[56] Jentsch P., Anand M., Bauch C.T. (2021). Prioritising COVID-19 vaccination in changing social and epidemiological landscapes. Lancet Infect Dis.

[57] Clark A., Jit M., Warren-Gash C. (2020). Global, regional, and national estimates of the population at increased risk of severe COVID-19 due to underlying health conditions in 2020: a modelling study. Lancet Glob Heal.

[58] Institute of Global Health Innovation, YouGov. Covid-19: Global attitudes towards a COVID-19 vaccine. 2020. https://www.imperial.ac.uk/media/imperial-college/institute-of-global-health-innovation/GlobalVaccineInsights_ICL-YouGov-Covid-19-Behaviour-Tracker_20201202_v6.pdf (accessed Jan 26, 2021).

[59] Hansen C.H., Michlmayr D., Gubbels S.M., Mølbak K., Ethelberg S. (2021). Articles Assessment of protection against reinfection with SARS-CoV-2 among 4 million PCR-tested individuals in Denmark in 2020: a population-level observational study. Lancet.

[60] Dan J.M., Mateus J., Kato Y. (2021). Immunological memory to SARS-CoV-2 assessed for up to 8 months after infection. Science.

[61] World Health Organization. National Health Workforce Accounts (NHWA). 2021. https://www.who.int/hrh/statistics/nhwa/en/ (accessed Jan 26, 2021).

