# Supplementary material for: Within-country age-based prioritisation, global allocation, and public health impact of a vaccine against SARS-CoV-2: A mathematical modelling analysis
Source: Vaccine. 2021 May 21;39(22):2995–3006. doi: 10.1016/j.vaccine.2021.04.002 (PMC8030738; doi:10.1016/j.vaccine.2021.04.002)
Supplement: Supplementary data 1 [file mmc1.pdf]

Supplementary Information

Within-country age-based prioritisation, global allocation, and public health impact of a vaccine against SARS-CoV-2: a mathematical modelling analysis

1 Mathematical model

1.1 Description

We extended an age-structured deterministic SEIR compartmental model of SARS-CoV-2 transmission to capture the impact of different vaccination schedules. The main model captures the infection status of individuals as being in one of nine states (Figure S1):

- S = uninfected and therefore **susceptible** to infection
- E = **exposed** to infection but not yet infectious
- I_{MILD} = **infected** and infectious with mild infection that does not require hospitalisation (this includes both symptomatic and asymptomatic infection)
- I_{CASE} = **infected** and infectious with disease that will require hospitalisation
- I_{HOSP} = cases that have been **hospitalised** in a general ward bed
- I_{ICU} = cases that have been admitted to **an intensive care unit (ICU)**
- I_{REC} = cases that have been stepped down from ICU into a general ward bed for **recovery**
- D = cases that have **died**
- R = infections and cases that have **recovered** and are immune to re-infection

Those that are susceptible become infected at a rate that depends on the level of infection in the community (i.e. the number of people in states I_{MILD} and I_{CASE}), the transmission probability and an age-stratified mixing matrix which captures the relative number of contacts that each age group has with their own age group and other age groups. This mixing matrix, along with the demography of the population, is obtained from studies that are representative of each of the four different World Bank income groups. Given the other model parameters, the transmission probability is varied to generate a different basic reproductive number, R_0. Following infection, cases proceed as shown by the arrows with the durations in each state and the probability of each pathway described below (see Tables S1 and S2). Following recovery, we allow individuals to lose naturally-acquired immunity and therefore return to the susceptible state. Additional constraints are included in the hospitalisation pathway to capture situations in which the need exceeds capacity; with those that do not receive appropriate care experiencing higher death rates.

Individuals who are susceptible, in the latent period, or recovered can be vaccinated. We assume that people who are currently infected do not receive the vaccine, and while this simplification may miss asymptomatic individuals, such people represent a small fraction of the total population at any given time. The process of vaccination is captured by mirroring the basic transmission model to also capture different states of vaccination (Figure S1):

- v_0 – unvaccinated
- v_1 and v_2 – vaccinated but not yet protected, reflecting the two-dose vaccine schedule and need to wait approximately 28 days from dose 1 for protection to develop
- v_3 and v_4 – vaccinated and protected
- v_5 – previously vaccinated but no longer protected, used to capture waning vaccine efficacy.

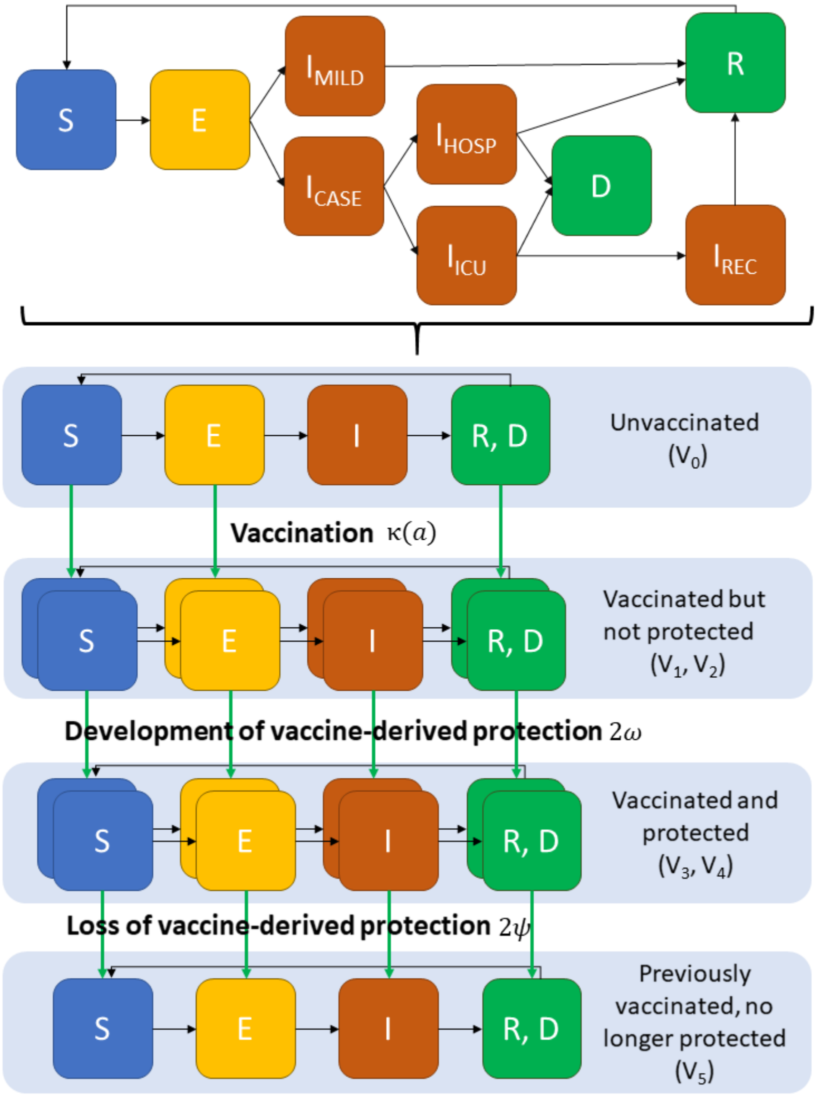

Figure S1: Schematic of the SARS-CoV-2 transmission model. *The schematic in the upper section is based on the model in Walker et al.*[1] *Individuals in the susceptible (S), exposed (E) and recovered (R) compartments can be vaccinated. Vaccination status is stratified into 6 categories – those who are unvaccinated (v_0), those who have recently been vaccinated but are not yet protected (v_1 and v_2) and those who are vaccinated and protected (v_3 and v_4) and those that have previously been vaccinated but are no longer protected (v_5). Protection may refer to partial protection. We do not model revaccination of previously vaccinated individuals, due to all vaccination occurring within a one-month period. Pathways for receiving healthcare for the proportions of the population in the $I_{HOSPITAL}$ and I_{ICU} compartments are described in Walker et al.*[1]

If the vaccine is not 100% efficacious, then in the protected states we assume there is partial efficacy. For example, if the vaccine is 90% efficacious then the risk of infection in states v_3 and v_4 is 90% lower than in state v_0, v_1, v_2, and v_5. We assume that vaccine efficacy is the same for susceptible and immune individuals. For those protected by both vaccine-derived and naturally-acquired immunity, we assume that the most protective effect is dominant. For example, if natural immunity lasts for one year and vaccine-derived immunity for five years then the period of protection for a recently infected and now immune individual who receives the vaccine would be one year of full naturally-acquired protection, followed by four years of vaccine-derived (leaky) protection.

1.2 Formal mathematical details

We stratify the population by age, a, and vaccination status, v. For the latter, we incorporate six categories – those who are unvaccinated (v_0), those who have recently been vaccinated but are not yet protected (v_1 and v_2) and those who are vaccinated and protected (v_3 and v_4) and those who have previously been vaccinated but are no longer protected (v_5). Note that we use the term "protected" here to denote partial protection because we model a leaky vaccine rather than sterile immunity. Protection is provided either against infection – reducing the transmission parameter β by a constant factor $1 - \upsilon_{inf}(a)$; against severe disease – reducing the rate of hospitalisation $\phi(a)$ by a constant factor $1 - \upsilon_{dis}(a)$; or against infection, with additional protection against severe disease in vaccinated individuals who experience breakthrough infection (combined modes of efficacy).

Vaccines are targeted by age groups at a rate $\kappa(a)$. To specify prioritisation strategies, we define a vector of current vaccine coverage C_a by age group (a) and a matrix of coverage targets T_{sa} where rows (s) represent ordered prioritisation steps and columns (a) the age group. For a given prioritisation step, e.g. $s = 1$, vaccination, up to a maximum specified rate expressed as the total number of vaccinees per day, is provided to all age groups that satisfy $C_a < T_{1a}$. Vaccinations are distributed to target age groups in proportion to the size of the unvaccinated populations. When all target coverages in the current prioritisation step are met, the step (s) is incremented $(+1)$ and the process repeated. When all coverage targets in the final prioritisation step are met, vaccination is ceased. We assume that individuals may only be vaccinated once.

The transmission model within each vaccine category is identical to that described in Walker *et al.*[1] with the inclusion of reversion of natural immunity which is modelled by an additional flow from the recovered compartment back to the susceptible compartment (Figure S1). Let *S(t,a,v)* denote the susceptible population in age group a with vaccination status v at time t , $E_1(t,a,v)$ and $E_2(t,a,v)$ two sequential latent periods, $I_{MILD}(t,a,v)$ infections that are either asymptomatic or symptomatic but do not require hospitalisation, $I_{CASE,0}(t,a,v)$ and $I_{CASE,1}(t,a,v)$ two sequential states for infections that are symptomatic and will subsequently require hospitalisation. $I_{HOSPITAl,,0}(t,a,v)$ and $I_{HOSPITAL,1}(t,a,v)$ are two sequential states for infections requiring a general hospital bed. $I_{ICU,0}(t,a,v)$ and $I_{ICU,1}(t,a,v)$ are two sequential states for infections requiring an ICU bed. $I_{REC,0}(t,a,v)$ and $I_{REC,1}(t,a,v)$ are two sequential states for hospitalised infections in general beds recovering from ICU whilst $R_1(t,a,v)$ and $R_2(t,a,v)$ are two sequential compartments capturing those that have recovered and are currently immune to re-infection, and *D(t,a,v,)* are those that have died from the disease in age group a and with vaccination status v. We further split $I_{HOSPITAL,i}(t,a,v)$ and $I_{ICU,i}(t,a,v)$ into states i=1,2 to track those that either receive or do not receive their hospital or ICU bed respectively (1, 0 respectively depending on capacity constraints) and through these route either die or recover (0 and 1 respectively) in order to capture different durations of stay in hospital dependent on outcome. For example, the state tracking those

that require a general bed, receive it and go on to die is $I_{HOSPITAL,i}(t,a,v,1,0)$ whilst the state tracking those that require a general bed, do not receive it and go on to die is $I_{HOSPITAL,i}(t,a,v,0,0)$. In the equations below we use the Kronecker Delta function $\delta(\cdot)$ to capture capacity constraints with this equal to 1 if there is capacity (Hospital or ICU) and zero otherwise. Contacts between age-classes are captured using the social contact mixing matrix where $c(a, a')$ denotes the rate of contacts between individuals in age groups a and a'. Age-dependent severity of disease is captured with an age-dependent mortality rate $\mu(a)$. Note that hospitalised individuals and those in ICU do not contribute to the force of infection.

A mean duration in ICU of 13.3 days is reported from a study of clinical outcomes of COVID-19 hospitalised patients across 42 countries[2]. In UK data, 60.1% of those entering ICU survive with the ratio of the duration of time spent in ICU in those that die to those who survive reported as 0.75[3]. We use these two additional pieces of information to obtain the mean duration of stay in ICU for individuals who die and those who survive.

The model incorporates three age-dependent parameters – the probability of hospitalisation given infection $\phi_1(a)$; the probability of requiring ICU given hospitalisation $\phi_2(a)$; and the probability of dying $\mu(a)$ given hospitalisation (when hospital capacity is not saturated, we make the simplifying assumption that all deaths occur in hospital) (Table S2). The probability of dying given hospitalisation is obtained from estimates of the infection fatality ratio from a recent systematic review and model-based analysis of the literature[4]. Deaths in hospital occur from both those entering ICU and those in general beds, with the proportion in each determined by an age-dependent parameter (Table S2). The shape of this distribution reflects a lower proportion of elderly patients being admitted to ICU and is informed by unpublished UK data. For a UK demography, this parameterisation results in 20% of all hospitalised cases requiring ICU (consistent with the ISARIC international study reporting 18%[2]), 26% of hospitalised patients dying (slightly lower the ISARIC international study reporting 32%[2]), a case fatality ratio in ICU of 48% (compared with the ISARIC international study reporting 54%[2]) and 41% of deaths occurring in ICU (slightly higher than the ISARIC international study reporting 36%[2]).

The IFR estimates presented in Brazeau *et al.*[4] also include a breakdown in five-year age bands for those above 80 years of age. To obtain the correct IFR in the 80+ age group we weighted the IFR reported by 5-year age bands by the proportion of the 80+ population in each 5-year band for each income setting to obtain an average IFR in the 80+ age group.

Given that our focus is on short-term dynamics we do not model births, deaths, or aging. The group of individuals therefore represents their age in 2020. The equations for each of the six vaccination groups are given in Section 1.3. The parameter symbols, description and values are shown in Tables S1, S2, and S3.

Table S1: Parameter descriptions and values.

Parameter	Symbol	Value	Description
**Epidemiological Parameters**			
Transmission parameter	β	-	Calculated from R_0 (See Table 1)
Mean latent period	$\frac{1}{\alpha}$	4.6 days	Estimated at 5.1 days[5–7]. The last 0.5 days are incorporated in the infectious periods to capture pre-symptomatic infectivity
Mean duration of mild infection	$\frac{1}{\gamma_1}$	2.1 days	Incorporates 0.5 days of infectiousness prior to symptoms. In combination with mean duration of severe illness this gives a mean serial interval of 6.75 days[8].
Mean duration of severe infection prior to hospitalisation	$\frac{1}{\gamma_2}$	4.5 days	Mean onset-to-admission of 4 days[9]. Values in the wider literature range from 1.2 days to 12 days[5–7,10,11]. Includes 0.5 days of infectiousness prior to symptom onset.
Mean duration of hospitalisation for non-critical cases if survive	$\frac{1}{\gamma_{3,1}}$	9 days	Median of values identified in[10–14].
Mean duration of hospitalisation for non-critical cases if die	$\frac{1}{\gamma_{3,0}}$	9 days	Median of values identified in[10–14].
Mean duration in ICU if survive	$\frac{1}{\gamma_{4,1}}$	14.8 days	Mean duration in ICU of 13.3 days from a study across 42 countries[2]. Ratio of duration in critical care if die: duration in critical care if survive of 0.75 and 60.1% probability of survival in ICU[3].
Mean duration in ICU if die	$\frac{1}{\gamma_{4,0}}$	11.1 days	Mean duration in ICU of 13.3 days from a study across 42 countries[2]. Ratio of duration in critical care if die: duration in critical care if survive of 0.75 and 60.1% probability of survival in ICU[3].
Mean duration in recovery after ICU	$\frac{1}{\gamma_5}$	3.0 days	Working assumption
Infection fatality ratio (IFR)	$\mu(a)$	-	Age-dependent[4] (see Table S2)
Hospitalisation proportion	$\phi_1(a)$	-	Age-dependent[15] (see Table S2)
Proportion of hospitalisations requiring ICU	$\phi_2(a)$	-	Age-dependent[15] (see Table S2)
Mean duration of naturally acquired immunity	$1/\rho$	365 days (default), infinite, or 183 days	[16–20]
**Vaccination Parameters**			
Age-dependent rate of vaccination	$\kappa(a)$	-	Age-dependent assumption: set such that number of people vaccinated per day in each age group achieves target coverage by the end of the vaccination period
Mean time to develop vaccine-acquired immunity following second dose	$1/\omega$	7 days	[21–25]
Mean duration of vaccine-acquired immunity	$1/\psi$	5000 days (default), 365 days or 183 days	[26]
Efficacy against infection	$1 - \upsilon_{inf}(a)$	-	Age-dependent (see Table S3)[27–32]
Efficacy against disease	$1 - \upsilon_{dis}(a)$	-	Age-dependent (see Table S3)[28,30,33–36]
Dose schedule	-	2 doses (default); 1 dose	[27,30–32]

Table S2: Age-dependent parameters for hospitalisation and death.

Age group (years)	Proportion of infections hospitalised[15]	Proportion of hospitalised cases requiring ICU[15]	Proportion of hospital deaths occurring in ICU	Proportion of non-ICU cases dying	Proportion of ICU cases dying	Infection fatality ratio (IFR)[4]
0 to 4	0.001	0.181	0.8	0.013	0.227	0.00004
5 to 9	0.001	0.181	0.8	0.014	0.252	0.00007
10 to 14	0.002	0.181	0.8	0.016	0.281	0.00011
15 to 19	0.002	0.137	0.8	0.016	0.413	0.00017
20 to 24	0.003	0.122	0.8	0.018	0.518	0.00026
25 to 29	0.005	0.123	0.8	0.020	0.573	0.00041
30 to 34	0.007	0.136	0.8	0.023	0.576	0.00064
35 to 39	0.009	0.161	0.8	0.026	0.543	0.00100
40 to 44	0.013	0.197	0.8	0.030	0.494	0.00156
45 to 49	0.018	0.242	0.8	0.036	0.447	0.00245
50 to 54	0.025	0.289	0.8	0.042	0.417	0.00384
55 to 59	0.036	0.327	0.8	0.050	0.411	0.00601
60 to 64	0.050	0.337	0.8	0.056	0.443	0.00941
65 to 69	0.071	0.309	0.8	0.060	0.539	0.01473
70 to 74	0.100	0.244	0.6	0.123	0.570	0.02307
75 to 79	0.140	0.160	0.4	0.184	0.643	0.03613
80+	0.233	0.057	0.15	0.341	0.993	-*
80 to 84	-	-	-	-	-	0.05659
85 to 89	-	-	-	-	-	0.08862
90+	-	-	-	-	-	0.17370

* To standardise input age groups for modelling, IFRs in 80 to 84, 85 to 89 and 90+ years age groups are aggregated to the 80+ years age group using country-specific demography.

Table S3: Scenarios for modes of vaccine efficacy.

**Mode of efficacy scenario**	**Efficacy against infection**	**Efficacy against disease**
Combined (default)	90%	Additional 60% efficacy for breakthrough infections
Infection only	90%	No additional protection
Disease only	0%	90%
Combined, with immunosenescence	90%; 45% in 65+ year age group	Additional 60% efficacy for breakthrough infections
Combined, lower efficacy	70%	Additional 60% efficacy for breakthrough infections

1.3 Mathematical model equations

1.3.1 Vaccination group v_0 - unvaccinated

$$\frac{dS(t,a,v_0)}{dt} = 2\rho R_2(t,a,v_0) - \beta \frac{S(t,a,v_0)}{N} \sum_{a'} c(a,a')[\sum_{v} (I_{MILD}(t,a',v) + I_{CASE}(t,a',v))] - \kappa(a)S(t,a,v_0)$$

$$\frac{dE_1(t,a,v_0)}{dt} = \beta \frac{S(t,a,v_0)}{N} \sum_{a'} c(a,a')[\sum_{v} (I_{MILD}(t,a',v) + I_{CASE}(t,a',v))] - 2\alpha E_1(t,a,v_0) - \kappa(a)E_1(t,a,v_0)$$

$$\frac{dE_2(t,a,v_0)}{dt} = 2\alpha E_1(t,a,v_0) - 2\alpha E_2(t,a,v_0) - \kappa(a)E_2(t,a,v_0)$$

$$\frac{dI_{MILD}(t,a,v_0)}{dt} = (1-\phi_1(a))(2\alpha E_2(t,a,v_0)) - \gamma_1 I_{MILD}(t,a,v_0)$$

$$\frac{dI_{CASE,0}(t,a,v_0)}{dt} = \phi_1(a)(2\alpha E_2(t,a,v_0)) - 2\gamma_2 I_{CASE,0}(t,a,v_0)$$

$$\frac{dI_{CASE,1}(t,a,v_0)}{dt} = 2\gamma_2 I_{CASE,0}(t,a,v_0) - 2\gamma_2 I_{CASE,1}(t,a,v_0)$$

$$\frac{dI_{HOSPITAL,0}(t,a,v_0,0{,}0)}{dt} = (1-\delta(H))\mu(a)(1-\phi_2(a))2\gamma_2 I_{CASE,1}(t,a,v_0) - 2\gamma_{3,0} I_{HOSPITAL,0}(t,a,v_0,0{,}0)$$

$$\frac{dI_{HOSPITAL,1}(t,a,v_0,0{,}0)}{dt} = 2\gamma_{3,0} I_{HOSPITAL,0}(t,a,v_0,0{,}0) - 2\gamma_{3,0} I_{HOSPITAL,1}(t,a,v_0,0{,}0)$$

$$\frac{dI_{HOSPITAL,0}(t,a,v_0,1{,}0)}{dt} = \delta(H)\mu(a)(1-\phi_2(a))2\gamma_2 I_{CASE,1}(t,a,v_0) - 2\gamma_{3,0} I_{HOSPITAL,0}(t,a,v_0,1{,}0)$$

$$\frac{dI_{HOSPITAL,1}(t,a,v_0,1{,}0)}{dt} = 2\gamma_{3,0} I_{HOSPITAL,0}(t,a,v_0,1{,}0) - 2\gamma_{3,0} I_{HOSPITAL,1}(t,a,v_0,1{,}0)$$

$$\frac{dI_{HOSPITAL,0}(t,a,v_0,0{,}1)}{dt} = (1-\delta(H))(1-\mu(a))(1-\phi_2(a))2\gamma_2 I_{CASE,1}(t,a,v_0) - 2\gamma_{3,1} I_{HOSPITAL,0}(t,a,v_0,0{,}1)$$

$$\frac{dI_{HOSPITAL,1}(t,a,v_0,0{,}1)}{dt} = 2\gamma_{3,1} I_{HOSPITAL,0}(t,a,v_0,0{,}1) - 2\gamma_{3,1} I_{HOSPITAL,1}(t,a,v_0,0{,}1)$$

$$\frac{dI_{HOSPITAL,0}(t,a,v_0,1{,}1)}{dt} = \delta(H)(1-\mu(a))(1-\phi_2(a))2\gamma_2 I_{CASE,1}(t,a,v_0) - 2\gamma_{3,1} I_{HOSPITAL,0}(t,a,v_0,1{,}1)$$

$$\frac{dI_{HOSPITAL,1}(t,a,v_0,1{,}1)}{dt} = 2\gamma_{3,1} I_{HOSPITAL,0}(t,a,v_0,1{,}1) - 2\gamma_{3,1} I_{HOSPITAL,1}(t,a,v_0,1{,}1)$$

$$\frac{dI_{ICU,0}(t,a,v_0,0{,}0)}{dt} = (1-\delta(ICU))\mu(a)\phi_2(a)2\gamma_2 I_{CASE,1}(t,a,v_0) - 2\gamma_{4,0} I_{ICU,0}(t,a,v_0,0{,}0)$$

$$\frac{dI_{ICU,1}(t,a,v_0,0{,}0)}{dt} = 2\gamma_{4,0} I_{ICU,0}(t,a,v_0,0{,}0) - 2\gamma_{4,0} I_{ICU,1}(t,a,v_0,0{,}0)$$

$$\frac{dI_{ICU,0}(t,a,v_0,1{,}0)}{dt} = \delta(ICU)\mu(a)\phi_2(a)2\gamma_2 I_{CASE,1}(t,a,v_0) - 2\gamma_{4,0} I_{ICU,0}(t,a,v_0,1{,}0)$$

$$\frac{dI_{ICU,1}(t,a,v_0,1{,}0)}{dt} = 2\gamma_{4,0} I_{ICU,0}(t,a,v_0,1{,}0) - 2\gamma_{4,0} I_{ICU,1}(t,a,v_0,1{,}0)$$

$$\frac{dI_{ICU,0}(t,a,v_0,0{,}1)}{dt} = (1-\delta(ICU))(1-\mu(a))\phi_2(a)2\gamma_2 I_{CASE,1}(t,v_0,a) - 2\gamma_{4,1} I_{ICU,0}(t,a,v_0,0{,}1)$$

$$\frac{dI_{ICU,1}(t,a,v_0,0{,}1)}{dt} = 2\gamma_{4,1} I_{ICU,0}(t,a,v_0,0{,}1) - 2\gamma_{4,1} I_{ICU,1}(t,a,v_0,0{,}1)$$

$$\frac{dI_{ICU,0}(t,a,v_0,1{,}1)}{dt} = \delta(ICU)(1-\mu(a))\phi_2(a)2\gamma_2 I_{CASE,1}(t,a,v_0) - 2\gamma_{4,1} I_{ICU,0}(t,a,v_0,1{,}1)$$

$$\frac{dI_{ICU,1}(t,a,v_0,1{,}1)}{dt} = 2\gamma_{4,1} I_{ICU,0}(t,a,v_0,1{,}1) - 2\gamma_{4,1} I_{ICU,1}(t,a,v_0,1{,}1)$$

$$\frac{dI_{REC,0}(t,a,v_0)}{dt} = 2\gamma_{4,1} I_{ICU,1}(t,a,v_0,0{,}1) + 2\gamma_{4,1} I_{ICU,1}(t,a,v_0,1{,}1) - 2\gamma_5 I_{REC,0}(t,a,v_0)$$

$$\frac{dI_{REC,1}(t,a,v_0)}{dt} = 2\gamma_5 I_{REC,0}(t,a,v_0) - 2\gamma_5 I_{REC,1}(t,a,v_0)$$

$$\frac{dR_1(t,a,v_0)}{dt} = \gamma_1 I_{MILD}(t,a,v_0) + 2\gamma_{3,1} I_{HOSPITAL,1}(t,a,v_0,0{,}1) + 2\gamma_{3,1} I_{HOSPITAL,1}(t,a,v_0,1{,}1) + 2\gamma_5 I_{REC,1}(t,a,v_0) + 2\gamma_{4,1} I_{ICU,1}(t,a,v_0,0{,}1) + 2\gamma_{4,1} I_{ICU,1}(t,a,v_0,1{,}1) - 2\rho R_1(t,a,v_0) - \kappa(a)R_1(t,a,v_0)$$

$$\frac{dR_2(t,a,v_0)}{dt} = 2\rho R_1(t,a,v_0) - 2\rho R_2(t,a,v_0) - \kappa(a)R_2(t,a,v_0)$$

$$\frac{dD(t,a,v_0)}{dt} = 2\gamma_{3,0} I_{HOSPITAL,1}(t,a,v_0,0{,}0) + 2\gamma_{3,0} I_{HOSPITAL,1}(t,a,v_0,1{,}0) + 2\gamma_{4,0} I_{ICU,1}(t,a,v_0,0{,}0) + 2\gamma_{4,0} I_{ICU,1}(t,a,v_0,1{,}0)$$

1.3.2 Vaccination group v₁ - vaccinated but not yet protected (state 1)

$$\frac{dS(t,a,v_1)}{dt} = \kappa(a)S(t,a,v_0) + 2\rho R_2(t,a,v_1) - \beta\frac{S(t,a,v_1)}{N}\sum_{a'} c(a,a')[\sum_{v}(I_{MILD}(t,a',v) + I_{CASE}(t,a',v))] - 2\omega S(t,a,v_1)$$

$$\frac{dE_1(t,a,v_1)}{dt} = \kappa(a)E_1(t,a,v_0) + \beta\frac{S(t,a,v_1)}{N}\sum_{a'} c(a,a')[\sum_{v}(I_{MILD}(t,a',v) + I_{CASE}(t,a',v))] - 2\alpha E_1(t,a,v_1) - 2\omega E_1(t,a,v_1)$$

$$\frac{dE_2(t,a,v_1)}{dt} = \kappa(a)E_2(t,a,v_0) + 2\alpha E_1(t,a,v_1) - 2\alpha E_2(t,a,v_1) - 2\omega E_2(t,a,v_1)$$

$$\frac{dI_{MILD}(t,a,v_1)}{dt} = (1-\phi_1(a))(2\alpha E_2(t,a,v_1)) - \gamma_1 I_{MILD}(t,a,v_1) - 2\omega I_{MILD}(t,a,v_1)$$

$$\frac{dI_{CASE,0}(t,a,v_1)}{dt} = \phi_1(a)(2\alpha E_2(t,a,v_1)) - 2\gamma_2 I_{CASE,0}(t,a,v_1) - 2\omega I_{CASE,0}(t,a,v_1)$$

$$\frac{dI_{CASE,1}(t,a,v_1)}{dt} = 2\gamma_2 I_{CASE,0}(t,a,v_1) - 2\gamma_2 I_{CASE,1}(t,a,v_1) - 2\omega I_{CASE,1}(t,a,v_1)$$

$$\frac{dI_{HOSPITAL,0}(t,a,v_1,0{,}0)}{dt} = (1-\delta(H))\mu(a)(1-\phi_2(a))2\gamma_2 I_{CASE,1}(t,a,v_1) - 2\gamma_{3,0} I_{HOSPITAL,0}(t,a,v_1,0{,}0) - 2\omega I_{HOSPITAL,0}(t,a,v_1,0{,}0)$$

$$\frac{dI_{HOSPITAL,1}(t,a,v_1,0{,}0)}{dt} = 2\gamma_{3,0} I_{HOSPITAL,0}(t,a,v_1,0{,}0) - 2\gamma_{3,0} I_{HOSPITAL,1}(t,a,v_1,0{,}0) - 2\omega I_{HOSPITAL,1}(t,a,v_1,0{,}0)$$

$$\frac{dI_{HOSPITAL,0}(t,a,v_1,1{,}0)}{dt} = \delta(H)\mu(a)(1-\phi_2(a))2\gamma_2 I_{CASE,1}(t,a,v_1) - 2\gamma_{3,0} I_{HOSPITAL,0}(t,a,v_1,1{,}0) - 2\omega I_{HOSPITAL,0}(t,a,v_1,1{,}0)$$

$$\frac{dI_{HOSPITAL,1}(t,a,v_1,1{,}0)}{dt} = 2\gamma_{3,0} I_{HOSPITAL,0}(t,a,v_1,1{,}0) - 2\gamma_{3,0} I_{HOSPITAL,1}(t,a,v_1,1{,}0) - 2\omega I_{HOSPITAL,1}(t,a,v_1,1{,}0)$$

$$\frac{dI_{HOSPITAL,0}(t,a,v_1,0{,}1)}{dt} = (1-\delta(H))(1-\mu(a))(1-\phi_2(a))2\gamma_2 I_{CASE,1}(t,a,v_1) - 2\gamma_{3,1} I_{HOSPITAL,0}(t,a,v_1,0{,}1) - 2\omega I_{HOSPITAL,0}(t,a,v_1,0{,}1)$$

$$\frac{dI_{HOSPITAL,1}(t,a,v_1,0{,}1)}{dt} = 2\gamma_{3,1} I_{HOSPITAL,0}(t,a,v_1,0{,}1) - 2\gamma_{3,1} I_{HOSPITAL,1}(t,a,v_1,0{,}1) - 2\omega I_{HOSPITAL,1}(t,a,v_1,0{,}1)$$

$$\frac{dI_{HOSPITAL,0}(t,a,v_1,1{,}1)}{dt} = \delta(H)(1-\mu(a))(1-\phi_2(a))2\gamma_2 I_{CASE,1}(t,a,v_1) - 2\gamma_{3,1} I_{HOSPITAL,0}(t,a,v_1,1{,}1) - 2\omega I_{HOSPITAL,0}(t,a,v_1,1{,}1)$$

$$\frac{dI_{HOSPITAL,1}(t,a,v_1,1{,}1)}{dt} = 2\gamma_{3,1} I_{HOSPITAL,0}(t,a,v_1,1{,}1) - 2\gamma_{3,1} I_{HOSPITAL,1}(t,a,v_1,1{,}1) - 2\omega I_{HOSPITAL,1}(t,a,v_1,1{,}1)$$

$$\frac{dI_{ICU,0}(t,a,v_1,0{,}0)}{dt} = (1-\delta(ICU))\mu(a)\phi_2(a)2\gamma_2 I_{CASE,1}(t,a,v_1) - 2\gamma_{4,0} I_{ICU,0}(t,a,v_1,0{,}0) - 2\omega I_{ICU,0}(t,a,v_1,0{,}0)$$

$$\frac{dI_{ICU,1}(t,a,v_1,0{,}0)}{dt} = 2\gamma_{4,0} I_{ICU,0}(t,a,v_1,0{,}0) - 2\gamma_{4,0} I_{ICU,1}(t,a,v_1,0{,}0) - 2\omega I_{ICU,1}(t,a,v_1,0{,}0)$$

$$\frac{dI_{ICU,0}(t,a,v_1,1{,}0)}{dt} = \delta(ICU)\mu(a)\phi_2(a)2\gamma_2 I_{CASE,1}(t,a,v_1) - 2\gamma_{4,0} I_{ICU,0}(t,a,v_1,1{,}0) - 2\omega I_{ICU,0}(t,a,v_1,1{,}0)$$

$$\frac{dI_{ICU,1}(t,a,v_1,1{,}0)}{dt} = 2\gamma_{4,0} I_{ICU,0}(t,a,v_1,1{,}0) - 2\gamma_{4,0} I_{ICU,1}(t,a,v_1,1{,}0) - 2\omega I_{ICU,1}(t,a,v_1,1{,}0)$$

$$\frac{dI_{ICU,0}(t,a,v_1,0{,}1)}{dt} = (1-\delta(ICU))(1-\mu(a))\phi_2(a)2\gamma_2 I_{CASE,1}(t,v_1,a) - 2\gamma_{4,1} I_{ICU,0}(t,a,v_1,0{,}1) - 2\omega I_{ICU,0}(t,a,v_1,0{,}1)$$

$$\frac{dI_{ICU,1}(t,a,v_1,0{,}1)}{dt} = 2\gamma_{4,1} I_{ICU,0}(t,a,v_1,0{,}1) - 2\gamma_{4,1} I_{ICU,1}(t,a,v_1,0{,}1) - 2\omega I_{ICU,1}(t,a,v_1,0{,}1)$$

$$\frac{dI_{ICU,0}(t,a,v_1,1{,}1)}{dt} = \delta(ICU)(1-\mu(a))\phi_2(a)2\gamma_2 I_{CASE,1}(t,a,v_1) - 2\gamma_{4,1} I_{ICU,0}(t,a,v_1,1{,}1) - 2\omega I_{ICU,0}(t,a,v_1,1{,}1)$$

$$\frac{dI_{ICU,1}(t,a,v_1,1{,}1)}{dt} = 2\gamma_{4,1} I_{ICU,0}(t,a,v_1,1{,}1) - 2\gamma_{4,1} I_{ICU,1}(t,a,v_1,1{,}1) - 2\omega I_{ICU,1}(t,a,v_1,1{,}1)$$

$$\frac{dI_{REC,0}(t,a,v_1)}{dt} = 2\gamma_{4,1} I_{ICU,1}(t,a,v_1,0{,}1) + 2\gamma_{4,1} I_{ICU,1}(t,a,v_1,1{,}1) - 2\gamma_5 I_{REC,0}(t,a,v_1) - 2\omega I_{REC,0}(t,a,v_1)$$

$$\frac{dI_{REC,1}(t,a,v_1)}{dt} = 2\gamma_5 I_{REC,0}(t,a,v_1) - 2\gamma_5 I_{REC,1}(t,a,v_1) - 2\omega I_{REC,1}(t,a,v_1)$$

$$\frac{dR_1(t,a,v_1)}{dt} = \kappa(a)R_1(t,a,v_0) + \gamma_1 I_{MILD}(t,a,v_1) + 2\gamma_{3,1} I_{HOSPITAL,1}(t,a,v_1,0{,}1) + 2\gamma_{3,1} I_{HOSPITAL,1}(t,a,v_1,1{,}1) + 2\gamma_5 I_{REC,1}(t,a,v_1) + 2\gamma_{4,1} I_{ICU,1}(t,a,v_1,0{,}1) + 2\gamma_{4,1} I_{ICU,1}(t,a,v_1,1{,}1) - 2\rho R_1(t,a,v_1) - 2\omega R(t,a,v_1)$$

$$\frac{dR_2(t,a,v_1)}{dt} = \kappa(a)R_2(t,a,v_0) + 2\rho R_1(t,a,v_1) - 2\rho R_2(t,a,v_1)$$

$$\frac{dD(t,a,v_1)}{dt} = 2\gamma_{3,0} I_{HOSPITAL,1}(t,a,v_1,0{,}0) + 2\gamma_{3,0} I_{HOSPITAL,1}(t,a,v_1,1{,}0) + 2\gamma_{4,0} I_{ICU,1}(t,a,v_1,0{,}0) + 2\gamma_{4,0} I_{ICU,1}(t,a,v_1,1{,}0)$$

1.3.3 Vaccination group v₂ - vaccinated but not yet protected (state 2)

$$\frac{dS(t,a,v_2)}{dt} = 2\rho R_2(t,a,v_2) - \beta\frac{S(t,a,v_2)}{N}\sum_{a'} c(a,a')[\sum_{v}(I_{MILD}(t,a',v) + I_{CASE}(t,a',v))] + 2\omega S(t,a,v_1) - 2\omega S(t,a,v_2)$$

$$\frac{dE_1(t,a,v_2)}{dt} = \beta\frac{S(t,a,v_2)}{N}\sum_{a'} c(a,a')[\sum_{v}(I_{MILD}(t,a',v) + I_{CASE}(t,a',v))] - 2\alpha E_1(t,a,v_2) + 2\omega E_1(t,a,v_1) - 2\omega E_1(t,a,v_2)$$

$$\frac{dE_2(t,a,v_2)}{dt} = 2\alpha E_1(t,a,v_2) - 2\alpha E_2(t,a,v_2) + 2\omega E_2(t,a,v_1) - 2\omega E_2(t,a,v_2)$$

$$\frac{dI_{MILD}(t,a,v_2)}{dt} = (1-\phi_1(a))(2\alpha E_2(t,a,v_2)) - \gamma_1 I_{MILD}(t,a,v_2) + 2\omega I_{MILD}(t,a,v_1) - 2\omega I_{MILD}(t,a,v_2)$$

$$\frac{dI_{CASE,0}(t,a,v_2)}{dt} = \phi_1(a)(2\alpha E_2(t,a,v_2)) - 2\gamma_2 I_{CASE,0}(t,a,v_2) + 2\omega I_{CASE,0}(t,a,v_1) - 2\omega I_{CASE,0}(t,a,v_2)$$

$$\frac{dI_{CASE,1}(t,a,v_2)}{dt} = 2\gamma_2 I_{CASE,0}(t,a,v_2) - 2\gamma_2 I_{CASE,1}(t,a,v_2) + 2\omega I_{CASE,1}(t,a,v_1) - 2\omega I_{CASE,1}(t,a,v_2)$$

$$\frac{dI_{HOSPITAL,0}(t,a,v_2,0,0)}{dt} = (1-\delta(H))\mu(a)(1-\phi_2(a))2\gamma_2 I_{CASE,1}(t,a,v_2) - 2\gamma_{3,0} I_{HOSPITAL,0}(t,a,v_2,0,0) + 2\omega I_{HOSPITAL,0}(t,a,v_1,0,0) - 2\omega I_{HOSPITAL,0}(t,a,v_2,0,0)$$

$$\frac{dI_{HOSPITAL,1}(t,a,v_2,0,0)}{dt} = 2\gamma_{3,0} I_{HOSPITAL,0}(t,a,v_2,0,0) - 2\gamma_{3,0} I_{HOSPITAL,1}(t,a,v_2,0,0) + 2\omega I_{HOSPITAL,1}(t,a,v_1,0,0) - 2\omega I_{HOSPITAL,1}(t,a,v_2,0,0)$$

$$\frac{dI_{HOSPITAL,0}(t,a,v_2,1,0)}{dt} = \delta(H)\mu(a)(1-\phi_2(a))2\gamma_2 I_{CASE,1}(t,a,v_2) - 2\gamma_{3,0} I_{HOSPITAL,0}(t,a,v_2,1,0) + 2\omega I_{HOSPITAL,0}(t,a,v_1,1,0) - 2\omega I_{HOSPITAL,0}(t,a,v_2,1,0)$$

$$\frac{dI_{HOSPITAL,1}(t,a,v_2,1,0)}{dt} = 2\gamma_{3,0} I_{HOSPITAL,0}(t,a,v_2,1,0) - 2\gamma_{3,0} I_{HOSPITAL,1}(t,a,v_2,1,0) + 2\omega I_{HOSPITAL,1}(t,a,v_1,1,0) - 2\omega I_{HOSPITAL,1}(t,a,v_2,1,0)$$

$$\frac{dI_{HOSPITAL,0}(t,a,v_2,0,1)}{dt} = (1-\delta(H))(1-\mu(a))(1-\phi_2(a))2\gamma_2 I_{CASE,1}(t,a,v_2) - 2\gamma_{3,1} I_{HOSPITAL,0}(t,a,v_2,0,1) + 2\omega I_{HOSPITAL,0}(t,a,v_1,0,1) - 2\omega I_{HOSPITAL,0}(t,a,v_2,0,1)$$

$$\frac{dI_{HOSPITAL,1}(t,a,v_2,0,1)}{dt} = 2\gamma_{3,1} I_{HOSPITAL,0}(t,a,v_2,0,1) - 2\gamma_{3,1} I_{HOSPITAL,1}(t,a,v_2,0,1) + 2\omega I_{HOSPITAL,1}(t,a,v_1,0,1) - 2\omega I_{HOSPITAL,1}(t,a,v_2,0,1)$$

$$\frac{dI_{HOSPITAL,0}(t,a,v_2,1,1)}{dt} = \delta(H)(1-\mu(a))(1-\phi_2(a))2\gamma_2 I_{CASE,1}(t,a,v_2) - 2\gamma_{3,1} I_{HOSPITAL,0}(t,a,v_2,1,1) + 2\omega I_{HOSPITAL,0}(t,a,v_1,1,1) - 2\omega I_{HOSPITAL,0}(t,a,v_2,1,1)$$

$$\frac{dI_{HOSPITAL,1}(t,a,v_2,1,1)}{dt} = 2\gamma_{3,1} I_{HOSPITAL,0}(t,a,v_2,1,1) - 2\gamma_{3,1} I_{HOSPITAL,1}(t,a,v_2,1,1) + 2\omega I_{HOSPITAL,1}(t,a,v_1,1,1) - 2\omega I_{HOSPITAL,1}(t,a,v_2,1,1)$$

$$\frac{dI_{ICU,0}(t,a,v_2,0,0)}{dt} = (1-\delta(ICU))\mu(a)\phi_2(a)2\gamma_2 I_{CASE,1}(t,a,v_2) - 2\gamma_{4,0} I_{ICU,0}(t,a,v_2,0,0) + 2\omega I_{ICU,0}(t,a,v_1,0,0) - 2\omega I_{ICU,0}(t,a,v_2,0,0)$$

$$\frac{dI_{ICU,1}(t,a,v_2,0,0)}{dt} = 2\gamma_{4,0} I_{ICU,0}(t,a,v_2,0,0) - 2\gamma_{4,0} I_{ICU,1}(t,a,v_2,0,0) + 2\omega I_{ICU,1}(t,a,v_1,0,0) - 2\omega I_{ICU,1}(t,a,v_2,0,0)$$

$$\frac{dI_{ICU,0}(t,a,v_2,1,0)}{dt} = \delta(ICU)\mu(a)\phi_2(a)2\gamma_2 I_{CASE,1}(t,a,v_2) - 2\gamma_{4,0} I_{ICU,0}(t,a,v_2,1,0) + 2\omega I_{ICU,0}(t,a,v_1,1,0) - 2\omega I_{ICU,0}(t,a,v_2,1,0)$$

$$\frac{dI_{ICU,1}(t,a,v_2,1{,}0)}{dt} = 2\gamma_{4,0}I_{ICU,0}(t,a,v_2,1{,}0) - 2\gamma_{4,0}I_{ICU,1}(t,a,v_2,1{,}0) + 2\omega I_{ICU,1}(t,a,v_1,1{,}0) - 2\omega I_{ICU,1}(t,a,v_2,1{,}0)$$

$$\frac{dI_{ICU,0}(t,a,v_2,0{,}1)}{dt} = (1-\delta(ICU))(1-\mu(a))\phi_2(a)2\gamma_2 I_{CASE,1}(t,v_2,a) - 2\gamma_{4,1}I_{ICU,0}(t,a,v_2,0{,}1) + 2\omega I_{ICU,0}(t,a,v_1,0{,}1) - 2\omega I_{ICU,0}(t,a,v_2,0{,}1)$$

$$\frac{dI_{ICU,1}(t,a,v_2,0{,}1)}{dt} = 2\gamma_{4,1}I_{ICU,0}(t,a,v_2,0{,}1) - 2\gamma_{4,1}I_{ICU,1}(t,a,v_2,0{,}1) + 2\omega I_{ICU,1}(t,a,v_1,0{,}1) - 2\omega I_{ICU,1}(t,a,v_2,0{,}1)$$

$$\frac{dI_{ICU,0}(t,a,v_2,1{,}1)}{dt} = \delta(ICU)(1-\mu(a))\phi_2(a)2\gamma_2 I_{CASE,1}(t,a,v_2) - 2\gamma_{4,1}I_{ICU,0}(t,a,v_2,1{,}1) + 2\omega I_{ICU,0}(t,a,v_1,1{,}1) - 2\omega I_{ICU,0}(t,a,v_2,1{,}1)$$

$$\frac{dI_{ICU,1}(t,a,v_2,1{,}1)}{dt} = 2\gamma_{4,1}I_{ICU,0}(t,a,v_2,1{,}1) - 2\gamma_{4,1}I_{ICU,1}(t,a,v_2,1{,}1) + 2\omega I_{ICU,1}(t,a,v_1,1{,}1) - 2\omega I_{ICU,1}(t,a,v_2,1{,}1)$$

$$\frac{dI_{REC,0}(t,a,v_2)}{dt} = 2\gamma_{4,1}I_{ICU,1}(t,a,v_2,0{,}1) + 2\gamma_{4,1}I_{ICU,1}(t,a,v_2,1{,}1) - 2\gamma_5 I_{REC,0}(t,a,v_2) + 2\omega I_{REC,0}(t,a,v_1) - 2\omega I_{REC,0}(t,a,v_2)$$

$$\frac{dI_{REC,1}(t,a,v_2)}{dt} = 2\gamma_5 I_{REC,0}(t,a,v_2) - 2\gamma_5 I_{REC,1}(t,a,v_2) + 2\omega I_{REC,1}(t,a,v_1) - 2\omega I_{REC,1}(t,a,v_2)$$

$$\frac{dR_1(t,a,v_2)}{dt} = \gamma_1 I_{MILD}(t,a,v_2) + 2\gamma_{3,1}I_{HOSPITAL,1}(t,a,v_2,0{,}1) + 2\gamma_{3,1}I_{HOSPITAL,1}(t,a,v_2,1{,}1) + 2\gamma_5 I_{REC,1}(t,a,v_2) + 2\gamma_{4,1}I_{ICU,1}(t,a,v_2,0{,}1) + 2\gamma_{4,1}I_{ICU,1}(t,a,v_2,1{,}1) - 2\rho R_1(t,a,v_2) + 2\omega R_1(t,a,v_1) - 2\omega R_1(t,a,v_2)$$

$$\frac{dR_2(t,a,v_2)}{dt} = 2\rho R_1(t,a,v_2) - 2\rho R_2(t,a,v_2) + 2\omega R_2(t,a,v_1) - 2\omega R_2(t,a,v_2)$$

$$\frac{dD(t,a,v_2)}{dt} = 2\gamma_{3,0}I_{HOSPITAL,1}(t,a,v_2,0{,}0) + 2\gamma_{3,0}I_{HOSPITAL,1}(t,a,v_2,1{,}0) + 2\gamma_{4,0}I_{ICU,1}(t,a,v_2,0{,}0) + 2\gamma_{4,0}I_{ICU,1}(t,a,v_2,1{,}0)$$

1.3.4 Vaccination group v₃ - vaccinated and protected (state 1)

$$\frac{dS(t,a,v_3)}{dt} = 2\rho R_2(t,a,v_3) - v_{inf}(a)\beta\frac{S(t,a,v_3)}{N}\sum_{a'} c(a,a')[\sum_{v}\left(I_{MILD}(t,a',v) + I_{CASE}(t,a',v)\right)] + 2\omega S(t,a,v_2) - 2\psi S(t,a,v_3)$$

$$\frac{dE_1(t,a,v_3)}{dt} = v_{inf}(a)\beta\frac{S(t,a,v_3)}{N}\sum_{a'} c(a,a')[\sum_{v}(I_{MILD}(t,a',v) + I_{CASE}(t,a',v))] - 2\alpha E_1(t,a,v_2) + 2\omega E_1(t,a,v_2) - 2\psi E_1(t,a,v_3)$$

$$\frac{dE_2(t,a,v_3)}{dt} = 2\alpha E_1(t,a,v_3) - 2\alpha E_2(t,a,v_3) + 2\omega E_2(t,a,v_2) - 2\psi E_2(t,a,v_3)$$

$$\frac{dI_{MILD}(t,a,v_3)}{dt} = (1 - v_{dis}(a)\phi_1(a))(2\alpha E_2(t,a,v_3)) - \gamma_1 I_{MILD}(t,a,v_3) + 2\omega I_{MILD}(t,a,v_2) - 2\psi I_{MILD}(t,a,v_3)$$

$$\frac{dI_{CASE,0}(t,a,v_3)}{dt} = v_{dis}(a)\phi_1(a)(2\alpha E_2(t,a,v_3)) - 2\gamma_2 I_{CASE,0}(t,a,v_3) + 2\omega I_{CASE,0}(t,a,v_2) - 2\psi I_{CASE,0}(t,a,v_3)$$

$$\frac{dI_{CASE,1}(t,a,v_3)}{dt} = 2\gamma_2 I_{CASE,0}(t,a,v_3) - 2\gamma_2 I_{CASE,1}(t,a,v_3) + 2\omega I_{CASE,1}(t,a,v_2) - 2\psi I_{CASE,1}(t,a,v_3)$$

$$\frac{dI_{HOSPITAL,0}(t,a,v_3,0,0)}{dt} = (1-\delta(H))\mu(a)(1-\phi_2(a))2\gamma_2 I_{CASE,1}(t,a,v_3) - 2\gamma_{3,0} I_{HOSPITAL,0}(t,a,v_3,0,0) + 2\omega I_{HOSPITAL,0}(t,a,v_2,0,0) - 2\psi I_{HOSPITAL,0}(t,a,v_3,0,0)$$

$$\frac{dI_{HOSPITAL,1}(t,a,v_3,0,0)}{dt} = 2\gamma_{3,0} I_{HOSPITAL,0}(t,a,v_3,0,0) - 2\gamma_{3,0} I_{HOSPITAL,1}(t,a,v_3,0,0) + 2\omega I_{HOSPITAL,1}(t,a,v_2,0,0) - 2\psi I_{HOSPITAL,1}(t,a,v_3,0,0)$$

$$\frac{dI_{HOSPITAL,0}(t,a,v_3,1,0)}{dt} = \delta(H)\mu(a)(1-\phi_2(a))2\gamma_2 I_{CASE,1}(t,a,v_3) - 2\gamma_{3,0} I_{HOSPITAL,0}(t,a,v_3,1,0) + 2\omega I_{HOSPITAL,0}(t,a,v_2,1,0) - 2\psi I_{HOSPITAL,0}(t,a,v_3,1,0)$$

$$\frac{dI_{HOSPITAL,1}(t,a,v_3,1,0)}{dt} = 2\gamma_{3,0} I_{HOSPITAL,0}(t,a,v_3,1,0) - 2\gamma_{3,0} I_{HOSPITAL,1}(t,a,v_3,1,0) + 2\omega I_{HOSPITAL,1}(t,a,v_2,1,0) - 2\psi I_{HOSPITAL,1}(t,a,v_3,1,0)$$

$$\frac{dI_{HOSPITAL,0}(t,a,v_3,0,1)}{dt} = (1-\delta(H))(1-\mu(a))(1-\phi_2(a))2\gamma_2 I_{CASE,1}(t,a,v_3) - 2\gamma_{3,1} I_{HOSPITAL,0}(t,a,v_3,0,1) + 2\omega I_{HOSPITAL,0}(t,a,v_2,0,1) - 2\psi I_{HOSPITAL,0}(t,a,v_3,0,1)$$

$$\frac{dI_{HOSPITAL,1}(t,a,v_3,0,1)}{dt} = 2\gamma_{3,1} I_{HOSPITAL,0}(t,a,v_3,0,1) - 2\gamma_{3,1} I_{HOSPITAL,1}(t,a,v_3,0,1) + 2\omega I_{HOSPITAL,1}(t,a,v_2,0,1) - 2\psi I_{HOSPITAL,1}(t,a,v_3,0,1)$$

$$\frac{dI_{HOSPITAL,0}(t,a,v_3,1,1)}{dt} = \delta(H)(1-\mu(a))(1-\phi_2(a))2\gamma_2 I_{CASE,1}(t,a,v_3) - 2\gamma_{3,1} I_{HOSPITAL,0}(t,a,v_3,1,1) + 2\omega I_{HOSPITAL,0}(t,a,v_2,1,1) - 2\psi I_{HOSPITAL,0}(t,a,v_3,1,1)$$

$$\frac{dI_{HOSPITAL,1}(t,a,v_3,1,1)}{dt} = 2\gamma_{3,1} I_{HOSPITAL,0}(t,a,v_3,1,1) - 2\gamma_{3,1} I_{HOSPITAL,1}(t,a,v_3,1,1) + 2\omega I_{HOSPITAL,1}(t,a,v_2,1,1) - 2\psi I_{HOSPITAL,1}(t,a,v_3,1,1)$$

$$\frac{dI_{ICU,0}(t,a,v_3,0,0)}{dt} = (1-\delta(ICU))\mu(a)\phi_2(a)2\gamma_2 I_{CASE,1}(t,a,v_3) - 2\gamma_{4,0} I_{ICU,0}(t,a,v_3,0,0) + 2\omega I_{ICU,0}(t,a,v_2,0,0) - 2\psi I_{ICU,0}(t,a,v_3,0,0)$$

$$\frac{dI_{ICU,1}(t,a,v_3,0,0)}{dt} = 2\gamma_{4,0} I_{ICU,0}(t,a,v_3,0,0) - 2\gamma_{4,0} I_{ICU,1}(t,a,v_3,0,0) + 2\omega I_{ICU,1}(t,a,v_2,0,0) - 2\psi I_{ICU,1}(t,a,v_3,0,0)$$

$$\frac{dI_{ICU,0}(t,a,v_3,1,0)}{dt} = \delta(ICU)\mu(a)\phi_2(a)2\gamma_2 I_{CASE,1}(t,a,v_3) - 2\gamma_{4,0} I_{ICU,0}(t,a,v_3,1,0) + 2\omega I_{ICU,0}(t,a,v_2,1,0) - 2\psi I_{ICU,0}(t,a,v_3,1,0)$$

$$\frac{dI_{ICU,1}(t,a,v_3,1{,}0)}{dt} = 2\gamma_{4,0}I_{ICU,0}(t,a,v_3,1{,}0) - 2\gamma_{4,0}I_{ICU,1}(t,a,v_3,1{,}0) + 2\omega I_{ICU,1}(t,a,v_2,1{,}0) - 2\psi I_{ICU,1}(t,a,v_3,1{,}0)$$

$$\frac{dI_{ICU,0}(t,a,v_3,0{,}1)}{dt} = (1-\delta(ICU))(1-\mu(a))\phi_2(a)2\gamma_2 I_{CASE,1}(t,v_3,a) - 2\gamma_{4,1}I_{ICU,0}(t,a,v_3,0{,}1) + 2\omega I_{ICU,0}(t,a,v_2,0{,}1) - 2\psi I_{ICU,0}(t,a,v_3,0{,}1)$$

$$\frac{dI_{ICU,1}(t,a,v_3,0{,}1)}{dt} = 2\gamma_{4,1}I_{ICU,0}(t,a,v_3,0{,}1) - 2\gamma_{4,1}I_{ICU,1}(t,a,v_3,0{,}1) + 2\omega I_{ICU,1}(t,a,v_2,0{,}1) - 2\psi I_{ICU,1}(t,a,v_3,0{,}1)$$

$$\frac{dI_{ICU,0}(t,a,v_3,1{,}1)}{dt} = \delta(ICU)(1-\mu(a))\phi_2(a)2\gamma_2 I_{CASE,1}(t,a,v_3) - 2\gamma_{4,1}I_{ICU,0}(t,a,v_3,1{,}1) + 2\omega I_{ICU,0}(t,a,v_2,1{,}1) - 2\psi I_{ICU,0}(t,a,v_3,1{,}1)$$

$$\frac{dI_{ICU,1}(t,a,v_3,1{,}1)}{dt} = 2\gamma_{4,1}I_{ICU,0}(t,a,v_3,1{,}1) - 2\gamma_{4,1}I_{ICU,1}(t,a,v_3,1{,}1) + 2\omega I_{ICU,1}(t,a,v_2,1{,}1) - 2\psi I_{ICU,1}(t,a,v_3,1{,}1)$$

$$\frac{dI_{REC,0}(t,a,v_3)}{dt} = 2\gamma_{4,1}I_{ICU,1}(t,a,v_3,0{,}1) + 2\gamma_{4,1}I_{ICU,1}(t,a,v_3,1{,}1) - 2\gamma_5 I_{REC,0}(t,a,v_3) + 2\omega I_{REC,0}(t,a,v_2) - 2\psi I_{REC,0}(t,a,v_3)$$

$$\frac{dI_{REC,1}(t,a,v_3)}{dt} = 2\gamma_5 I_{REC,0}(t,a,v_3) - 2\gamma_5 I_{REC,1}(t,a,v_3) + 2\omega I_{REC,1}(t,a,v_2) - 2\psi I_{REC,1}(t,a,v_3)$$

$$\frac{dR_1(t,a,v_3)}{dt} = \gamma_1 I_{MILD}(t,a,v_3) + 2\gamma_{3,1}I_{HOSPITAL,1}(t,a,v_3,0{,}1) + 2\gamma_{3,1}I_{HOSPITAL,1}(t,a,v_3,1{,}1) + 2\gamma_5 I_{REC,1}(t,a,v_3) + 2\gamma_{4,1}I_{ICU,1}(t,a,v_3,0{,}1) + 2\gamma_{4,1}I_{ICU,1}(t,a,v_3,1{,}1) - 2\rho R_1(t,a,v_3) + 2\omega R_1(t,a,v_2) - 2\psi R_1(t,a,v_3)$$

$$\frac{dR_2(t,a,v_3)}{dt} = 2\rho R_1(t,a,v_3) - 2\rho R_2(t,a,v_3) + 2\omega R_2(t,a,v_2) - 2\psi R_2(t,a,v_3)$$

$$\frac{dD(t,a,v_3)}{dt} = 2\gamma_{3,0}I_{HOSPITAL,1}(t,a,v_3,0{,}0) + 2\gamma_{3,0}I_{HOSPITAL,1}(t,a,v_3,1{,}0) + 2\gamma_{4,0}I_{ICU,1}(t,a,v_3,0{,}0) + 2\gamma_{4,0}I_{ICU,1}(t,a,v_3,1{,}0)$$

1.3.5 Vaccination group v₄ - vaccinated and protected (state 2)

$$\frac{dS(t,a,v_4)}{dt} = 2\rho R_2(t,a,v_4) - \upsilon_{inf}(a)\beta\frac{S(t,a,v_4)}{N}\sum_{a'} c(a,a')[\sum_{v}\left(I_{MILD}(t,a',v) + I_{CASE}(t,a',v)\right)] + 2\psi S(t,a,v_3) - 2\psi S(t,a,v_4)$$

$$\frac{dE_1(t,a,v_4)}{dt} = \upsilon_{inf}(a)\beta\frac{S(t,a,v_4)}{N}\sum_{a'} c(a,a')[\sum_{v}(I_{MILD}(t,a',v) + I_{CASE}(t,a',v))] - 2\alpha E_1(t,a,v_4) + 2\psi E_1(t,a,v_3) - 2\psi E_1(t,a,v_4)$$

$$\frac{dE_2(t,a,v_4)}{dt} = 2\alpha E_1(t,a,v_4) - 2\alpha E_2(t,a,v_4) + 2\psi E_2(t,a,v_3) - 2\psi E_2(t,a,v_4)$$

$$\frac{dI_{MILD}(t,a,v_4)}{dt} = (1-\upsilon_{dis}(a)\phi_1(a))(2\alpha E_2(t,a,v_4)) - \gamma_1 I_{MILD}(t,a,v_4) + 2\psi I_{MILD}(t,a,v_3) - 2\psi I_{MILD}(t,a,v_4)$$

$$\frac{dI_{CASE,0}(t,a,v_4)}{dt} = \upsilon_{dis}(a)\phi_1(a)(2\alpha E_2(t,a,v_4)) - 2\gamma_2 I_{CASE,0}(t,a,v_4) + 2\psi I_{CASE,0}(t,a,v_3) - 2\psi I_{CASE,0}(t,a,v_4)$$

$$\frac{dI_{CASE,1}(t,a,v_4)}{dt} = 2\gamma_2 I_{CASE,0}(t,a,v_4) - 2\gamma_2 I_{CASE,1}(t,a,v_4) + 2\psi I_{CASE,1}(t,a,v_3) - 2\psi I_{CASE,1}(t,a,v_4)$$

$$\frac{dI_{HOSPITAL,0}(t,a,v_4,0,0)}{dt} = (1-\delta(H))\mu(a)(1-\phi_2(a))2\gamma_2 I_{CASE,1}(t,a,v_4) - 2\gamma_{3,0} I_{HOSPITAL,0}(t,a,v_4,0,0) + 2\psi I_{HOSPITAL,0}(t,a,v_3,0,0) - 2\psi I_{HOSPITAL,0}(t,a,v_4,0,0)$$

$$\frac{dI_{HOSPITAL,1}(t,a,v_4,0,0)}{dt} = 2\gamma_{3,0} I_{HOSPITAL,0}(t,a,v_4,0,0) - 2\gamma_{3,0} I_{HOSPITAL,1}(t,a,v_4,0,0) + 2\psi I_{HOSPITAL,1}(t,a,v_3,0,0) - 2\psi I_{HOSPITAL,1}(t,a,v_4,0,0)$$

$$\frac{dI_{HOSPITAL,0}(t,a,v_4,1,0)}{dt} = \delta(H)\mu(a)(1-\phi_2(a))2\gamma_2 I_{CASE,1}(t,a,v_4) - 2\gamma_{3,0} I_{HOSPITAL,0}(t,a,v_4,1,0) + 2\psi I_{HOSPITAL,0}(t,a,v_3,1,0) - 2\psi I_{HOSPITAL,0}(t,a,v_3,1,0)$$

$$\frac{dI_{HOSPITAL,1}(t,a,v_4,1,0)}{dt} = 2\gamma_{3,0} I_{HOSPITAL,0}(t,a,v_4,1,0) - 2\gamma_{3,0} I_{HOSPITAL,1}(t,a,v_4,1,0) + 2\psi I_{HOSPITAL,1}(t,a,v_3,1,0) - 2\psi I_{HOSPITAL,1}(t,a,v_4,1,0)$$

$$\frac{dI_{HOSPITAL,0}(t,a,v_4,0,1)}{dt} = (1-\delta(H))(1-\mu(a))(1-\phi_2(a))2\gamma_2 I_{CASE,1}(t,a,v_4) - 2\gamma_{3,1} I_{HOSPITAL,0}(t,a,v_4,0,1) + 2\psi I_{HOSPITAL,0}(t,a,v_3,0,1) - 2\psi I_{HOSPITAL,0}(t,a,v_4,0,1)$$

$$\frac{dI_{HOSPITAL,1}(t,a,v_4,0,1)}{dt} = 2\gamma_{3,1} I_{HOSPITAL,0}(t,a,v_4,0,1) - 2\gamma_{3,1} I_{HOSPITAL,1}(t,a,v_4,0,1) + 2\psi I_{HOSPITAL,1}(t,a,v_3,0,1) - 2\psi I_{HOSPITAL,1}(t,a,v_4,0,1)$$

$$\frac{dI_{HOSPITAL,0}(t,a,v_4,1,1)}{dt} = \delta(H)(1-\mu(a))(1-\phi_2(a))2\gamma_2 I_{CASE,1}(t,a,v_4) - 2\gamma_{3,1} I_{HOSPITAL,0}(t,a,v_4,1,1) + 2\psi I_{HOSPITAL,0}(t,a,v_3,1,1) - 2\psi I_{HOSPITAL,0}(t,a,v_4,1,1)$$

$$\frac{dI_{HOSPITAL,1}(t,a,v_4,1,1)}{dt} = 2\gamma_{3,1} I_{HOSPITAL,0}(t,a,v_4,1,1) - 2\gamma_{3,1} I_{HOSPITAL,1}(t,a,v_4,1,1) + 2\psi I_{HOSPITAL,1}(t,a,v_3,1,1) - 2\psi I_{HOSPITAL,1}(t,a,v_4,1,1)$$

$$\frac{dI_{ICU,0}(t,a,v_4,0,0)}{dt} = (1-\delta(ICU))\mu(a)\phi_2(a)2\gamma_2 I_{CASE,1}(t,a,v_4) - 2\gamma_{4,0} I_{ICU,0}(t,a,v_4,0,0) + 2\psi I_{ICU,0}(t,a,v_3,0,0) - 2\psi I_{ICU,0}(t,a,v_4,0,0)$$

$$\frac{dI_{ICU,1}(t,a,v_4,0,0)}{dt} = 2\gamma_{4,0} I_{ICU,0}(t,a,v_4,0,0) - 2\gamma_{4,0} I_{ICU,1}(t,a,v_4,0,0) + 2\psi I_{ICU,1}(t,a,v_3,0,0) - 2\psi I_{ICU,1}(t,a,v_4,0,0)$$

$$\frac{dI_{ICU,0}(t,a,v_4,1,0)}{dt} = \delta(ICU)\mu(a)\phi_2(a)2\gamma_2 I_{CASE,1}(t,a,v_4) - 2\gamma_{4,0} I_{ICU,0}(t,a,v_4,1,0) + 2\psi I_{ICU,0}(t,a,v_3,1,0) - 2\psi I_{ICU,0}(t,a,v_4,1,0)$$

$$\frac{dI_{ICU,1}(t,a,v_4,1,0)}{dt} = 2\gamma_{4,0} I_{ICU,0}(t,a,v_4,1,0) - 2\gamma_{4,0} I_{ICU,1}(t,a,v_4,1,0) + 2\psi I_{ICU,1}(t,a,v_3,1,0) - 2\psi I_{ICU,1}(t,a,v_4,1,0)$$

$$\frac{dI_{ICU,0}(t,a,v_4,0,1)}{dt} = (1-\delta(ICU))(1-\mu(a))\phi_2(a)2\gamma_2 I_{CASE,1}(t,v_4,a) - 2\gamma_{4,1} I_{ICU,0}(t,a,v_4,0,1) + 2\psi I_{ICU,0}(t,a,v_3,0,1) - 2\psi I_{ICU,0}(t,a,v_4,0,1)$$

$$\frac{dI_{ICU,1}(t,a,v_4,0,1)}{dt} = 2\gamma_{4,1} I_{ICU,0}(t,a,v_4,0,1) - 2\gamma_{4,1} I_{ICU,1}(t,a,v_4,0,1) + 2\psi I_{ICU,1}(t,a,v_3,0,1) - 2\psi I_{ICU,1}(t,a,v_4,0,1)$$

$$\frac{dI_{ICU,0}(t,a,v_4,1,1)}{dt} = \delta(ICU)(1-\mu(a))\phi_2(a)2\gamma_2 I_{CASE,1}(t,a,v_4) - 2\gamma_{4,1} I_{ICU,0}(t,a,v_4,1,1) + 2\psi I_{ICU,0}(t,a,v_3,1,1) - 2\psi I_{ICU,0}(t,a,v_4,1,1)$$

$$\frac{dI_{ICU,1}(t,a,v_4,1,1)}{dt} = 2\gamma_{4,1} I_{ICU,0}(t,a,v_4,1,1) - 2\gamma_{4,1} I_{ICU,1}(t,a,v_4,1,1) + 2\psi I_{ICU,1}(t,a,v_3,1,1) - 2\psi I_{ICU,1}(t,a,v_4,1,1)$$

$$\frac{dI_{REC,0}(t,a,v_4)}{dt} = 2\gamma_{4,1} I_{ICU,1}(t,a,v_4,0,1) + 2\gamma_{4,1} I_{ICU,1}(t,a,v_4,1,1) - 2\gamma_5 I_{REC,0}(t,a,v_4) + 2\psi I_{REC,0}(t,a,v_3) - 2\psi I_{REC,0}(t,a,v_4)$$

$$\frac{dI_{REC,1}(t,a,v_4)}{dt} = 2\gamma_5 I_{REC,0}(t,a,v_4) - 2\gamma_5 I_{REC,1}(t,a,v_4) + 2\psi I_{REC,1}(t,a,v_3) - 2\psi I_{REC,1}(t,a,v_4)$$

$$\frac{dR_1(t,a,v_4)}{dt} = \gamma_1 I_{MILD}(t,a,v_4) + 2\gamma_{3,1} I_{HOSPITAL,1}(t,a,v_4,0,1) + 2\gamma_{3,1} I_{HOSPITAL,1}(t,a,v_4,1,1) + 2\gamma_5 I_{REC,1}(t,a,v_4) + 2\gamma_{4,1} I_{ICU,1}(t,a,v_4,0,1) + 2\gamma_{4,1} I_{ICU,1}(t,a,v_4,1,1) - 2\rho R_1(t,a,v_4) + 2\psi R_1(t,a,v_3) - 2\psi R_1(t,a,v_4)$$

$$\frac{dR_2(t,a,v_4)}{dt} = 2\rho R_1(t,a,v_4) - 2\rho R_2(t,a,v_4) + 2\psi R_2(t,a,v_3) - 2\psi R_2(t,a,v_4)$$

$$\frac{dD(t,a,v_4)}{dt} = 2\gamma_{3,0} I_{HOSPITAL,1}(t,a,v_4,0,0) + 2\gamma_{3,0} I_{HOSPITAL,1}(t,a,v_4,1,0) + 2\gamma_{4,0} I_{ICU,1}(t,a,v_4,0,0) + 2\gamma_{4,0} I_{ICU,1}(t,a,v_4,1,0)$$

1.3.6 Vaccination group v_5 - previously vaccinated but no longer protected

$$\frac{dS(t,a,v_5)}{dt} = 2\rho R_2(t,a,v_5) - \beta\frac{S(t,a,v_5)}{N}\sum_{a'} c(a,a')[\sum_{v}(I_{MILD}(t,a',v) + I_{CASE}(t,a',v))] + 2\psi S(t,a,v_4)$$

$$\frac{dE_1(t,a,v_5)}{dt} = \beta\frac{S(t,a,v_5)}{N}\sum_{a'} c(a,a')[\sum_{v}(I_{MILD}(t,a',v) + I_{CASE}(t,a',v))] - 2\alpha E_1(t,a,v_5) + 2\psi E_1(t,a,v_4)$$

$$\frac{dE_2(t,a,v_5)}{dt} = 2\alpha E_1(t,a,v_5) - 2\alpha E_2(t,a,v_5) + 2\psi E_2(t,a,v_4)$$

$$\frac{dI_{MILD}(t,a,v_5)}{dt} = (1-\phi_1(a))(2\alpha E_2(t,a,v_5)) - \gamma_1 I_{MILD}(t,a,v_5) + 2\psi I_{MILD}(t,a,v_4)$$

$$\frac{dI_{CASE,0}(t,a,v_5)}{dt} = \phi_1(a)(2\alpha E_2(t,a,v_5)) - 2\gamma_2 I_{CASE,0}(t,a,v_5) + 2\psi I_{CASE,0}(t,a,v_4)$$

$$\frac{dI_{CASE,1}(t,a,v_5)}{dt} = 2\gamma_2 I_{CASE,0}(t,a,v_5) - 2\gamma_2 I_{CASE,1}(t,a,v_5) + 2\psi I_{CASE,1}(t,a,v_4)$$

$$\frac{dI_{HOSPITAL,0}(t,a,v_5,0,0)}{dt} = (1-\delta(H))\mu(a)(1-\phi_2(a))2\gamma_2 I_{CASE,1}(t,a,v_5) - 2\gamma_{3,0} I_{HOSPITAL,0}(t,a,v_5,0,0) + 2\psi I_{HOSPITAL,0}(t,a,v_4,0,0)$$

$$\frac{dI_{HOSPITAL,1}(t,a,v_5,0,0)}{dt} = 2\gamma_{3,0} I_{HOSPITAL,0}(t,a,v_5,0,0) - 2\gamma_{3,0} I_{HOSPITAL,1}(t,a,v_5,0,0) + 2\psi I_{HOSPITAL,1}(t,a,v_4,0,0)$$

$$\frac{dI_{HOSPITAL,0}(t,a,v_5,1,0)}{dt} = \delta(H)\mu(a)(1-\phi_2(a))2\gamma_2 I_{CASE,1}(t,a,v_5) - 2\gamma_{3,0} I_{HOSPITAL,0}(t,a,v_5,1,0) + 2\psi I_{HOSPITAL,0}(t,a,v_3,1,0)$$

$$\frac{dI_{HOSPITAL,1}(t,a,v_5,1,0)}{dt} = 2\gamma_{3,0} I_{HOSPITAL,0}(t,a,v_5,1,0) - 2\gamma_{3,0} I_{HOSPITAL,1}(t,a,v_5,1,0) + 2\psi I_{HOSPITAL,1}(t,a,v_4,1,0)$$

$$\frac{dI_{HOSPITAL,0}(t,a,v_5,0,1)}{dt} = (1-\delta(H))(1-\mu(a))(1-\phi_2(a))2\gamma_2 I_{CASE,1}(t,a,v_5) - 2\gamma_{3,1} I_{HOSPITAL,0}(t,a,v_5,0,1) + 2\psi I_{HOSPITAL,0}(t,a,v_4,0,1)$$

$$\frac{dI_{HOSPITAL,1}(t,a,v_5,0,1)}{dt} = 2\gamma_{3,1} I_{HOSPITAL,0}(t,a,v_5,0,1) - 2\gamma_{3,1} I_{HOSPITAL,1}(t,a,v_5,0,1) + 2\psi I_{HOSPITAL,1}(t,a,v_4,0,1)$$

$$\frac{dI_{HOSPITAL,0}(t,a,v_5,1,1)}{dt} = \delta(H)(1-\mu(a))(1-\phi_2(a))2\gamma_2 I_{CASE,1}(t,a,v_5) - 2\gamma_{3,1} I_{HOSPITAL,0}(t,a,v_5,1,1) + 2\psi I_{HOSPITAL,0}(t,a,v_4,1,1)$$

$$\frac{dI_{HOSPITAL,1}(t,a,v_5,1,1)}{dt} = 2\gamma_{3,1} I_{HOSPITAL,0}(t,a,v_5,1,1) - 2\gamma_{3,1} I_{HOSPITAL,1}(t,a,v_5,1,1) + 2\psi I_{HOSPITAL,1}(t,a,v_4,1,1)$$

$$\frac{dI_{ICU,0}(t,a,v_5,0,0)}{dt} = (1-\delta(ICU))\mu(a)\phi_2(a)2\gamma_2 I_{CASE,1}(t,a,v_5) - 2\gamma_{4,0} I_{ICU,0}(t,a,v_5,0,0) + 2\psi I_{ICU,0}(t,a,v_4,0,0)$$

$$\frac{dI_{ICU,1}(t,a,v_5,0,0)}{dt} = 2\gamma_{4,0} I_{ICU,0}(t,a,v_5,0,0) - 2\gamma_{4,0} I_{ICU,1}(t,a,v_5,0,0) + 2\psi I_{ICU,1}(t,a,v_4,0,0)$$

$$\frac{dI_{ICU,0}(t,a,v_5,1,0)}{dt} = \delta(ICU)\mu(a)\phi_2(a)2\gamma_2 I_{CASE,1}(t,a,v_5) - 2\gamma_{4,0} I_{ICU,0}(t,a,v_5,1,0) + 2\psi I_{ICU,0}(t,a,v_4,1,0)$$

$$\frac{dI_{ICU,1}(t,a,v_5,1,0)}{dt} = 2\gamma_{4,0} I_{ICU,0}(t,a,v_5,1,0) - 2\gamma_{4,0} I_{ICU,1}(t,a,v_5,1,0) + 2\psi I_{ICU,1}(t,a,v_4,1,0)$$

$$\frac{dI_{ICU,0}(t,a,v_5,0,1)}{dt} = (1-\delta(ICU))(1-\mu(a))\phi_2(a)2\gamma_2 I_{CASE,1}(t,v_5,a) - 2\gamma_{4,1} I_{ICU,0}(t,a,v_5,0,1) + 2\psi I_{ICU,0}(t,a,v_4,0,1)$$

$$\frac{dI_{ICU,1}(t,a,v_5,0,1)}{dt} = 2\gamma_{4,1} I_{ICU,0}(t,a,v_5,0,1) - 2\gamma_{4,1} I_{ICU,1}(t,a,v_5,0,1) + 2\psi I_{ICU,1}(t,a,v_4,0,1)$$

$$\frac{dI_{ICU,0}(t,a,v_5,1,1)}{dt} = \delta(ICU)(1-\mu(a))\phi_2(a)2\gamma_2 I_{CASE,1}(t,a,v_5) - 2\gamma_{4,1} I_{ICU,0}(t,a,v_5,1,1) + 2\psi I_{ICU,0}(t,a,v_4,1,1)$$

$$\frac{dI_{ICU,1}(t,a,v_5,1,1)}{dt} = 2\gamma_{4,1} I_{ICU,0}(t,a,v_5,1,1) - 2\gamma_{4,1} I_{ICU,1}(t,a,v_5,1,1) + 2\psi I_{ICU,1}(t,a,v_4,1,1)$$

$$\frac{dI_{REC,0}(t,a,v_5)}{dt} = 2\gamma_{4,1} I_{ICU,1}(t,a,v_5,0,1) + 2\gamma_{4,1} I_{ICU,1}(t,a,v_5,1,1) - 2\gamma_5 I_{REC,0}(t,a,v_5) + 2\psi I_{REC,0}(t,a,v_4)$$

$$\frac{dI_{REC,1}(t,a,v_5)}{dt} = 2\gamma_5 I_{REC,0}(t,a,v_5) - 2\gamma_5 I_{REC,1}(t,a,v_5) + 2\psi I_{REC,1}(t,a,v_4)$$

$$\frac{dR_1(t,a,v_5)}{dt} = \gamma_1 I_{MILD}(t,a,v_5) + 2\gamma_{3,1} I_{HOSPITAL,1}(t,a,v_5,0,1) + 2\gamma_{3,1} I_{HOSPITAL,1}(t,a,v_5,1,1) + 2\gamma_5 I_{REC,1}(t,a,v_5) + 2\gamma_{4,1} I_{ICU,1}(t,a,v_5,0,1) + 2\gamma_{4,1} I_{ICU,1}(t,a,v_5,1,1) - 2\rho R_1(t,a,v_5) + 2\psi R_1(t,a,v_4)$$

$$\frac{dR_2(t,a,v_5)}{dt} = 2\rho R_1(t,a,v_5) - 2\rho R_2(t,a,v_5) + 2\psi R_2(t,a,v_4)$$

$$\frac{dD(t,a,v_5)}{dt} = 2\gamma_{3,0} I_{HOSPITAL,1}(t,a,v_5,0,0) + 2\gamma_{3,0} I_{HOSPITAL,1}(t,a,v_5,1,0) + 2\gamma_{4,0} I_{ICU,1}(t,a,v_5,0,0) + 2\gamma_{4,0} I_{ICU,1}(t,a,v_5,1,0)$$

1.4 Model validation

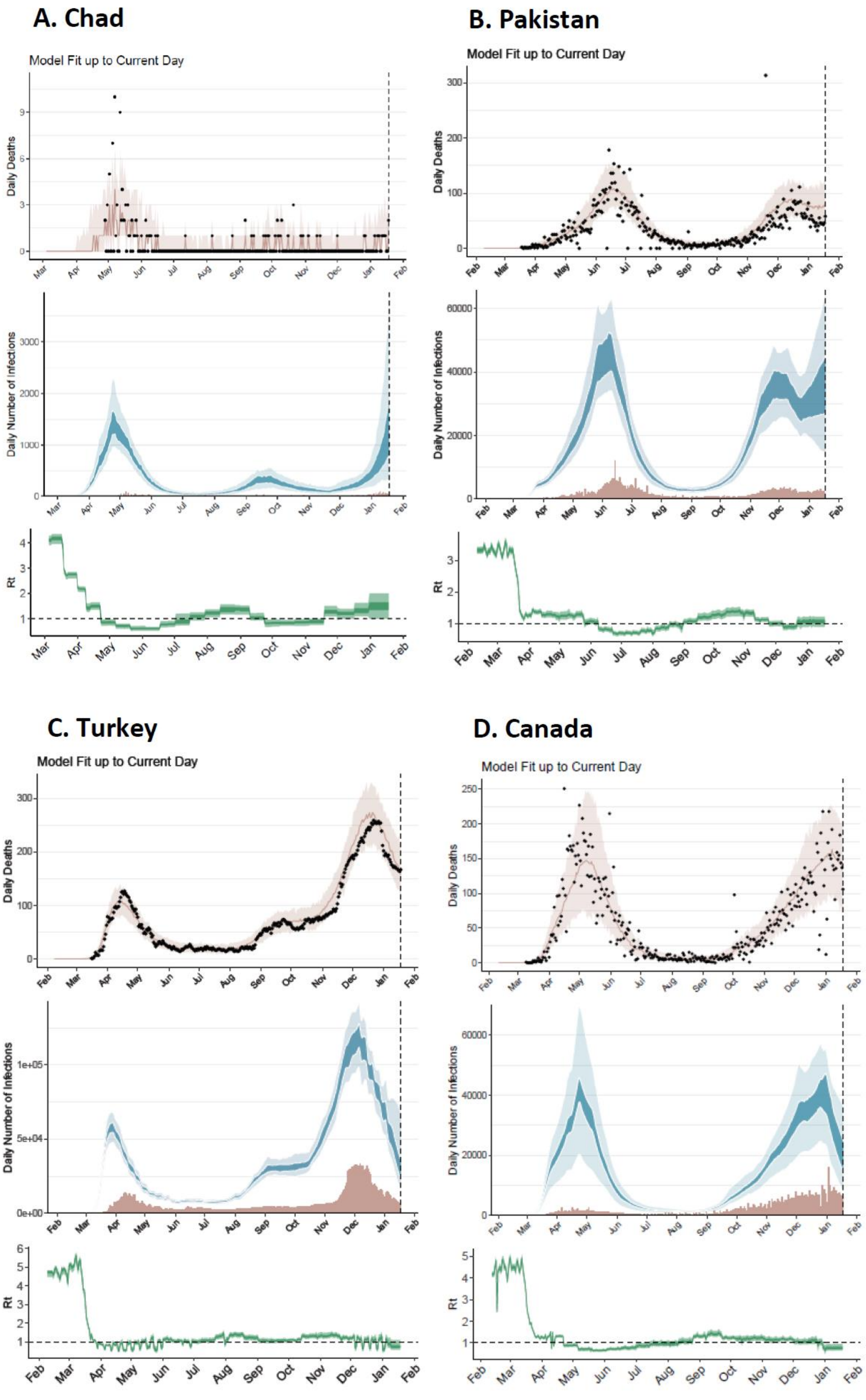

Figure S2: Country-specific calibration of the SARS-CoV-2 transmission model (as at 18 January 2021)*[37]*. *Four example countries are shown: Chad (low-income); Pakistan (lower-middle-income); Turkey (upper-middle-income); and Canada (high-income). Each upper panel shows the modelled daily deaths calibrated to the daily deaths as reported by the COVID-19 Data Repository by the Center for Systems Science and Engineering (CSSE) at Johns Hopkins University*[38]*. For countries not included in that repository, data are obtained from Worldometer*[39]*. Each middle panel shows the daily number of infections estimated by fitting to the current total of deaths, with reported cases shown in red and the model-estimated infections shown in blue (dark blue 50% interquartile range, light blue 95% quantile). The dashed vertical lines show the current day. Each lower panel shows the estimated reproduction number over time,* R_t.

1.5 Health system assumptions

For each income setting, we consider two possibilities for health system capacity. The first is that all health systems are unconstrained – and hence that a constant (age-dependent) proportion of infections are hospitalised and receive appropriate care, regardless of the size of the epidemic. This results in population-level IFRs that are highest in HIC settings and lowest in LIC settings given the different demographics of these populations[1]. Second, we more realistically assume that health systems will be constrained to varying degrees. Here we follow the assumptions and parameters in Walker et al.[1] in which LIC and LMIC settings have limited hospital capacity (estimated using World Bank data) and that once exceeded, those requiring hospitalisation but who do not receive it experience worse outcomes. In contrast, in UMIC and HIC settings, while existing hospital capacity may also be exceeded, we assume that surge capacity is implemented to fill this gap. In LIC settings we additionally assume that outcomes for hospitalised cases are worse than in other settings. This results in slightly higher population-level IFRs in LIC and LMIC compared to UMIC and HIC settings.

1.6 Timing of transmission and vaccination

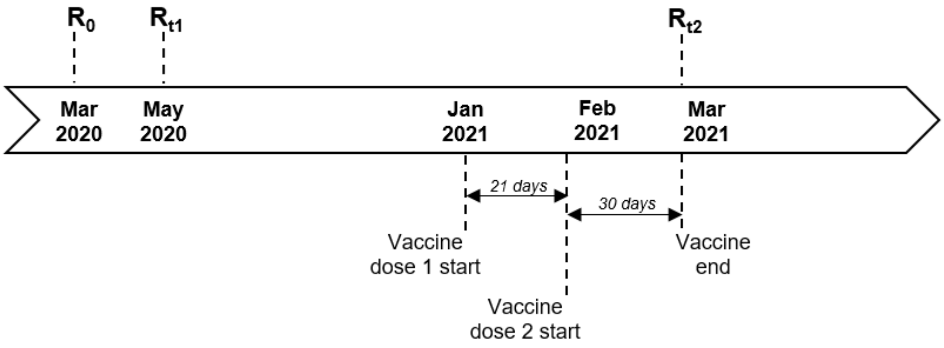

Figure S3: Schematic illustration of the timing of changes in levels of transmission and the introduction of vaccination.

1.7 Additional methods for vaccine age-targeting

For the optimisation of vaccine allocation by income setting and age target, we run the simulation for the vaccine distributed to combinations of 5-year age groups from 0–4 years up to 75–79 years and 80+ years. Since it is unlikely that multiple non-sequential 5-year age groups would be selected in any vaccine programme (for example, vaccinating only the 20–24-, 40–44- and 60–64-year-olds is not programmatically likely), and rather than simulating impact for every possible combination of age groups that could be targeted, we construct the parameter space such that the vaccine could be targeted to up to two distinct contiguous groups, or rather up two non-overlapping age groups that are each comprised of any number of consecutive 5-year groups. This would allow, for example, the elderly and children to be selected. The age group combinations are depicted in Figure S4.

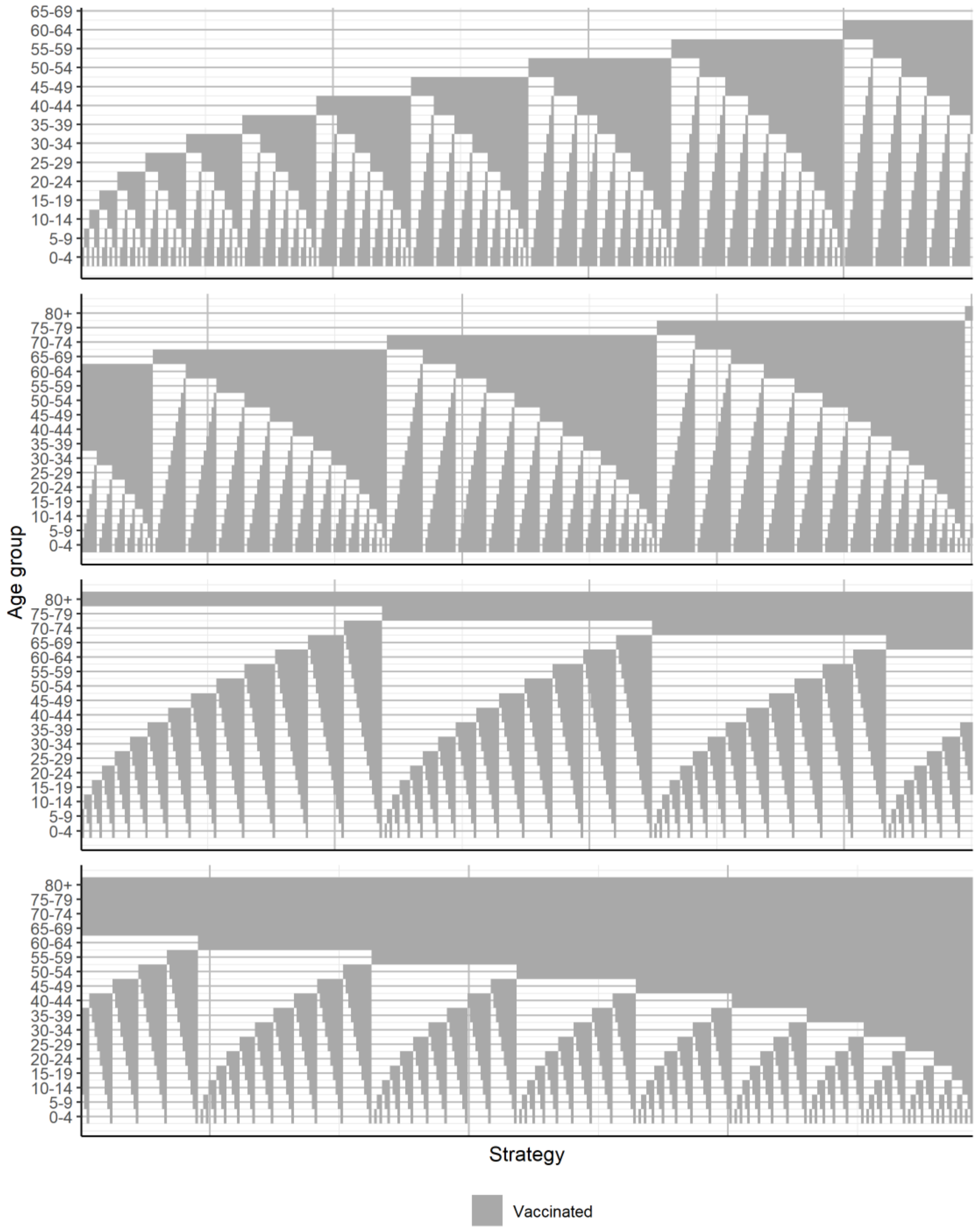

Figure S4: Illustration of 5-year age group combinations. *Each column represents a unique vaccine age-targeting strategy indicating which age groups (rows) are vaccinated (grey shaded regions) or unvaccinated (unshaded regions) for that strategy.*

2 Extended results

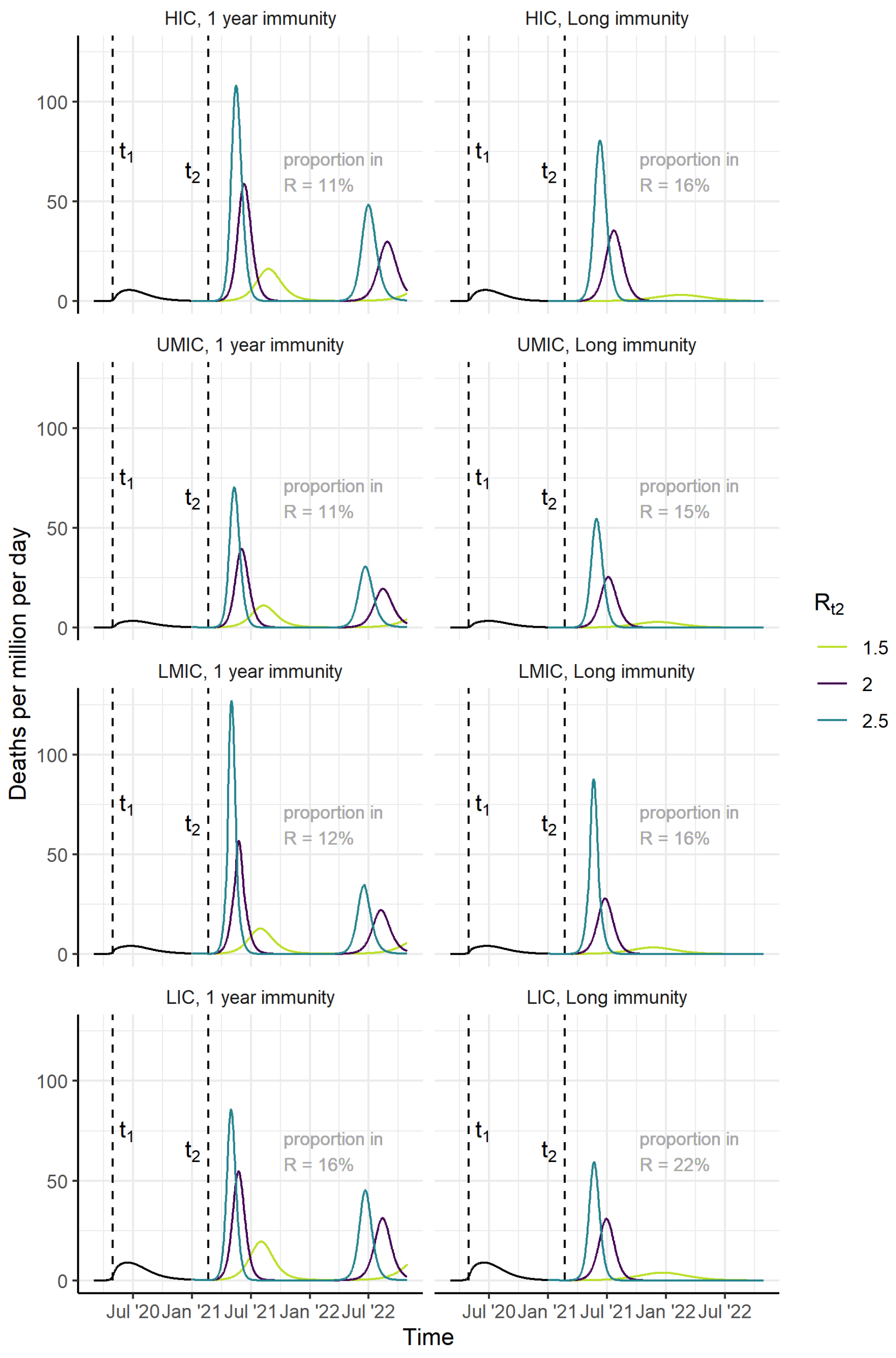

Figure S5: Scenarios for the course of the epidemic from 2020–2022, for high-income, upper-middle-income, lower-middle-income and low-income country settings (HIC, UMIC, LMIC and LIC respectively). *(A, C, E, G) assuming an average duration of naturally-acquired immunity of one year; (B, D, F, H) assuming long-term immunity. We assume R_0=2.5 up to time t_1 (May 2020) and that R_{t1} drops to 1.1 between time t_1 and t_2 (end-February 2021). From time t_2 onwards, we consider three counterfactual scenarios, R_{t2}=1.5, 2.0 and 2.5, as shown in light green, purple and turquoise respectively. The grey annotated text indicates the proportion of the population in the recovered class at the time of vaccine introduction (dose 2).*

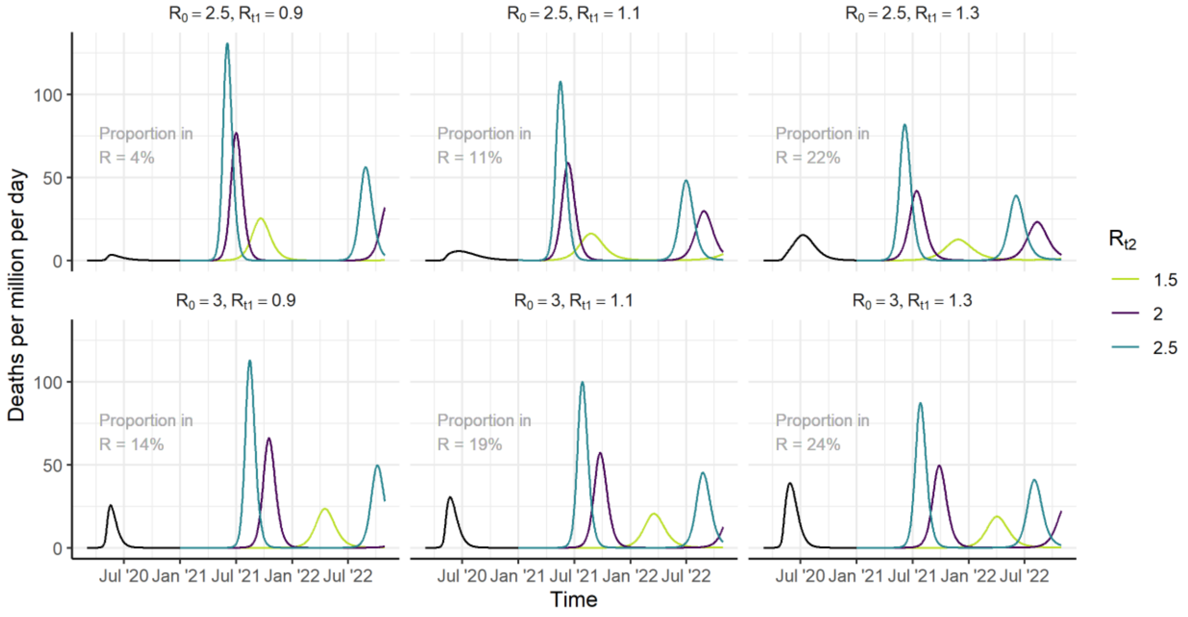

Figure S6: Scenarios for the course of the epidemic from 2020–2022 (counterfactual scenarios). *Epidemic trajectories are shown for a high-income country setting, in the absence of a vaccine, for a range of values of* R_0 *(rows),* R_{t1} *(columns), and* R_{t2} *(coloured lines). The grey annotated text indicates the proportion of the population in the recovered class at the time of vaccine introduction (dose 2). Immunity following infection is assumed to persist for one year.*

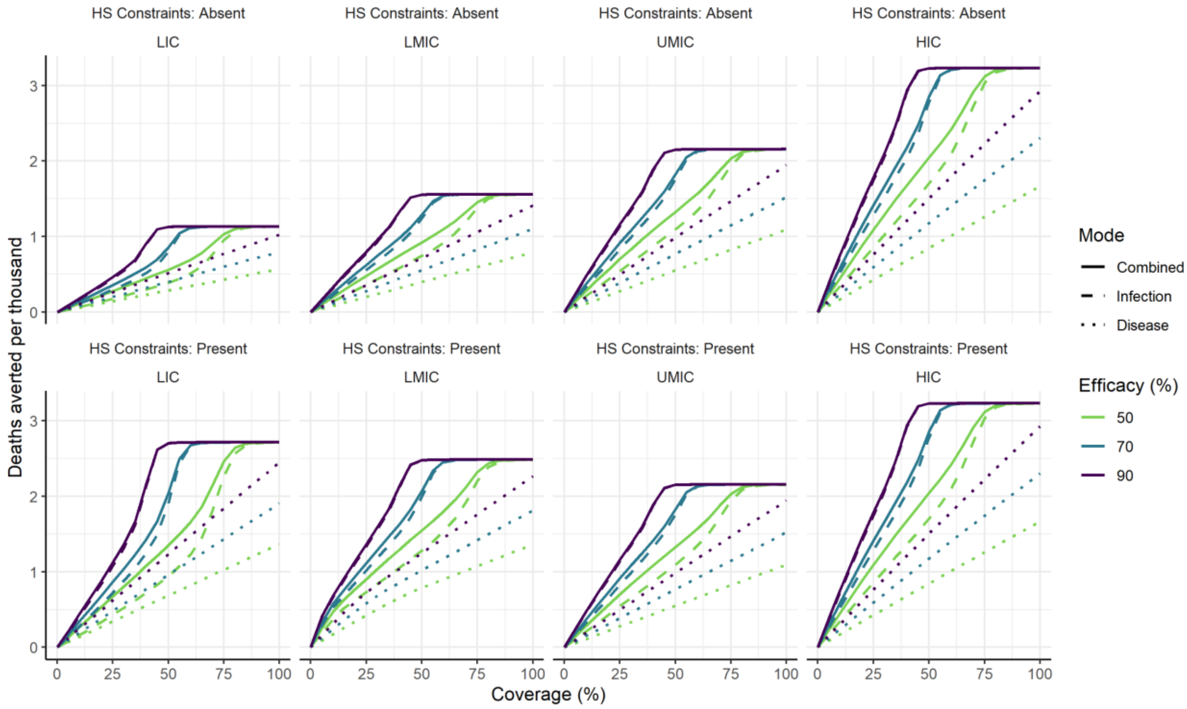

Figure S7: Vaccine efficacy and herd immunity by income setting. *Projected total deaths averted per thousand population in 2021 under the default vaccine scenarios shown in Table 1, for the four income settings (columns), and with health system constraints either absent or present (rows). The colours show different vaccine efficacy assumptions (from 50% to 90%). Solid lines represent impact for a vaccine that is efficacious against infection, with additional efficacy against severe disease. Dashed lines represent a vaccine that is efficacious against infection only, and dotted lines represent a vaccine that prevents severe disease (and hence death) but does not reduce infection or onwards transmission (Table S3).*

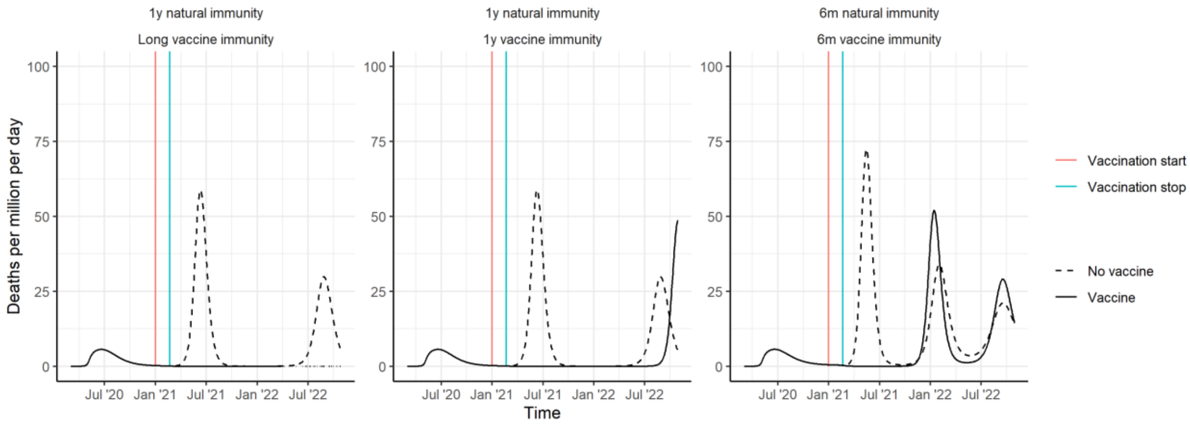

Figure S8: Epidemic trajectories and impact of immunity. *Epidemic scenarios are shown for the period 2020–2022, both in the absence of a vaccine (dashed black lines) and following vaccine introduction (solid black lines). Vaccine implementation is indicated by the red and blue vertical lines. The left panel represents the scenario where vaccine-derived immunity is long-term, and naturally-acquired immunity is one year. The middle panel represents where both vaccine- and naturally-acquired immunity are one year, while the right panel shows trajectories where both durations are six months. Trajectories are shown for a high-income country setting, and assuming the default transmission and vaccine parameters as in Table 1.*

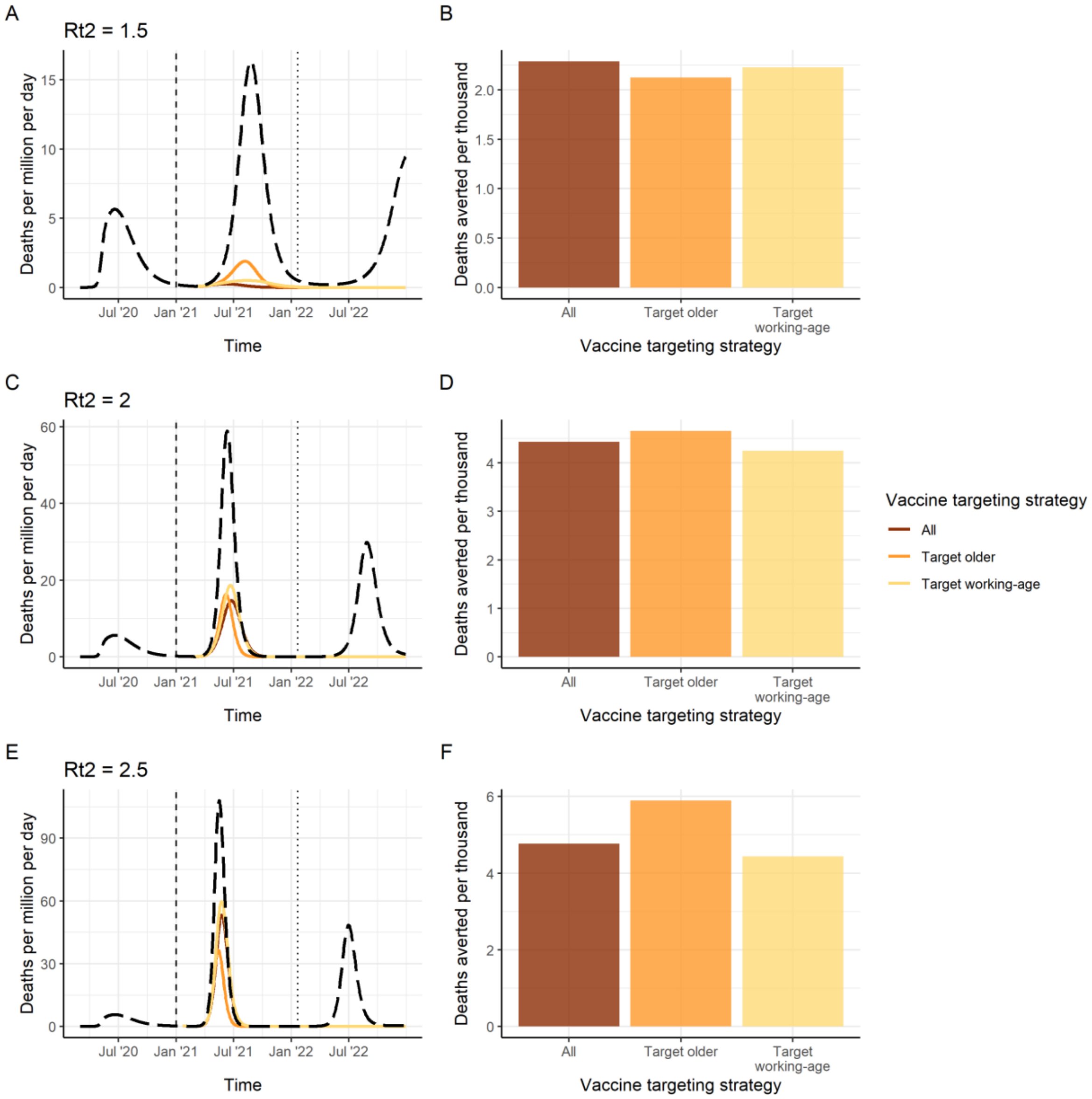

Figure S9: Vaccine impact over a one-year vaccination period. *Here, NPIs are assumed to be lifted when the vaccine is introduced, rather than after the target population is vaccinated, and the target population is vaccinated over a period of one year. Vaccination start is shown by the vertical dashed line, and vaccination stop by the vertical dotted line. Three different values of* R_{t2} *are shown:* $R_{t2} = 1.5$ *(A,B);* $R_{t2} = 2.0$ *(C,D; the default value) and* $R_{t2} = 2.5$ *(E,F). The left-hand-side panels show the deaths per million per day, for the counterfactual scenario (long-dashed black line), and three vaccine targeting strategies (coloured lined). The right-hand-side panels show the total deaths averted in 2021–2022 per thousand population for three vaccine targeting strategies, where vaccination takes place at a constant rate over the course of 2021. "All": all age groups vaccinated simultaneously. "Target older": the 80+ group is vaccinated first, then additional groups (75–79, 70–74 and so on) are consecutively vaccinated. "Target working-age": the 15–64-year-old group is vaccinated first, and then the older group, and then children.*

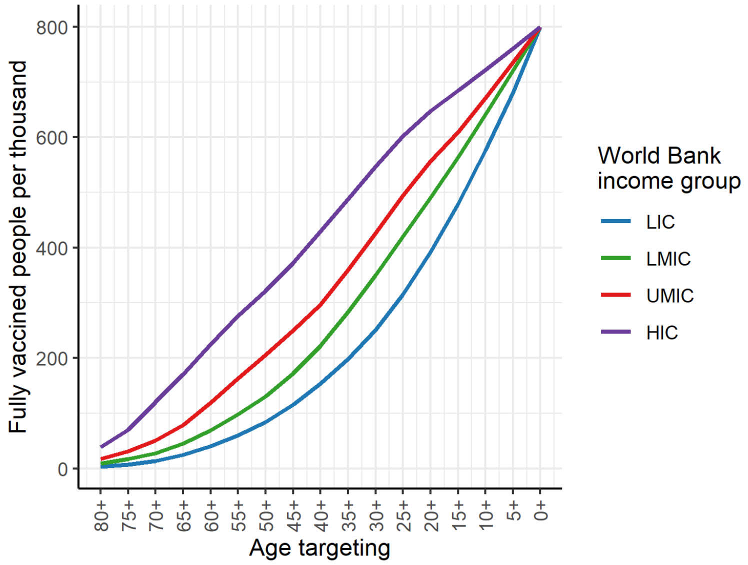

Figure S10: Resources required when vaccine introduction is targeted by age. *Here, the highest risk age groups are prioritised. Resources required is presented as a proportion of the total population.*

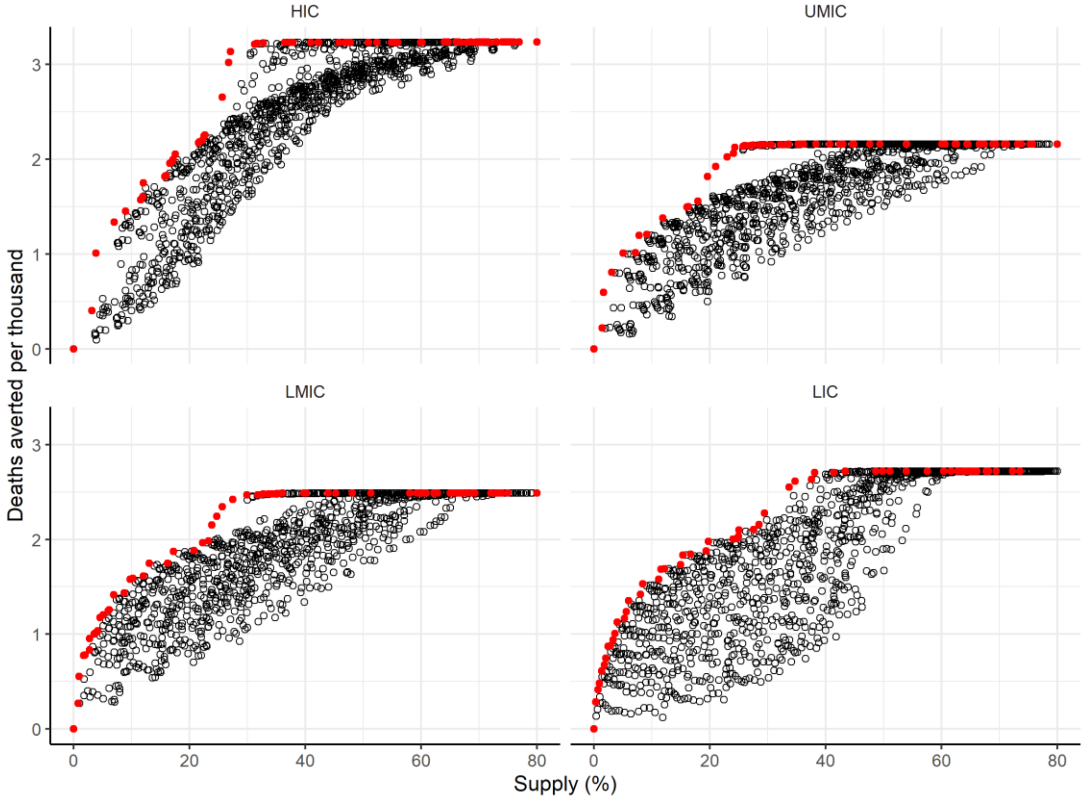

Figure S11: Efficiency frontier for the age targeting of a vaccine within each income setting. *The black circles each represent a unique age targeting strategy, for each income setting, for increasing availability of doses on the x-axis, versus impact in terms of deaths averted per thousand population on the y-axis. The red points represent the most efficient (non-dominated) age-targeting strategies, or the maximum deaths averted as the vaccine supply is increased. These red points correspond to the age targeting strategies shown in Figure 4A, C, E and G, and the optimal allocation strategy in Figure 4B, D, F and H.*

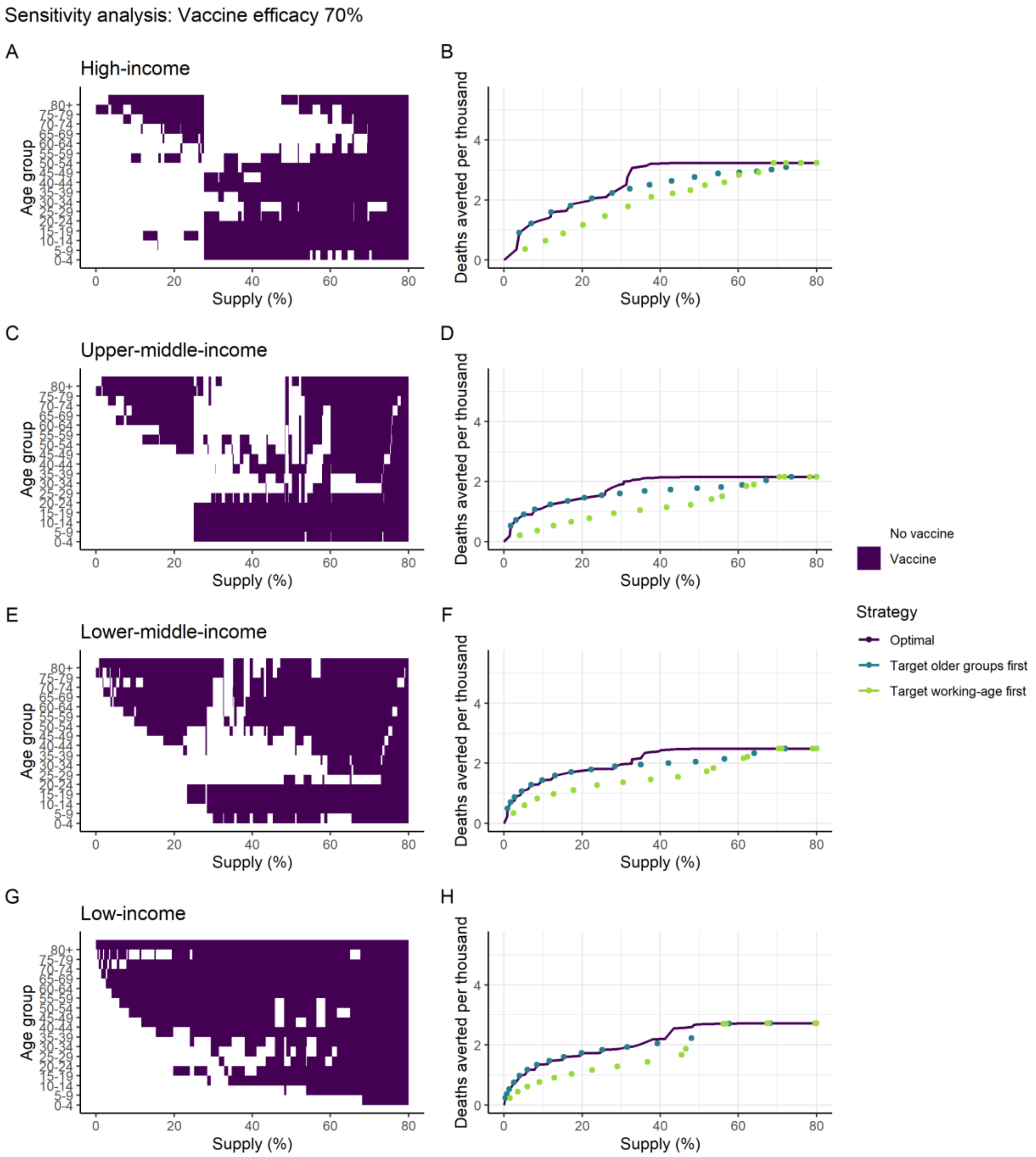

Figure S12: Sensitivity analysis of targeting of vaccine introduction within each income setting; lower vaccine efficacy (70%). *These panels illustrate the most efficient allocation under different dose constraints, where the supply is defined as the proportion of the population able to access two doses. Panels A, C, E and G show the age groups allocated under each supply level, where the purple shaded regions indicate the age groups allocated the vaccine. Panels B, D, F and H show the efficiency frontiers expressed as deaths averted per thousand population as a function of vaccine supply. The optimal strategies from the left-hand panels are shown in purple. The turquoise shows the strategy that prioritises the older at-risk age: 80+ for the lowest coverage level, and sequentially including additional age groups (75–79, 70–74 and so on) as additional doses are available. The green strategy prioritises the working age population first (beginning with the 60–64 age group and sequentially adding younger groups), then vaccinates the elderly and children as doses become available.*

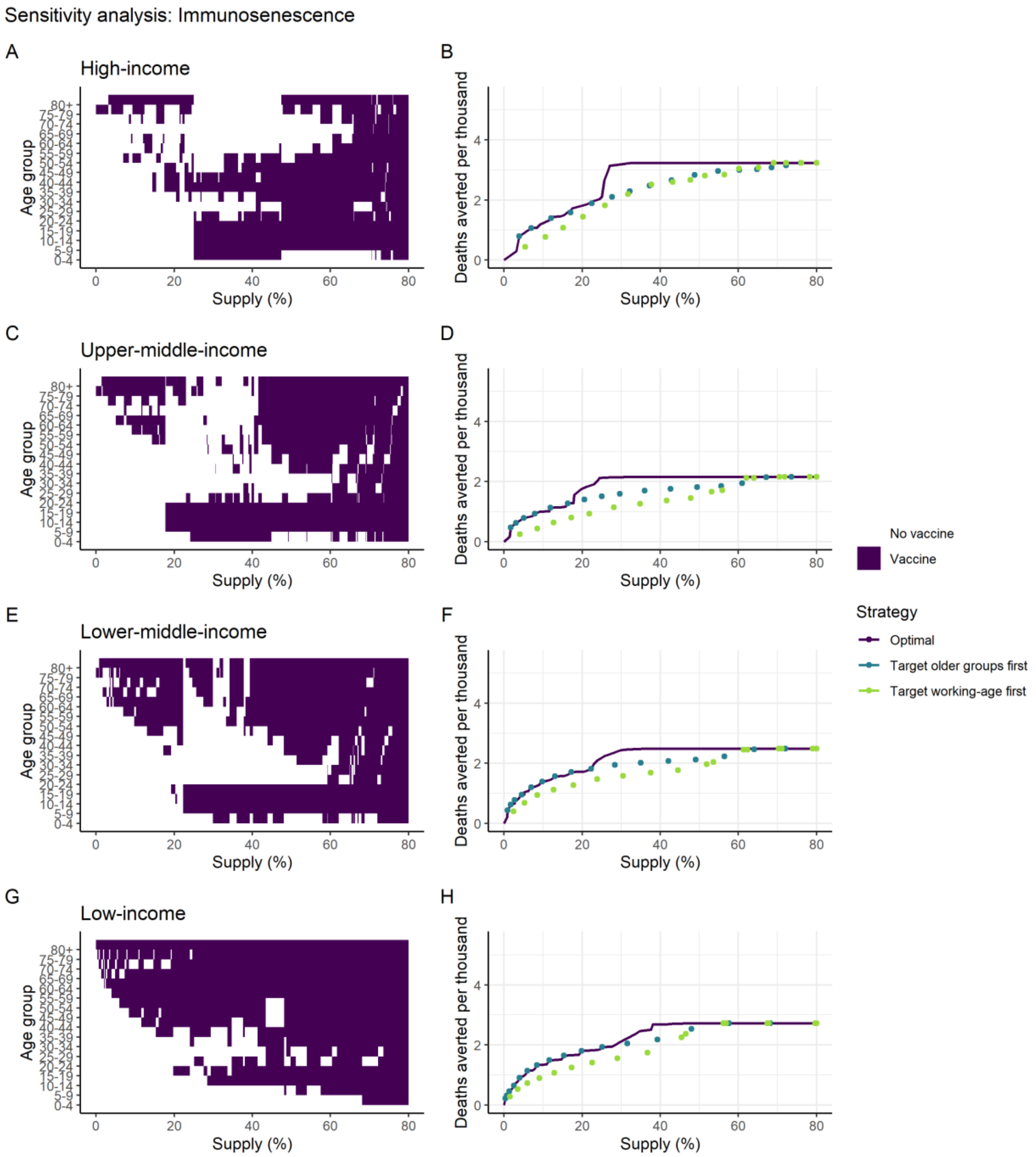

Figure S13: Sensitivity analysis of targeting of vaccine introduction within each income setting; reduced vaccine impact in 65+ age group (immunosenescence). *Vaccine efficacy was reduced in the 65+ age group to 35%. These panels illustrate the most efficient allocation under different dose constraints, where the supply is defined as the proportion of the population able to access two doses. Panels A, C, E and G show the age groups allocated under each supply level, where the purple shaded regions indicate the age groups allocated the vaccine. Panels B, D, F and H show the efficiency frontiers expressed as deaths averted per thousand population as a function of vaccine supply. The optimal strategies from the left-hand panels are shown in purple. The turquoise shows the strategy that prioritises the older at-risk age: 80+ for the lowest coverage level, and sequentially including additional age groups (75–79, 70–74 and so on) as additional doses are available. The green strategy prioritises the working age population first (beginning with the 60–64 age group and sequentially adding younger groups), then vaccinates the elderly and children as doses become available.*

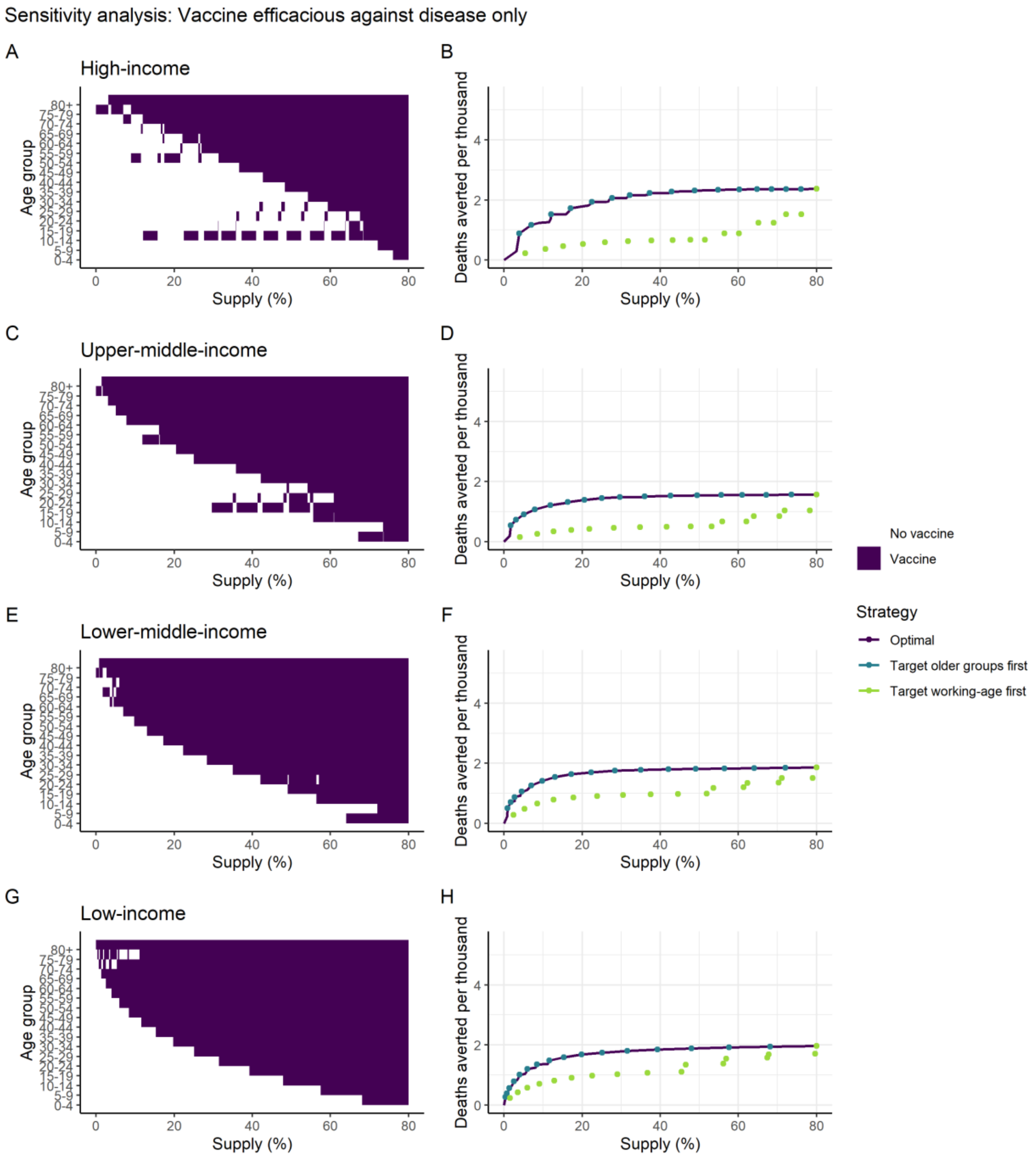

Figure S14: Sensitivity analysis of targeting of vaccine introduction within each income setting; vaccine efficacious against disease only. *These panels illustrate the most efficient allocation under different dose constraints, where the supply is defined as the proportion of the population able to access two doses. Panels A, C, E and G show the age groups allocated under each supply level, where the purple shaded regions indicate the age groups allocated the vaccine. Panels B, D, F and H show the efficiency frontiers expressed as deaths averted per thousand population as a function of vaccine supply. The optimal strategies from the left-hand panels are shown in purple. The turquoise shows the strategy that prioritises the older at-risk age: 80+ for the lowest coverage level, and sequentially including additional age groups (75–79, 70–74 and so on) as additional doses are available. The green strategy prioritises the working age population first (beginning with the 60–64 age group and sequentially adding younger groups), then vaccinates the elderly and children as doses become available.*

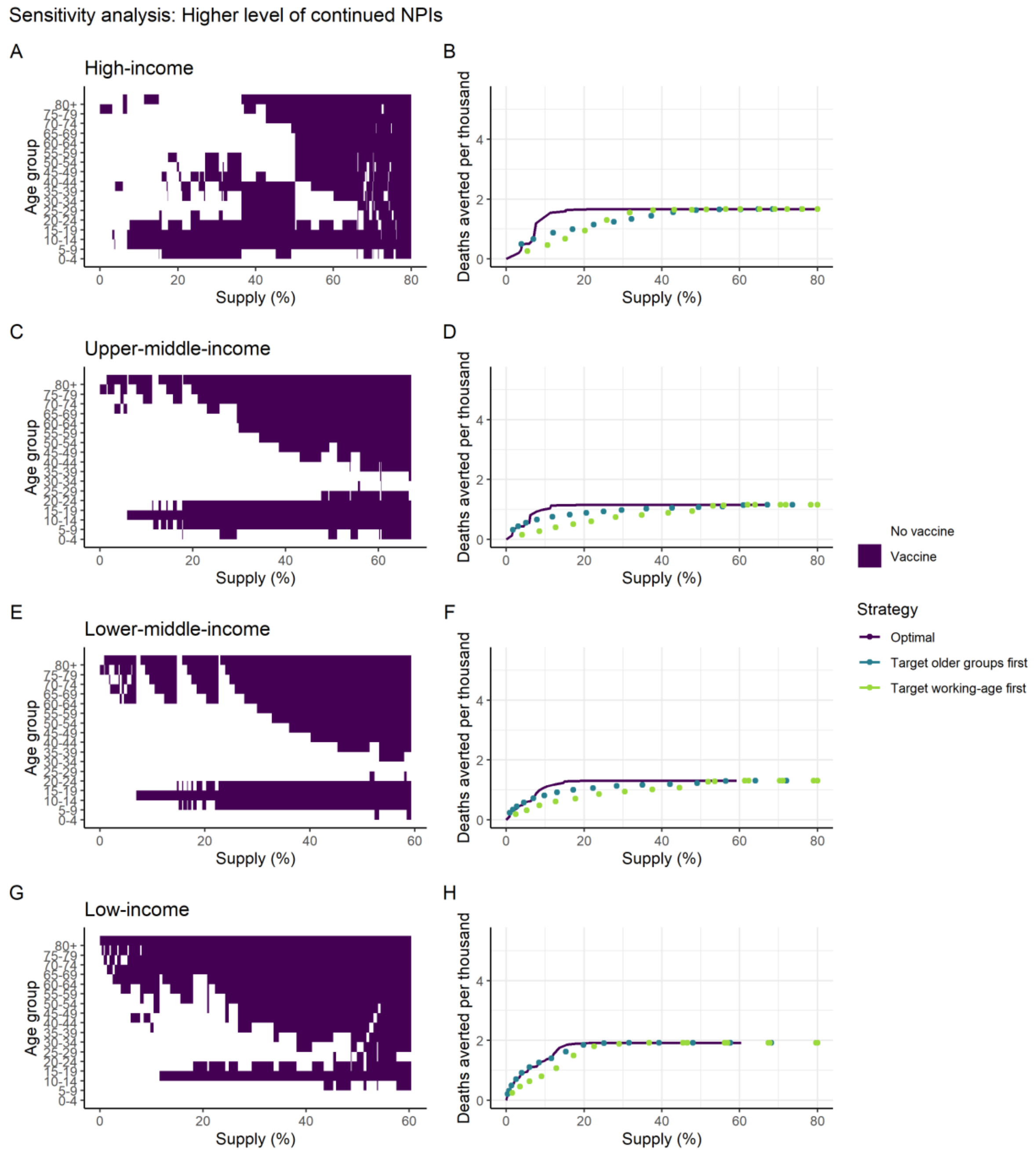

Figure S15: Sensitivity analysis of targeting of vaccine introduction within each income setting; R_{t2}***=1.5.*** *These panels illustrate the most efficient allocation under different dose constraints, where the supply is defined as the proportion of the population able to access two doses. Panels A, C, E and G show the age groups allocated under each supply level, where the purple shaded regions indicate the age groups allocated the vaccine. Panels B, D, F and H show the efficiency frontiers expressed as deaths averted per thousand population as a function of vaccine supply. The optimal strategies from the left-hand panels are shown in purple. The turquoise shows the strategy that prioritises the older at-risk age: 80+ for the lowest coverage level, and sequentially including additional age groups (75–79, 70–74 and so on) as additional doses are available. The green strategy prioritises the working age population first (beginning with the 60–64 age group and sequentially adding younger groups), then vaccinates the elderly and children as doses become available.*

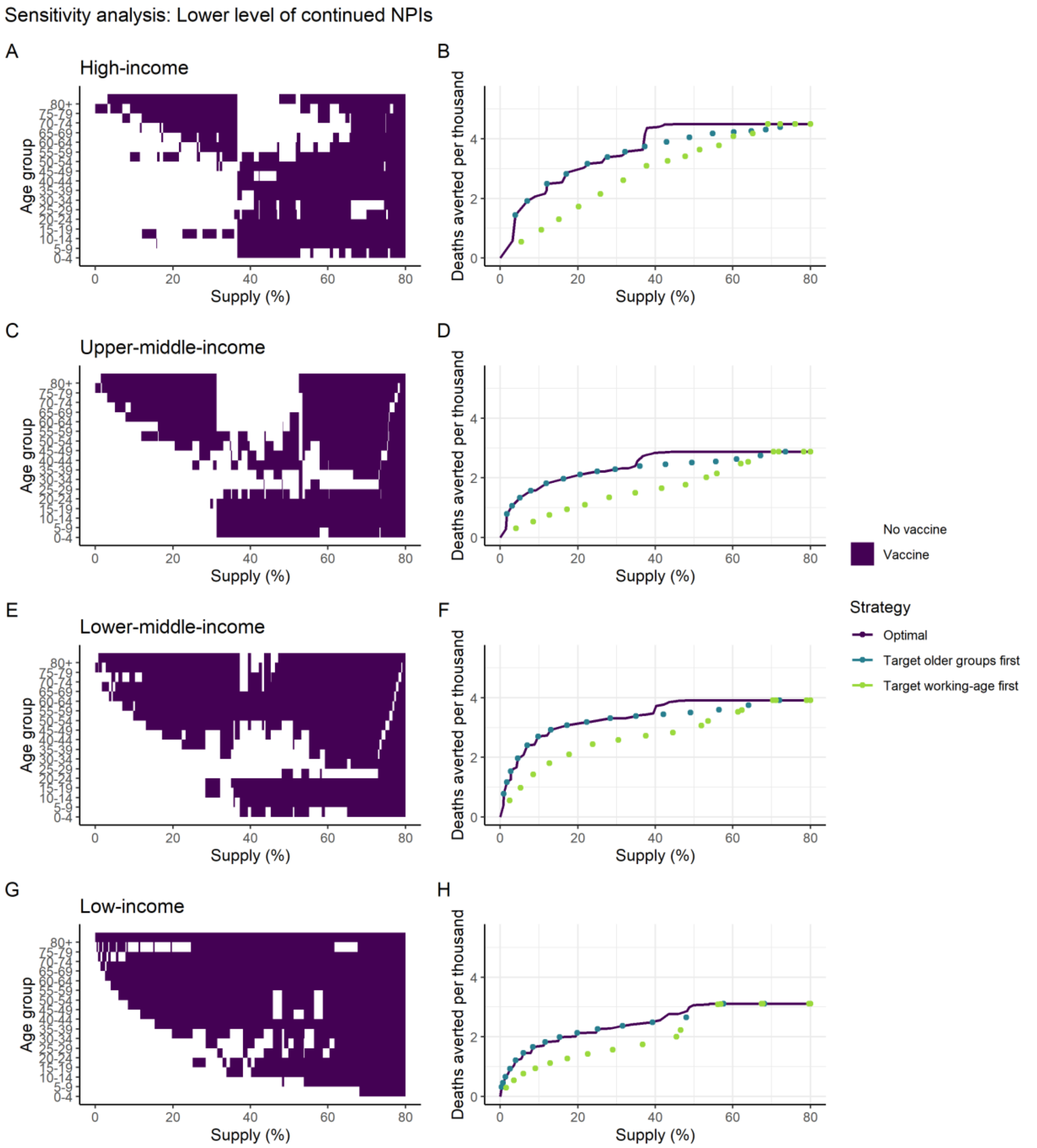

Figure S16: Sensitivity analysis of targeting of vaccine introduction within each income setting; R_{t2}=2.5. *These panels illustrate the most efficient allocation under different dose constraints, where the supply is defined as the proportion of the population able to access two doses. Panels A, C, E and G show the age groups allocated under each supply level, where the purple shaded regions indicate the age groups allocated the vaccine. Panels B, D, F and H show the efficiency frontiers expressed as deaths averted per thousand population as a function of vaccine supply. The optimal strategies from the left-hand panels are shown in purple. The turquoise shows the strategy that prioritises the older at-risk age: 80+ for the lowest coverage level, and sequentially including additional age groups (75–79, 70–74 and so on) as additional doses are available. The green strategy prioritises the working age population first (beginning with the 60–64 age group and sequentially adding younger groups), then vaccinates the elderly and children as doses become available.*

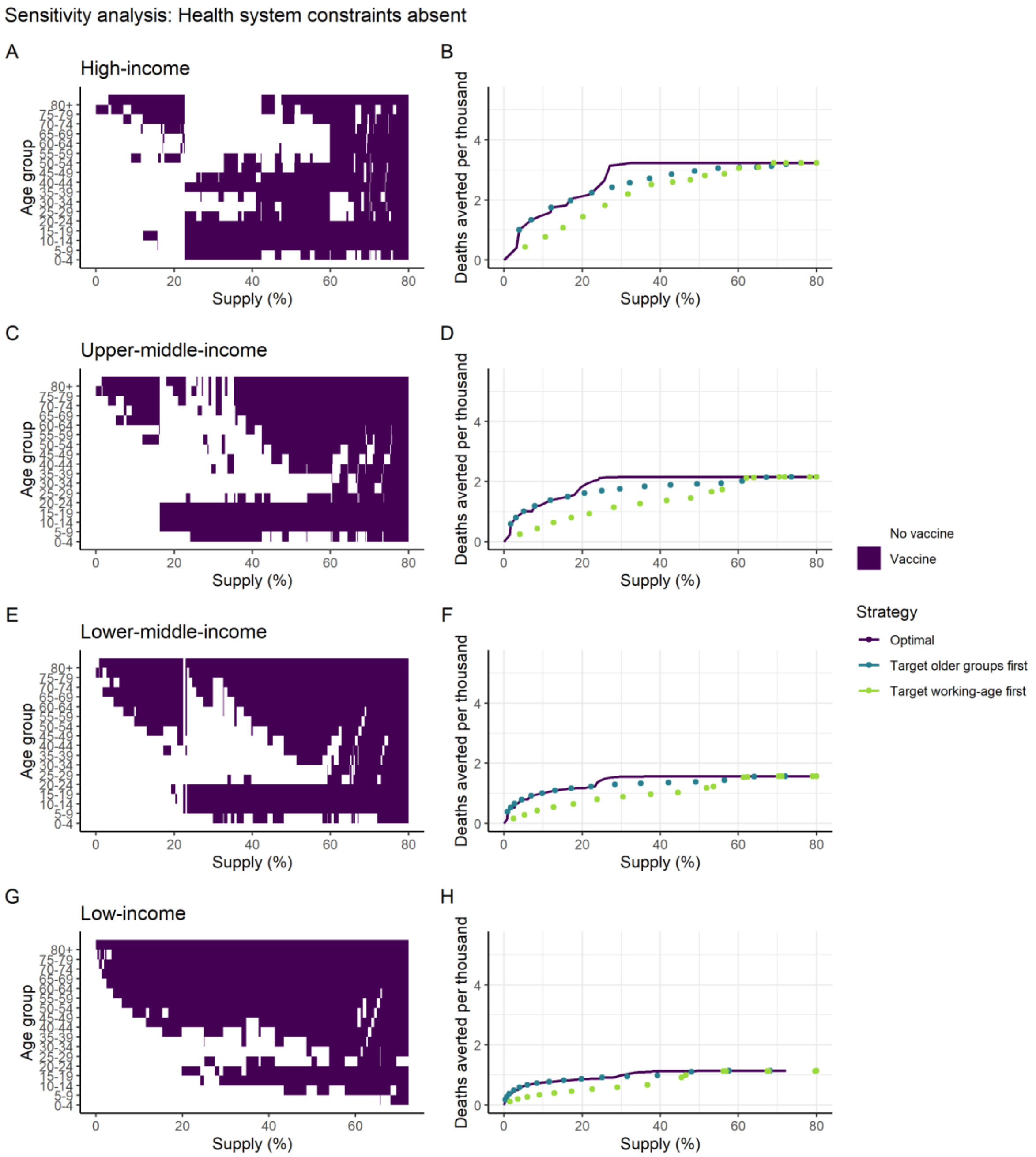

Figure S17: Sensitivity analysis of targeting of vaccine introduction within each income setting; health system constraints absent. *These panels illustrate the most efficient allocation under different dose constraints, where the supply is defined as the proportion of the population able to access two doses. Panels A, C, E and G show the age groups allocated under each supply level, where the purple shaded regions indicate the age groups allocated the vaccine. Panels B, D, F and H show the efficiency frontiers expressed as deaths averted per thousand population as a function of vaccine supply. The optimal strategies from the left-hand panels are shown in purple. The turquoise shows the strategy that prioritises the older at-risk age: 80+ for the lowest coverage level, and sequentially including additional age groups (75–79, 70–74 and so on) as additional doses are available. The green strategy prioritises the working age population first (beginning with the 60–64 age group and sequentially adding younger groups), then vaccinates the elderly and children as doses become available.*

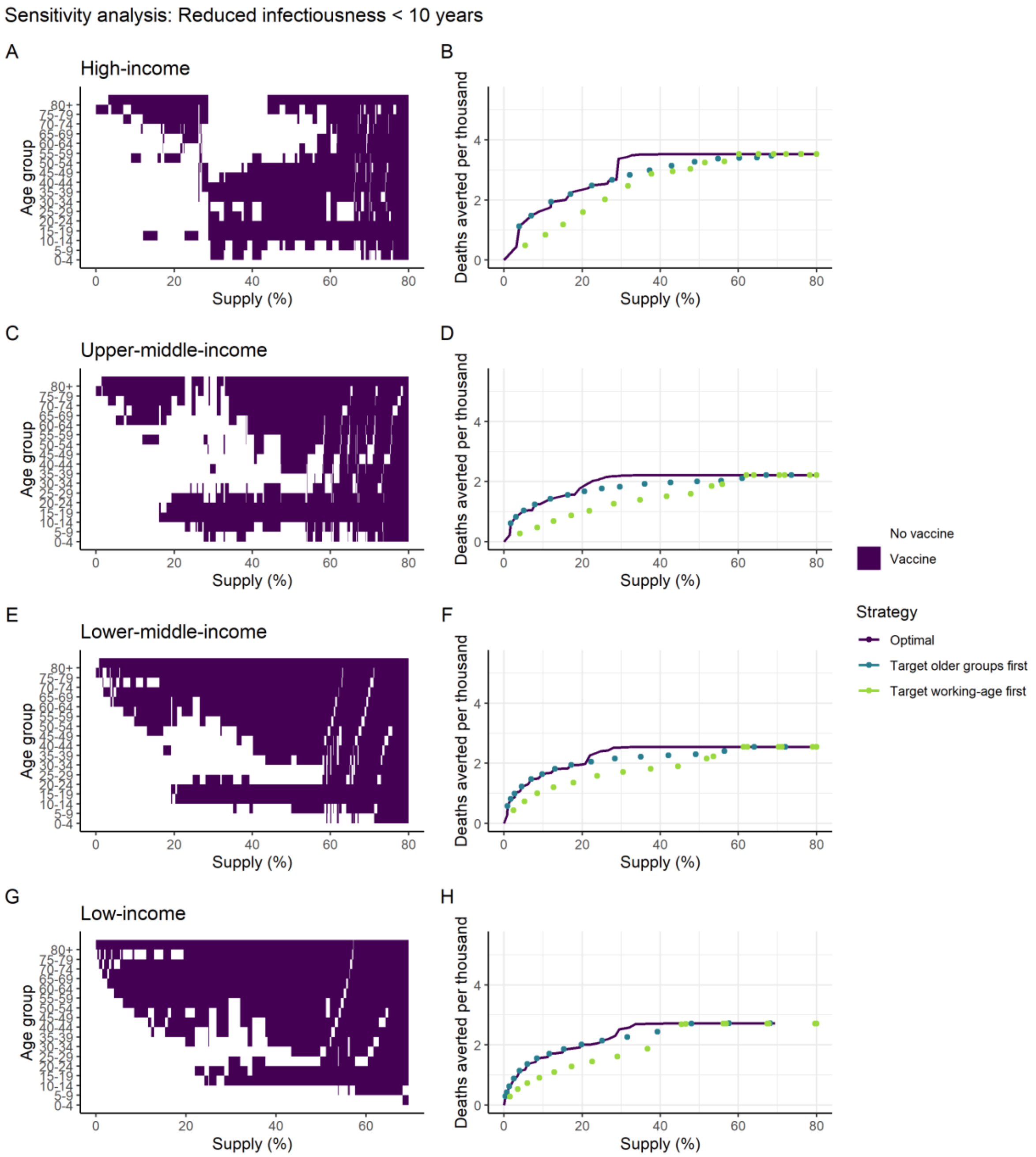

Figure S18: Sensitivity analysis of targeting of vaccine introduction within each income setting; transmission from children younger than 10 years reduced by 50%. *These panels illustrate the most efficient allocation under different dose constraints, where the supply is defined as the proportion of the population able to access two doses. Panels A, C, E and G show the age groups allocated under each supply level, where the purple shaded regions indicate the age groups allocated the vaccine. Panels B, D, F and H show the efficiency frontiers expressed as deaths averted per thousand population as a function of vaccine supply. The optimal strategies from the left-hand panels are shown in purple. The turquoise shows the strategy that prioritises the older at-risk age: 80+ for the lowest coverage level, and sequentially including additional age groups (75–79, 70–74 and so on) as additional doses are available. The green strategy prioritises the working age population first (beginning with the 60–64 age group and sequentially adding younger groups), then vaccinates the elderly and children as doses become available.*

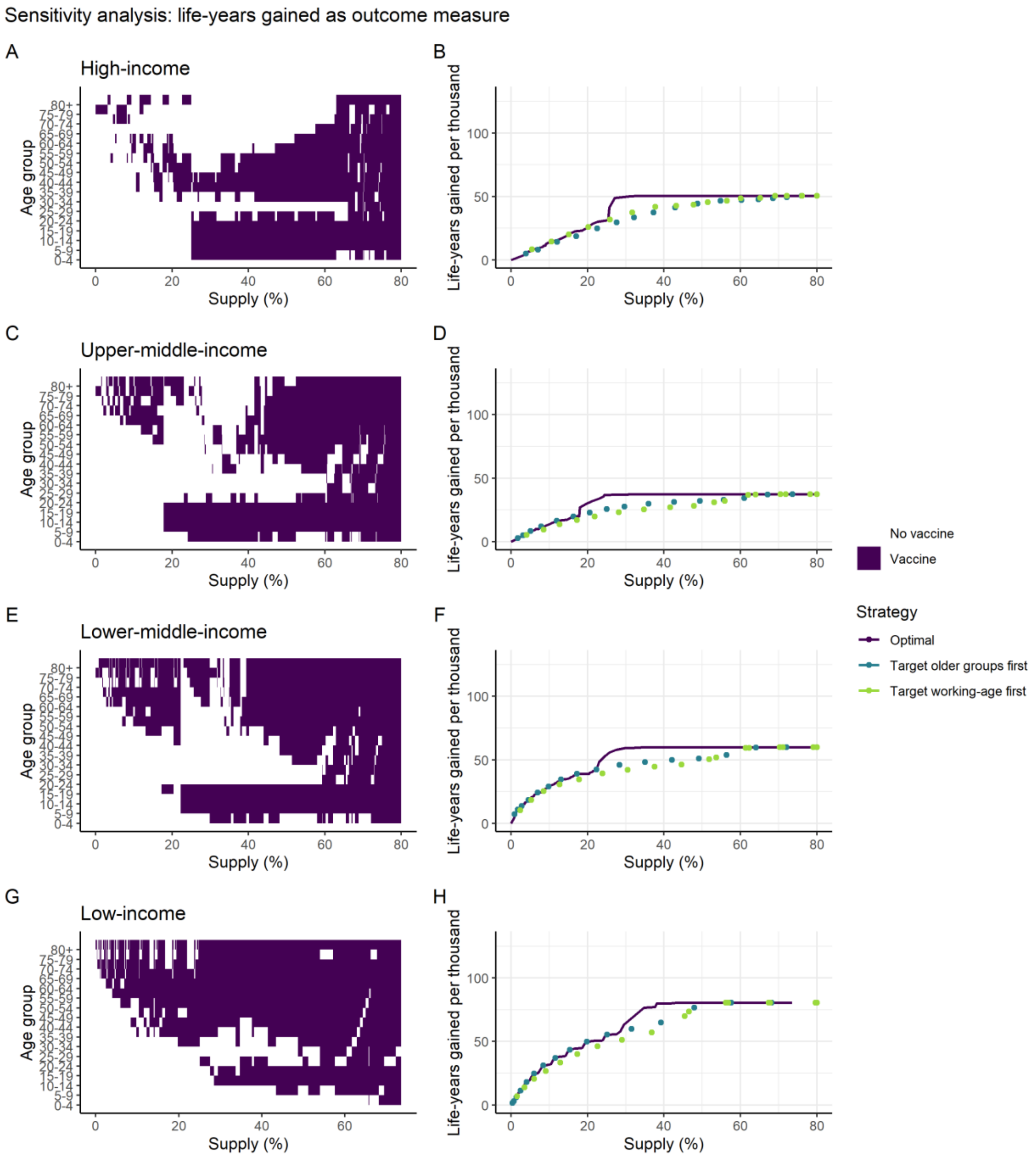

Figure S19: Sensitivity analysis of targeting of vaccine introduction within each income setting; life-years gained as outcome measure. *These panels illustrate the most efficient allocation under different dose constraints, where the supply is defined as the proportion of the population able to access two doses. Panels A, C, E and G show the age groups allocated under each supply level, where the purple shaded regions indicate the age groups allocated the vaccine. Panels B, D, F and H show the efficiency frontiers expressed as life-years gained per thousand population as a function of vaccine supply. The optimal strategies from the left-hand panels are shown in purple. The turquoise shows the strategy that prioritises the older at-risk age: 80+ for the lowest coverage level, and sequentially including additional age groups (75–79, 70–74 and so on) as additional doses are available. The green strategy prioritises the working age population first (beginning with the 60–64 age group and sequentially adding younger groups), then vaccinates the elderly and children as doses become available.*

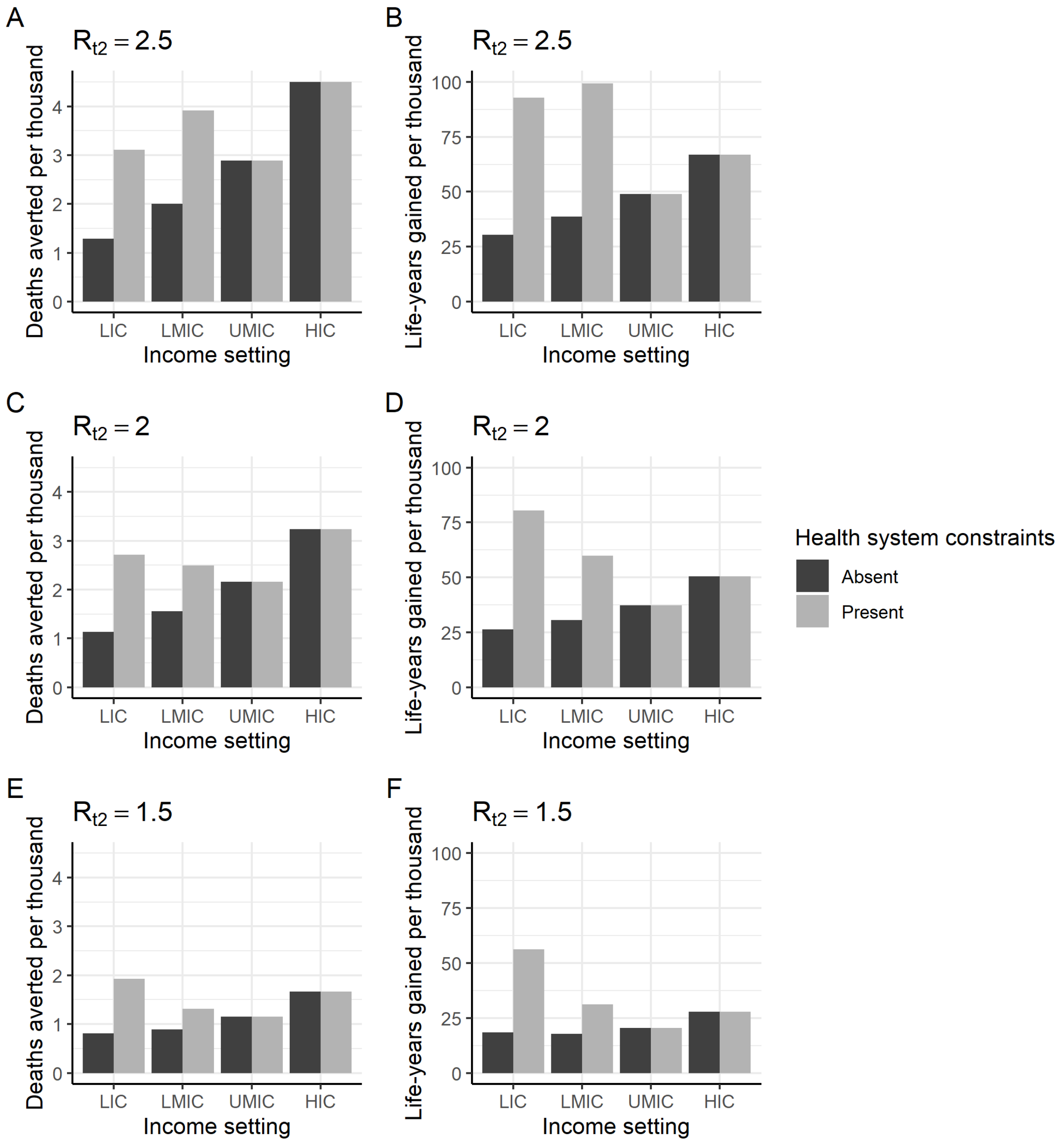

Figure S20: Vaccine impact by income setting and level of NPIs at vaccine introduction. *Deaths averted (A, C, E) and life years gained (B, D, F) per thousand population in 2021 for each income setting (x-axis), where health systems are either unconstrained (dark grey) or constrained (light grey), and for* R_{t2} *= 2.5 (upper row),* R_{t2}*=2 (default value, middle row) and* R_{t2}*=1.5 (lower row). Default vaccine parameters are in Table 1.*

Table S4: Global allocation of vaccine doses for non-optimised scenarios*. Here we assume that limited countries within each income setting are allocated doses at high (80%) coverage, rather than all countries being allocated doses at a lower level of coverage as in Table 2. The global vaccine supply is assumed to be constrained to 2 billion doses, with a two-dose schedule and 15% buffer and wastage (resulting in 0.85 billion vaccine courses available). FVP: fully vaccinated persons.*

**Allocation strategy**		**Income setting**	**Deaths averted per million**	**Deaths averted per 100 FVP**	**Total deaths averted per million global population**	**Total deaths averted per 100 FVP**
Allocated to limited countries at 80% coverage	A: Countries receive doses in proportion to population	HIC	450	0.4	348	0.31
		UMIC	300	0.27		
		LMIC	346	0.31		
		LIC	378	0.34		
	B: Countries receive doses in proportion to population, with 65+ group prioritised and remaining doses allocated to 15-64 age groups	HIC	1293	1.16	1316	1.18
		UMIC	1268	1.14		
		LMIC	1388	1.25		
		LIC	1238	1.11		
	C: Countries receive doses in proportion to 65+ population, with 65+ group prioritised and remaining doses allocated to 15-64 age groups	HIC	2260	0.9	1394	1.25
		UMIC	1252	1.09		
		LMIC	1279	1.91		
		LIC	932	2.56		
	D: Allocated first to high-income countries	HIC	2837	0.4	450	0.40
		UMIC	0	0		
		LMIC	0	0		
		LIC	0	0		
	E: Allocated first to low-income and lower-middle-income countries	HIC	0	0	352	0.32
		UMIC	0	0		
		LMIC	740	0.31		
		LIC	808	0.34		
	F: Countries receive doses in proportion to population, plus 1.15 b doses to HIC and 1.1 b doses to MIC	HIC	2081	0.4	785	0.33
		UMIC	519	0.27		
		LMIC	598	0.31		
		LIC	378	0.34		

Table S5: Optimised global allocation of vaccine doses for different assumptions about vaccine characteristics, transmission, health system constraints, and optimisation outcome.

**Parameter assumption**	**Income setting**	**Deaths averted per million**	**Deaths averted per 100 fully vaccinated persons**	**Total deaths averted per million global population**	**Total deaths averted per 100 fully vaccinated persons**
Default	HIC	3135	1.16	1672	1.43
	UMIC	1241	1.44		
	LMIC	1581	1.62		
	LIC	1533	1.81		
Lower vaccine efficacy (70%)	HIC	3068	0.94	1497	1.28
	UMIC	1073	1.37		
	LMIC	1365	1.61		
	LIC	1335	1.58		
Reduced vaccine efficacy (scaled by 50%) in 65+ years population	HIC	3135	1.16	1503	1.28
	UMIC	1235	1.07		
	LMIC	1202	1.73		
	LIC	1330	1.57		
Vaccine efficacious against disease only	HIC	1773	0.97	1391	1.19
	UMIC	1068	1.36		
	LMIC	1544	1.18		
	LIC	1483	1.28		
NPIs maintained at higher level following vaccine introduction (such that R_{t2}=1.5)	HIC	1551	1.37	1259	1.07
	UMIC	1131	0.99		
	LMIC	1145	1.02		
	LIC	1856	1.19		
NPIs maintained at lower level following vaccine introduction (such that R_{t2}=2.5)	HIC	3734	1.28	2331	1.99
	UMIC	1573	2.01		
	LMIC	2698	2.76		
	LIC	1659	1.96		
Health system constraints absent	HIC	3135	1.16	1417	1.21
	UMIC	1602	1.06		
	LMIC	790	1.74		
	LIC	585	1.46		
Reduced infectiousness in children younger than 10 years	HIC	3381	1.16	1730	1.48
	UMIC	1238	1.58		
	LMIC	1639	1.68		
	LIC	1549	1.83		
Life-years gained as optimisation outcome measure	HIC	3135	1.16	1532	1.31
	UMIC	593	3.52		
	LMIC	1612	1.51		
	LIC	2614	0.75		

Table S6: Sensitivity analysis for the fixed global vaccine allocation scenarios.

	**Sensitivity Analysis** Total deaths averted per million global population (Total deaths averted per 100 fully vaccinated people)							
	Default	Lower vaccine efficacy (70%)	Reduced vaccine efficacy (scaled by 50%) in 65+ years population	Vaccine efficacious against disease only	NPIs maintained at higher level following vaccine introduction (such that R_{t2}=1.5)	NPIs maintained at lower level following vaccine introduction (such that R_{t2}=2.5)	Health system constraints absent	Reduced infectiousness in children younger than 10 years
A: Countries receive doses in proportion to population	650 (0.59)	524 (0.48)	609 (0.55)	289 (0.26)	562 (0.51)	809 (0.74)	484 (0.44)	671 (0.61)
B: Countries receive doses in proportion to population, targeted first to 65+, then 15-64 age groups	1324 (1.2)	1182 (1.07)	1082 (0.98)	1118 (1.01)	756 (0.68)	1936 (1.75)	1091 (0.99)	1393 (1.26)
C: Countries receive doses in proportion to population in 65+ age group, targeted first to 65+, then 15-64 age groups	1386 (1.25)	1245 (1.12)	1135 (1.02)	1188 (1.07)	767 (0.69)	1995 (1.8)	1193 (1.07)	1461 (1.32)
D: Allocated first to high-income countries	513 (0.46)	513 (0.46)	513 (0.46)	330 (0.3)	264 (0.24)	713 (0.64)	513 (0.46)	559 (0.5)
E: Allocated first to low-income and lower-middle-income countries	597 (0.53)	484 (0.43)	571 (0.51)	319 (0.28)	635 (0.57)	908 (0.81)	331 (0.29)	610 (0.54)
F: Countries receive doses in proportion to population, plus additional 1.15 b doses to HIC and 1.1 b doses to MIC	1308 (0.56)	1103 (0.47)	1266 (0.54)	625 (0.27)	1075 (0.46)	1710 (0.73)	1098 (0.47)	1374 (0.59)

3 References

1 Walker PGT, Whittaker C, Watson OJ, *et al.* The impact of COVID-19 and strategies for mitigation and suppression in low- and middle-income countries. *Science* 2020; **422**: eabc0035.

2 Pritchard M, Dankwa EA, Hall M, *et al.* ISARIC Clinical Data Report 4 October 2020. *medRxiv* 2020; DOI: https://doi.org/10.1101/2020.07.17.20155218.

3 ICNARC. ICNARC report on COVID-19 in critical care, 16 October 2020. 2020. https://www.icnarc.org/Our-Audit/Audits/Cmp/Reports.

4 Brazeau NF, Verity R, Jenks S, *et al.* Report 34: COVID-19 Infection Fatality Ratio estimates from seroprevalence. 2020. DOI: https://doi.org/10.25561/83545.

5 Linton NM, Kobayashi T, Yang Y, *et al.* Incubation Period and Other Epidemiological Characteristics of 2019 Novel Coronavirus Infections with Right Truncation: A Statistical Analysis of Publicly Available Case Data. *J Clin Med* 2020; **9**: 538.

6 Li Q, Guan X, Wu P, *et al.* Early Transmission Dynamics in Wuhan, China, of Novel Coronavirus–Infected Pneumonia. *N Engl J Med* 2020; **382**: 1199–207.

7 Lauer SA, Grantz KH, Bi Q, *et al.* The Incubation Period of Coronavirus Disease 2019 (COVID-19) From Publicly Reported Confirmed Cases: Estimation and Application. *Ann Intern Med* 2020; **172**: 577–82.

8 Bi Q, Wu Y, Mei S, *et al.* Epidemiology and transmission of COVID-19 in 391 cases and 1286 of their close contacts in Shenzhen, China: a retrospective cohort study. *Lancet Infect Dis* 2020; **20**: 911–9.

9 Docherty AB, Harrison EM, Green CA, *et al.* Features of 20 133 UK patients in hospital with covid-19 using the ISARIC WHO Clinical Characterisation Protocol: Prospective observational cohort study. *BMJ* 2020; **369**: 1–12.

10 Hawryluk I, Mellan TA, Hoeltgebaum HH, *et al.* Inference of COVID-19 epidemiological distributions from Brazilian hospital data. *J R Soc Interface* 2020; **17**: 20200596.

11 South African COVID-19 Modelling Consortium. Estimating cases for COVID-19 in South Africa. Short term Projections: June 2020. 2020 https://www.nicd.ac.za/wp-content/uploads/2020/06/SACovidModellingReport_ShortTermProjections_12062020_Final2.pdf.

12 Sreevalsan-Nair J, Vangimalla RR, Ghogale PR. Analysis and estimation of length of in-hospital stay using demographic data of COVID-19 recovered patients in Singapore. *medRxiv* 2020; DOI: https://doi.org/10.1101/2020.04.17.20069724.

13 Haw NJL, Uy J, Sy KTL, Abrigo MRM. Epidemiological profile and transmission dynamics of COVID-19 in the Philippines. *Epidemiol Infect* 2020; **148**: e204.

14 Oliveira E, Parikh A, Lopez-Ruiz A, *et al.* ICU outcomes and survival in patients with severe COVID-19 in the largest health care system in Central Florida. *PLoS ONE* 2021; **16**(3): e0249038.

15 Salje H, Kiem CT, Lefrancq N, *et al.* Estimating the burden of SARS-CoV-2 in France. *Science* 2020; **369**: 208–11.

16 To KKW, Hung IF, Ip JD, *et al.* COVID-19 re-infection by a phylogenetically distinct SARS-coronavirus-2 strain confirmed by whole genome sequencing. *Clin Infect Dis* 2020; ciaa1275.

17 Ministerio de Ciencia e Innovaciaon Gobierno de Espana, Ministerio de Sanidad Gobierno de Espana, Consejo Interterritorial, Instituto de Salud Carlos III. Estudio Nacional de Sero-Epidemiolgia de la Infeccion por SARS-CoV-2 en Espana: Informe Final. 2020 https://www.mscbs.gob.es/ciudadanos/ene-covid/.

18 Hall V, Foulkes S, Charlett A, *et al.* Do antibody positive healthcare workers have lower SARS-CoV-2 infection rates than antibody negative healthcare workers? Large multi-centre prospective cohort study (the SIREN study), England: June to November 2020. *medRxiv* 2021; DOI: https://doi.org/2021.01.13.21249642.

19 Hansen CH, Michlmayr D, Gubbels SM, Mølbak K, Ethelberg S. Articles Assessment of protection against reinfection with SARS-CoV-2 among 4 million PCR-tested individuals in Denmark in 2020 : a population-level observational study. *Lancet* 2021; **6736**: 1–9.

20 Dan JM, Mateus J, Kato Y, *et al.* Immunological memory to SARS-CoV-2 assessed for up to 8 months after infection. *Science* 2021; **371**. DOI:10.1126/science.abf4063.

21 L.A. Jackson, E.J. Anderson, N.G. Rouphael, P.C. Roberts, M. Makhene, R.N. Coler, M.P. McCullough, J.D. Chappell, M.R. Denison, L.J. Stevens, A.J. Pruijssers, A. McDermott, B. Flach, N.A. Doria-Rose KSC, K.M. Morabito, S. O'Dell, S.D. Schmidt, P.A. Swanson II, M. Padilla, J.R. Mascola, K.M. Neuzil, H. Bennett, W. Sun, E. Peters, M. Makowski, J. Albert KC, W. Buchanan, R. Pikaart-Tautges, J.E. Ledgerwood, B.S. Graham, and J.H. Beigel for the mRNA-1273 SG. An mRNA Vaccine against SARS-CoV-2 — Preliminary Report. *N Engl J Med* 2020; **383**: 1920–31.

22 Folegatti PM, Ewer KJ, Aley PK, *et al.* Articles Safety and immunogenicity of the ChAdOx1 nCoV-19 vaccine against SARS-CoV-2 : a preliminary report of a phase 1 / 2 , single-blind , randomised controlled trial. *Lancet* 2020; **396**: 467–78.

23 Zhu FC, Li YH, Guan XH, *et al.* Safety, tolerability, and immunogenicity of a recombinant adenovirus type-5 vectored COVID-19 vaccine: a dose-escalation, open-label, non-randomised, first-in-human trial. *Lancet* 2020; **395**: 1845–54.

24 Mulligan MJ, Lyke KE, Kitchin N, *et al.* Phase I/II study of COVID-19 RNA vaccine BNT162b1 in adults. *Nature* 2020; **586**: 589–93.

25 Sahin U, Muik A, Derhovanessian E, *et al.* COVID-19 vaccine BNT162b1 elicits human antibody and TH1 T cell responses. *Nature* 2020; **586**: 594–9.

26 World Health Organization. WHO Target Product Profiles for COVID-19 Vaccines. Version 3. Geneva, 2020; https://www.who.int/publications/m/item/who-target-product-profiles-for-covid-19-vaccines.

27 Voysey M, Clemens SAC, Madhi SA, *et al.* Safety and efficacy of the ChAdOx1 nCoV-19 vaccine (AZD1222) against SARS-CoV-2: an interim analysis of four randomised controlled trials in Brazil, South Africa, and the UK. *Lancet* 2020; **397**: 99–111.

28 Voysey M, Costa Clemens SA, Madhi SA, *et al.* Single-dose administration and the influence of the timing of the booster dose on immunogenicity and efficacy of ChAdOx1 nCoV-19 (AZD1222) vaccine: a pooled analysis of four randomised trials. *Lancet* 2021; **19**: 881–91.

29 Weekes M, Jones NK, Rivett L, *et al.* Single-dose BNT162b2 vaccine protects against asymptomatic SARS-CoV-2 infection. *eLife* 2021; **10**: e68808.

30 Polack FP, Thomas SJ, Kitchin N, *et al.* Safety and Efficacy of the BNT162b2 mRNA Covid-19 Vaccine. *N Engl J Med* 2020; **383**: 2603–15.

31 Baden LR, El Sahly HM, Essink B, *et al.* Efficacy and Safety of the mRNA-1273 SARS-CoV-2 Vaccine. *N Engl J Med* 2020; **384**: 403–16.

32 Logunov DY, Dolzhikova I V, Shcheblyakov D V, *et al.* Safety and efficacy of an rAd26 and rAd5 vector-based heterologous prime-boost COVID-19 vaccine: an interim analysis of a randomised controlled phase 3 trial in Russia. *Lancet* 2021; **397**: 671–81.

33 Public Health England. PHE monitoring of the early impact and effectiveness of COVID-19 vaccination in England, 22 February 2021. 2021; 1–17. https://www.gov.uk/government/publications/phe-monitoring-of-the-effectiveness-of-covid-19-vaccination.

34 Bernal JL, Andrews N, Gower C, *et al.* Early effectiveness of COVID-19 vaccination with BNT162b2 mRNA vaccine and ChAdOx1 adenovirus vector vaccine on symptomatic disease , hospitalisations and mortality in older adults in England. *medRxiv* 2021; DOI: https://doi.org/10.1101/2021.03.01.21252652.

35 Amit S, Regev-Yochay G, Afek A, Kreiss Y, Leshem E. Early rate reductions of SARS-CoV-2 infection and COVID-19 in BNT162b2 vaccine recipients. *Lancet* 2021; **397**: 875–7.

36 Hall V, Foulkes S, Saei A, *et al.* Effectiveness of BNT162b2 mRNA Vaccine Against Infection and COVID-19 Vaccine Coverage in Healthcare Workers in England, Multicentre Prospective Cohort Study (the SIREN Study). *SSRN Prepr* 2021; https://papers.ssrn.com/sol3/papers.cfm?abstract_id=3790399.

37 MRC Centre for Global Infectious Disease Analysis Imperial College London. Future scenarios of the healthcare burden of COVID-19 in Low- or Middle-income countries. 2021. https://mrc-ide.github.io/global-lmic-reports/ (accessed Jan 26, 2021).

38 Dong E, Du H, Gardner L. An interactive web-based dashboard to track COVID-19 in real time. *Lancet Infect Dis* 2020; **20**: 533–4.

39 Worldometer. Coronavirus updates. https://www.worldometers.info/ (accessed Jan 26, 2021).
